# Multifaceted Applications of Microbial Pigments: Current Knowledge, Challenges and Future Directions for Public Health Implications

**DOI:** 10.3390/microorganisms7070186

**Published:** 2019-06-28

**Authors:** Chatragadda Ramesh, Nambali Valsalan Vinithkumar, Ramalingam Kirubagaran, Chidambaram Kulandaisamy Venil, Laurent Dufossé

**Affiliations:** 1 National Centre for Coastal Research (NCCR), NCCR Field Office, Ministry of Earth Sciences (MoES), Mandapam Camp 623519, India; 2 Atal Centre for Ocean Science and Technology for Islands, ESSO-NIOT, Dollygunj, Port Blair, Andaman and Nicobar Islands 744103, India; 3 Marine Biotechnology Group, ESSO-National Institute of Ocean Technology (NIOT), Ministry of Earth Sciences (Govt. of India), Chennai 600100, India; 4 Anna University, Department of Biotechnology, Coimbatore 641046, India; 5 Laboratoire de Chimie des Substances Naturelles et des Sciences des Aliments–LCSNSA EA 2212, Université de La Réunion, ESIROI Agroalimentaire, 97744 Saint-Denis, France

**Keywords:** microbial pigments, pigment compounds, food colorants, bioactive pigment molecules, pigment applications

## Abstract

Microbial oddities such as versatile pigments are gaining more attention in current research due to their widely perceived applications as natural food colorants, textiles, antimicrobial activities, and cytotoxic activities. This indicates that the future generation will depend on microbial pigments over synthetic colorants for sustainable livelihood. Although several reviews have detailed the comprehensive applications of microbial pigments extensively, knowledge on several aspects of pigmented microbes is apparently missing and not properly reviewed anywhere. Thus, this review has been made to provide overall knowledge on biodiversity, distribution, pathogenicity, and ecological and industrial applications of microbial pigments as well as their challenges and future directions for food, industrial, and biomedical applications. Meticulously, this compendious review treatise on the pigments from bacteria, fungi, yeasts, and microalgae includes reports from the 1970s to 2018. A total of 261 pigment compounds produced by about 500 different microbial species are included, and their bioactive nature is described.

## 1. Introduction

Microbial communities have an enormous potentiality to produce diverse and mesmerizing aesthetic traits, such as knack emission of bioluminescence and fluorescence, formation of magnetosomes, production of bioactive metabolites, and different pigments for scientific succulence. Colloquially, directly or indirectly, microbial communities play an important integrated role in the biosphere by regulating biogeochemical and ecological processes [1]. Regardless of their role in the environment, they offer several benefits to humanity; one such benefit is pigment production by several microbes, of which deserved importance is being highlighted in recent times, and there are still more untapped sources to explore many unknown pigmented compounds [2]. The importance of microbial pigments has been emphasized in different applications, such as cosmetics, food, pharmaceuticals, and textiles, and these compounds are also well-known to exhibit cytotoxic, antioxidant, antimicrobial, antimalarial, anticancer, antitumor, and antifouling activities [3,4,5,6,7].

Pigments are molecules that absorb a specific wavelength of light and reflect the rest of the pulchritude visible spectrum (380–750 nm). Pigment production is one of the charismatic traits of microbes. Apparently, microbial pigments are not merely colors, but they possess a mixture of diverse chemical components with multifaceted potential biological activities [8]. In the last two decades, studies on pigmented microorganisms from terrestrial and marine ecosystems have tremendously expanded, resulting in the use of pigments in cancer-related research.

Microbial pigmented molecules such as bacteriochlorophylls, carotenoids, flavins, indigoids, melanins, pheomelanin, monascins, phenazines, phenazostatin D, prodigiosin, quinone precursors, violacein, glaukothalin, pycocyanin, xanthomonadin, phenazine, canthaxanthin, astaxanthin, β-carotene, etc. are produced as biproducts by several microorganisms [3,9,10,11,12]. Many of these compounds and their derivatives are reported to show wide range of cell specific biological activities which are expressed in effective/inhibitory/lethal/subleathal concentrations such as effective dose (ED), growth inhibitory concentration (GIC), minimum inhibitory concentration (MIC), Half maximal effective concentration (EC_50_), half maximal growth inhibition (GI_50_), half maximal inhibitory concentration (IC_50_), half maximal lethal concentration (LC_50_), half maximal lethal dose (LD_50_), and tumour growth inhibition of 50% (TGI_50_) [13]. 

There are several synthetic colorants that are being developed as immunosuppressive and anticancer drugs [14]. Historical notes on several synthetic dyes and pigments are of industrial applications (textile, cosmetics, and food), and their disadvantages have recently been well-detailed [15,16]. Since a number of synthetic pigments and their biproducts are found to display toxic, teratogenic, and carcinogenic properties, the exploration of natural pigments from microbes has emerged in recent years due to their biodegradability than synthetic counterparts [17]. Several natural pigment compounds originated from different sources are being used as food colorants [18]. Irrespective of plant, animal, and synthetic pigments, microbial pigments are much mellower and highly preferred due to their higher productivity and optimizable culture conditions. Pigment productions by *Monascus*, *Rhodotorula*, marine actinomycetes, marine *Pseudoalteromonas*, and marine cyanobacterial species have been widely studied. In this context, this review appends a plethora of industrially important pigmented molecules produced by different microorganisms such as bacteria, fungi, cyanobacteria, and yeast.

## 2. Microbial Pigments and Chemical Structures

Microbial pigments are usually seen in two forms as pigments diffused out into the media and pigments retained within the cells. To produce any kind of pigmented compound with potential biological properties from any organism for industrial applications, the following requirements need to be fulfilled: Organisms should be amenable to culture, have a fast growth rate, be optimizable, have a high productivity in limited space and in short time, be available and able to produce throughout the year, be nontoxigenic, be nonpathogenic, be able to grow in a wide range of nutrients such as carbon and nitrogen sources, and be tolerant to a broad spectrum of physical (light and temperature) and chemical (pH and osmolarity) parameters used in production. Mostly, these requirements are met with microbes; hence, many research studies have been prioritized and focused on microbes (particularly marine bacteria are highly preferred due to their higher productivity and optimizable culture conditions) as potential sources over plants, animals, and synthetic compounds. Further inclination towards optimization and improvement of mass production of pigments are encouraged with genetic engineering as well. 

There are four main sources of pigments for various applications as mentioned above: (1) Plant-derived pigments, (2) animal-derived pigments, (3) microbial pigments, and (4) synthetic pigments. Here, merely microbial pigments are reviewed due to their insatiable demand over the rest of the sources and for better understanding on the same aspect. Some of the carotenoid pigments, such as acetylenic carotenoids appear to be restricted to certain environments, e.g., some marine bacteria *Planococcus maritimus* and *Rubritalea squalenifaciens* biosynthesize acyclic C_30_-type carotenoic acids [19]. In nature, C_50_ Carotenoids such as sarcinaxanthin and decaprenoxanthin are exclusively biosynthesized by *Halobacteria*, *Halococcus*, *Actinomycetales*, *Flavobacterium dehydrogenans*, *Arthrobacter* sp., *Micrococcus luteus*, *Dietzia* sp., *Corynebacterium poinsettiae*, *C. glutamicum*, and a strain of *Pseudomonas*, and these carotenoids appear to be not produced by plants [20,21]. Similarly, aryl carotenoids such as isorenieratene, 3-hydroxy-isorenieratene and 3,3′-di-hydroxy-isorenieratene are found in very few microorganisms, such as *Brevibacterium linens, Streptomyces mediolani*, and *Mycobacterium aurum* [21]. Identifying metabolic pathways and genes responsible for such rare phenomena are of great importance for genetic engineering studies to develop rare carotenoids with therapeutic application. 

**Figure 1:**
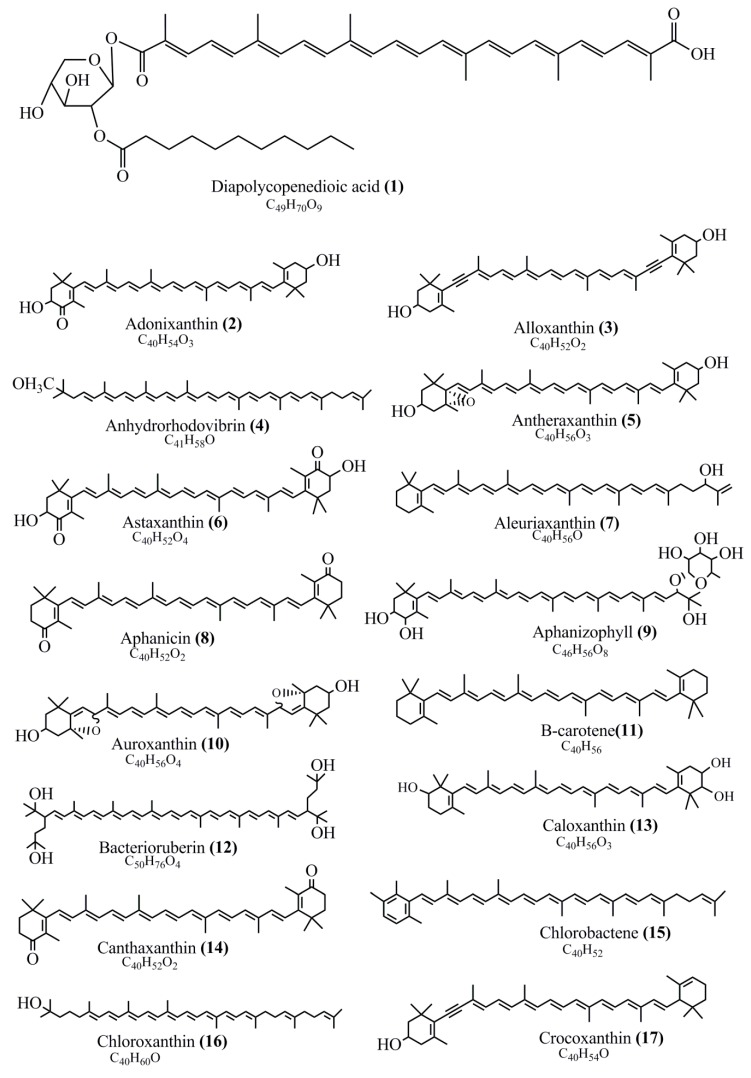

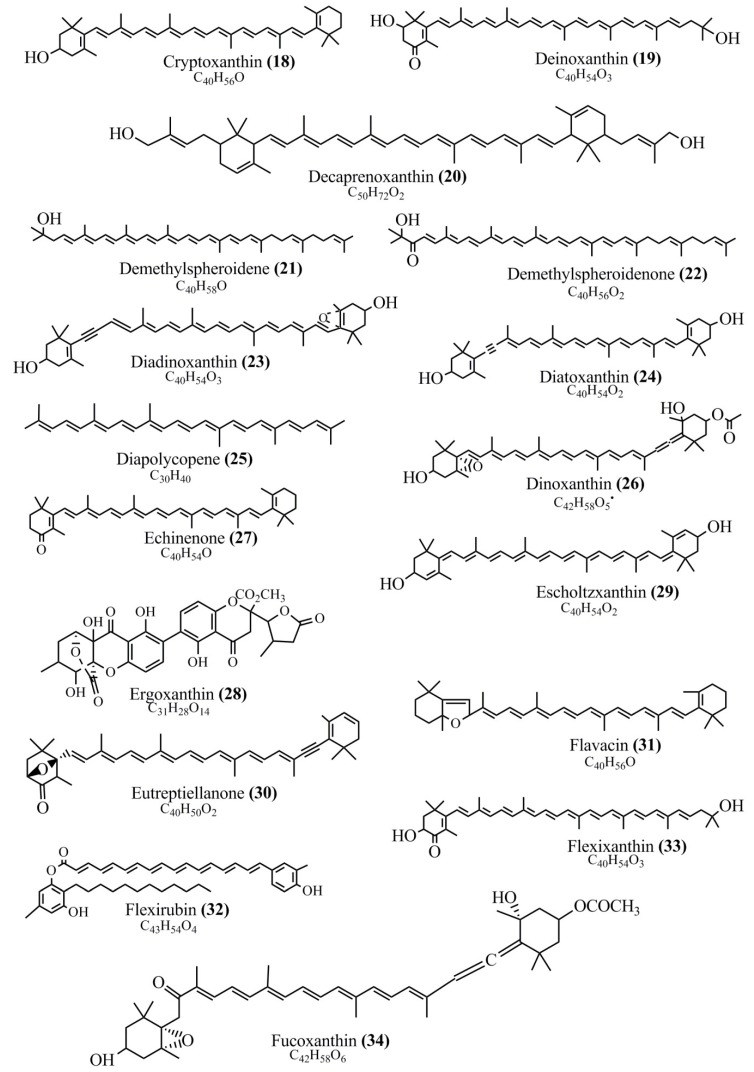

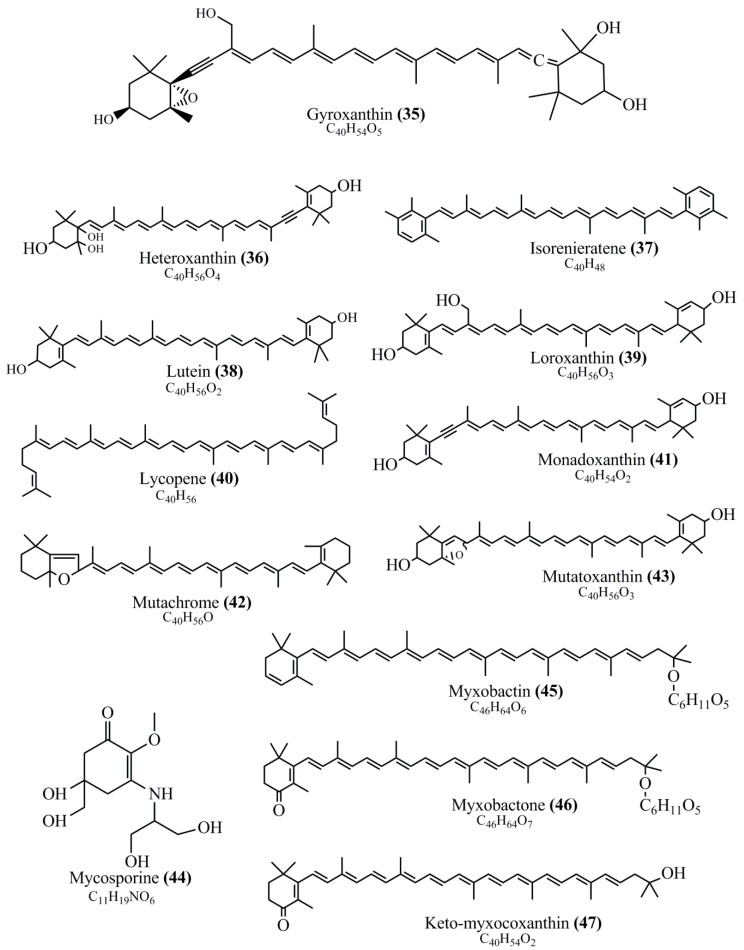

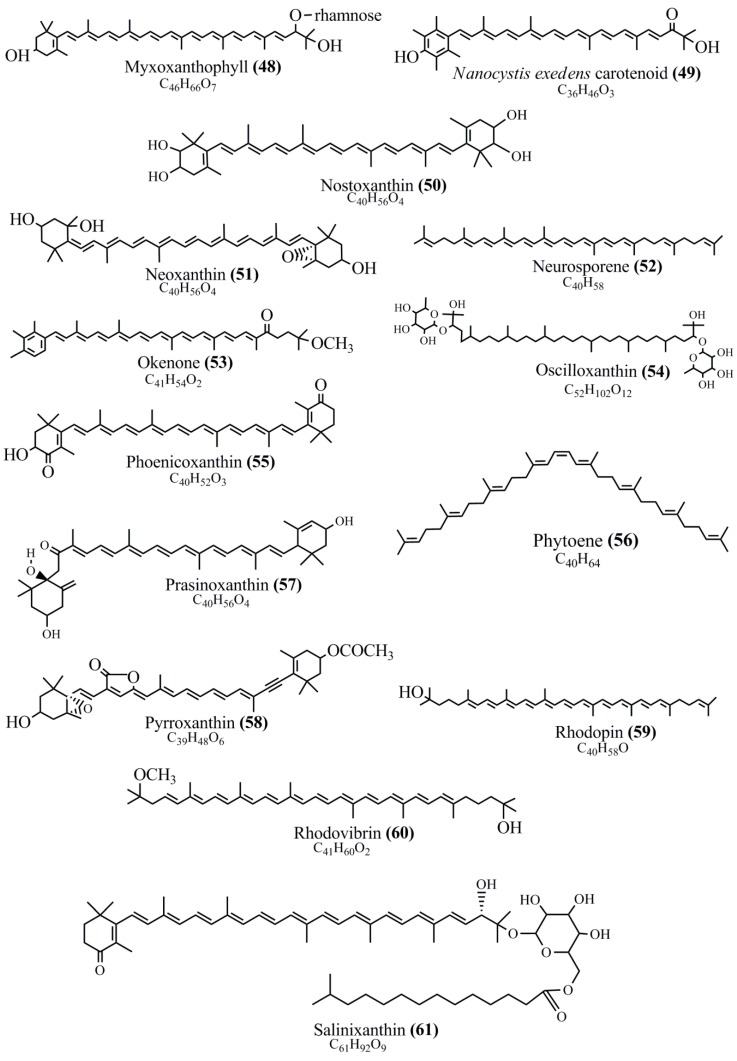

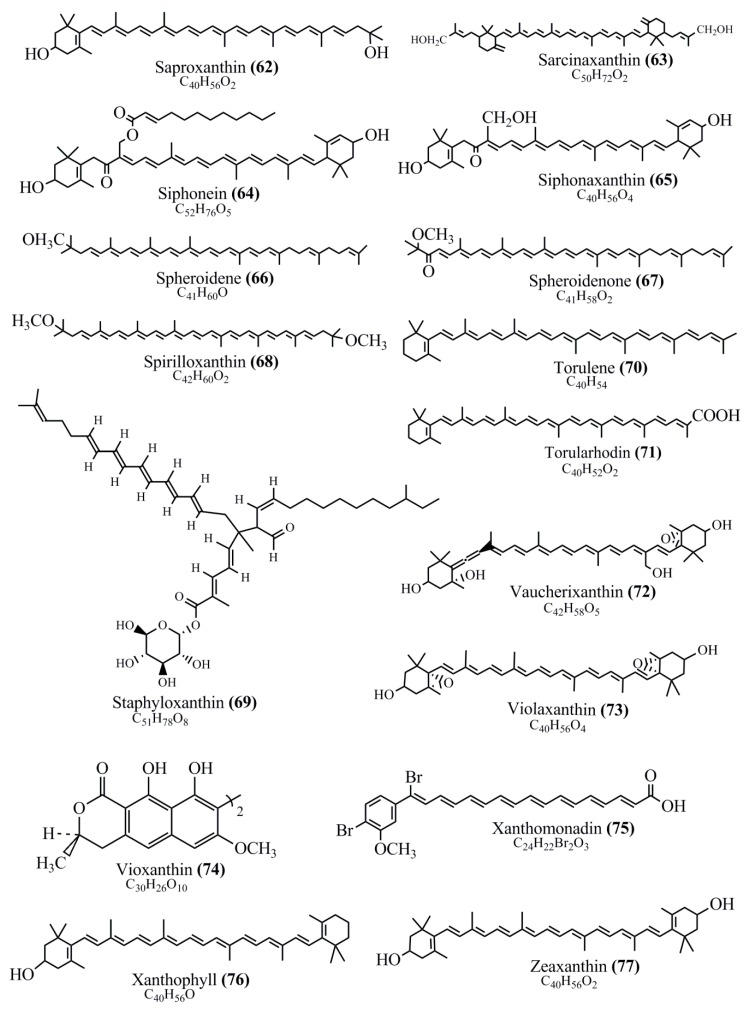

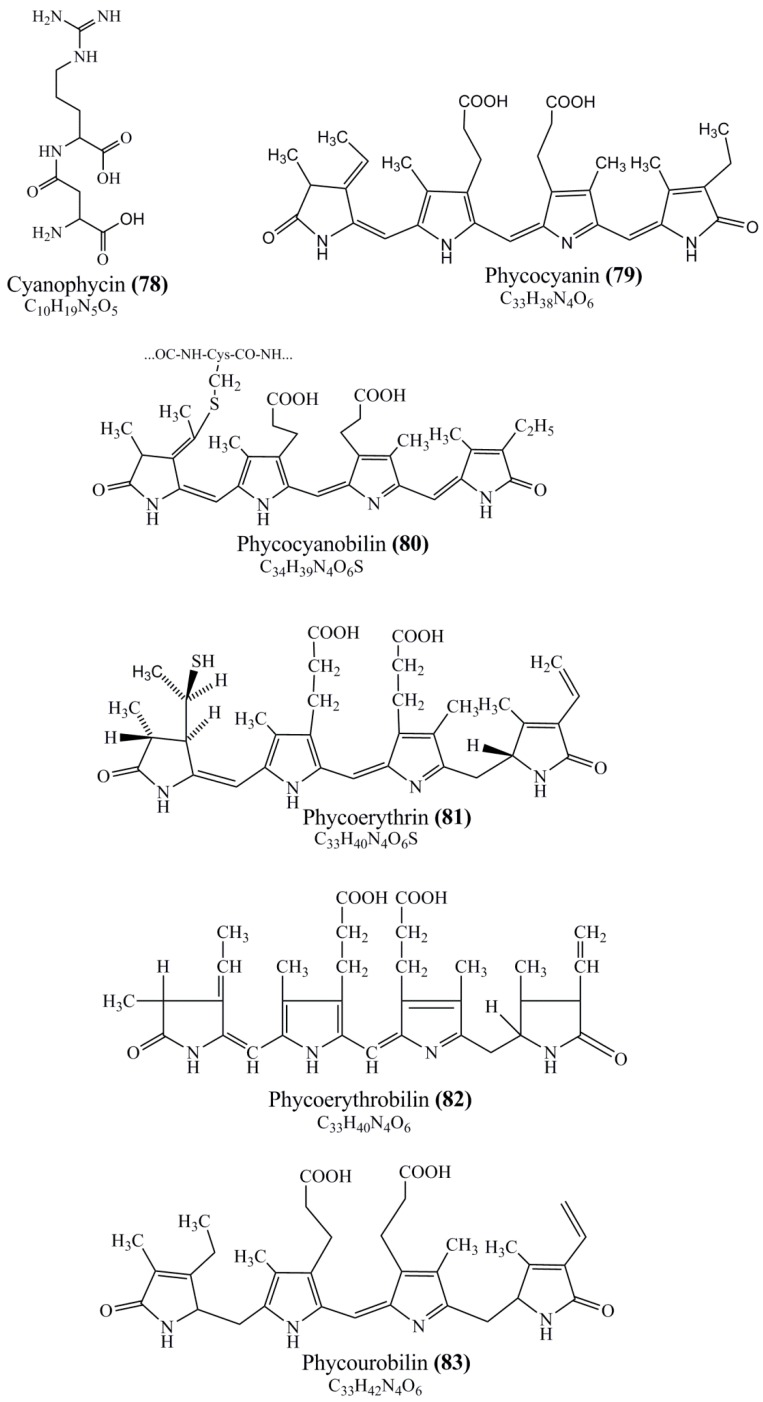

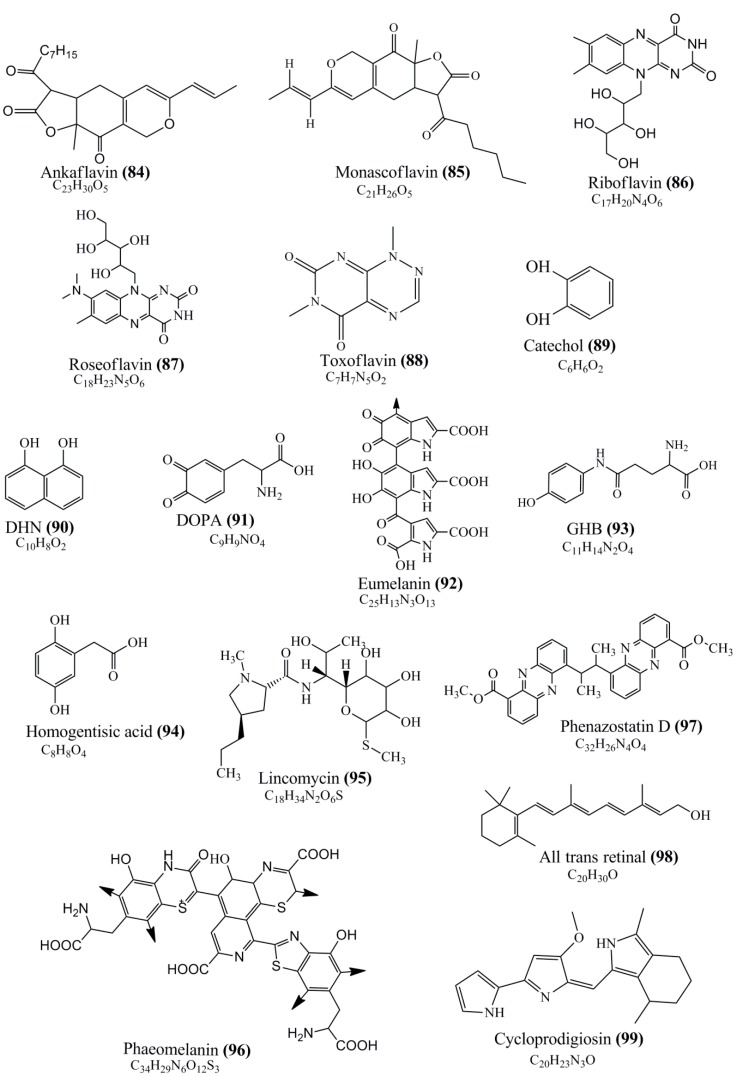

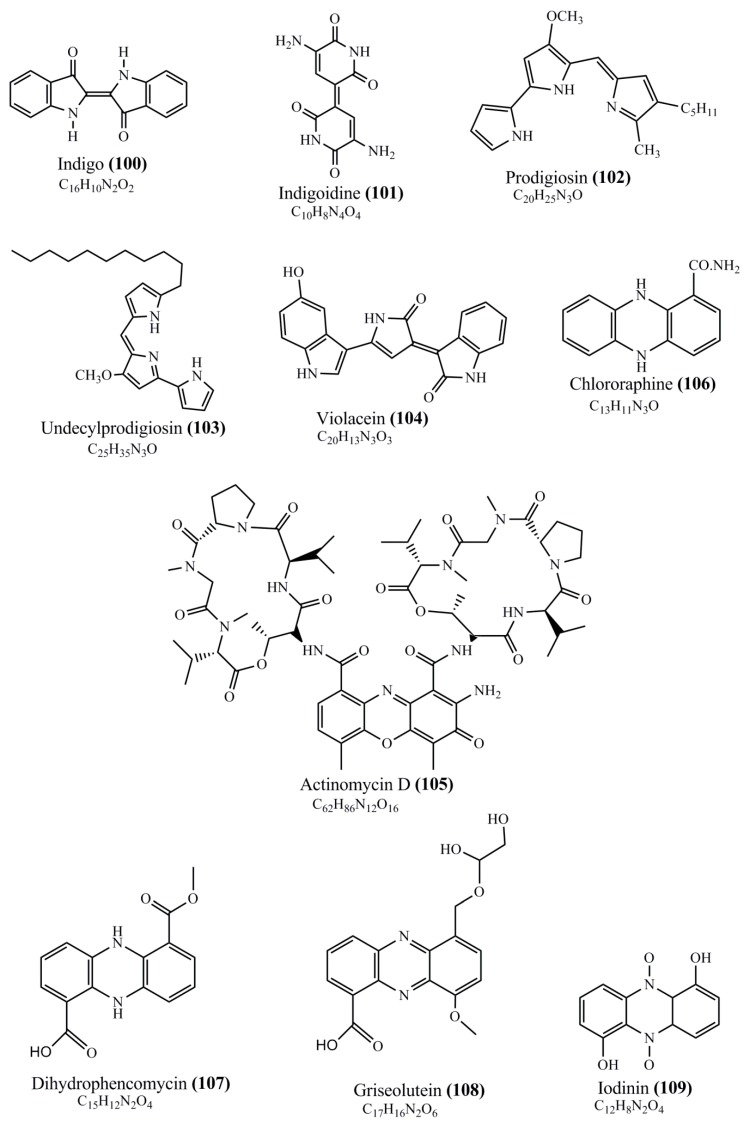

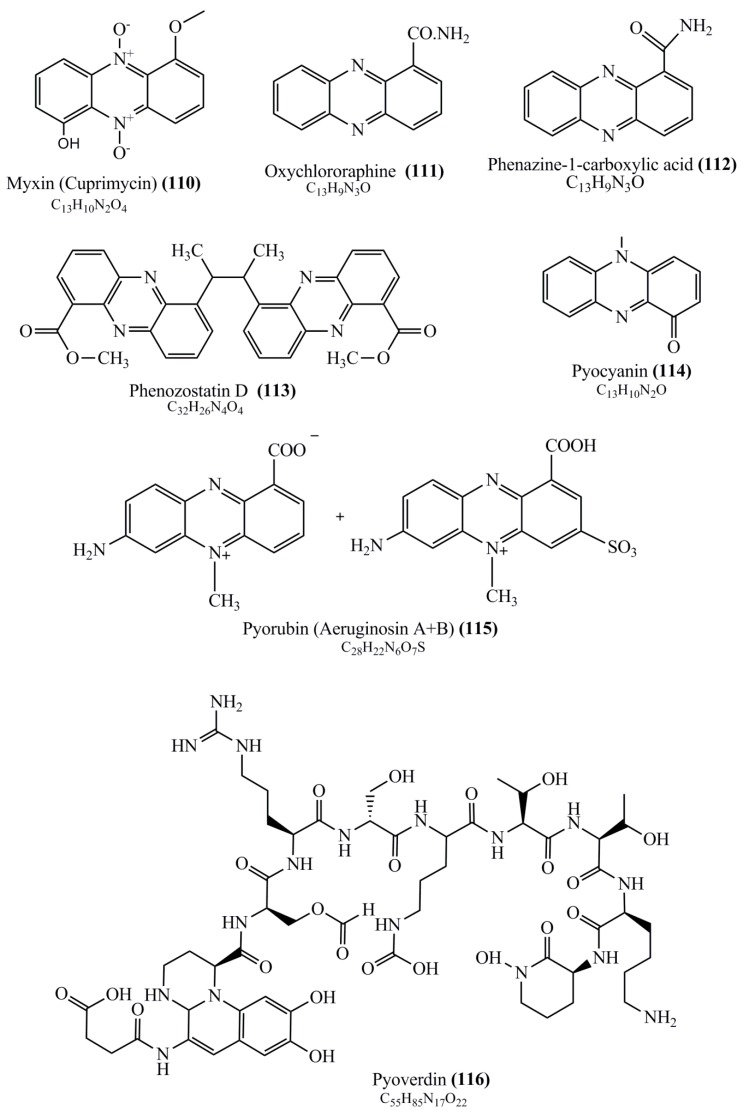

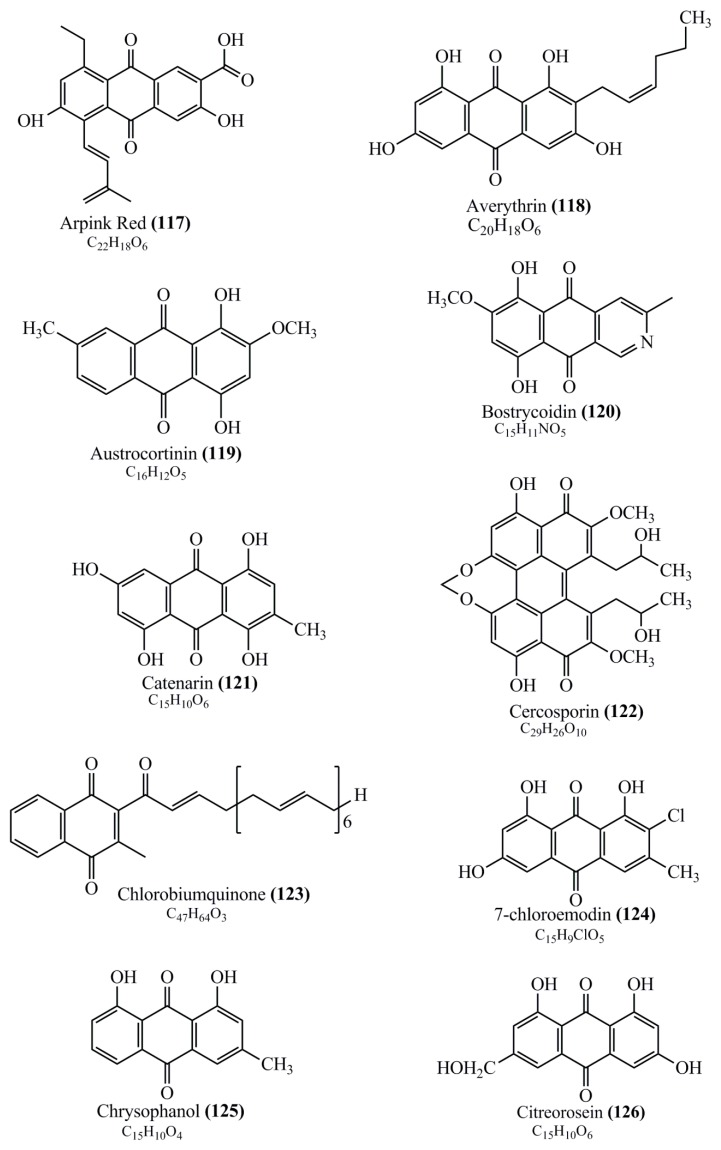

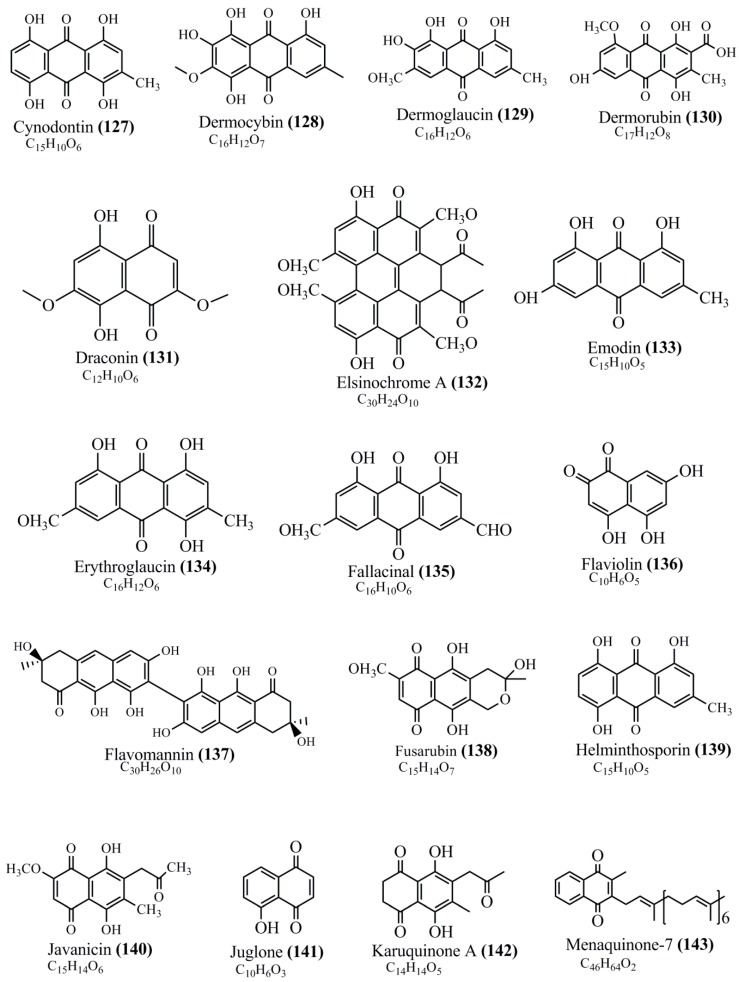

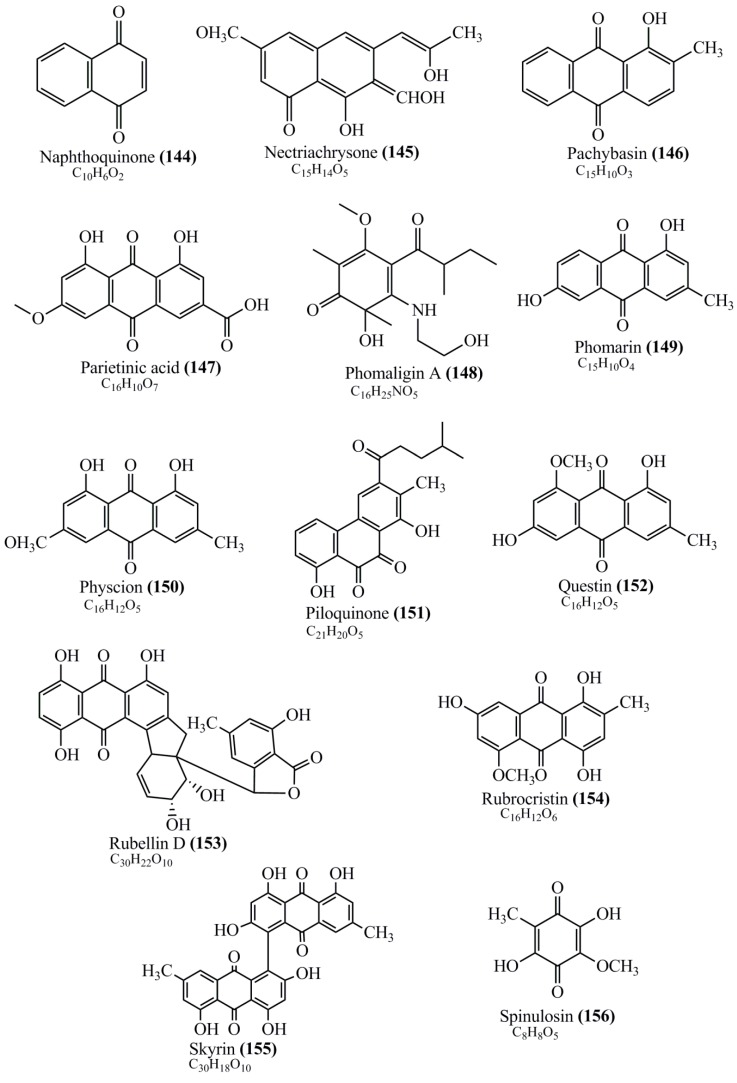

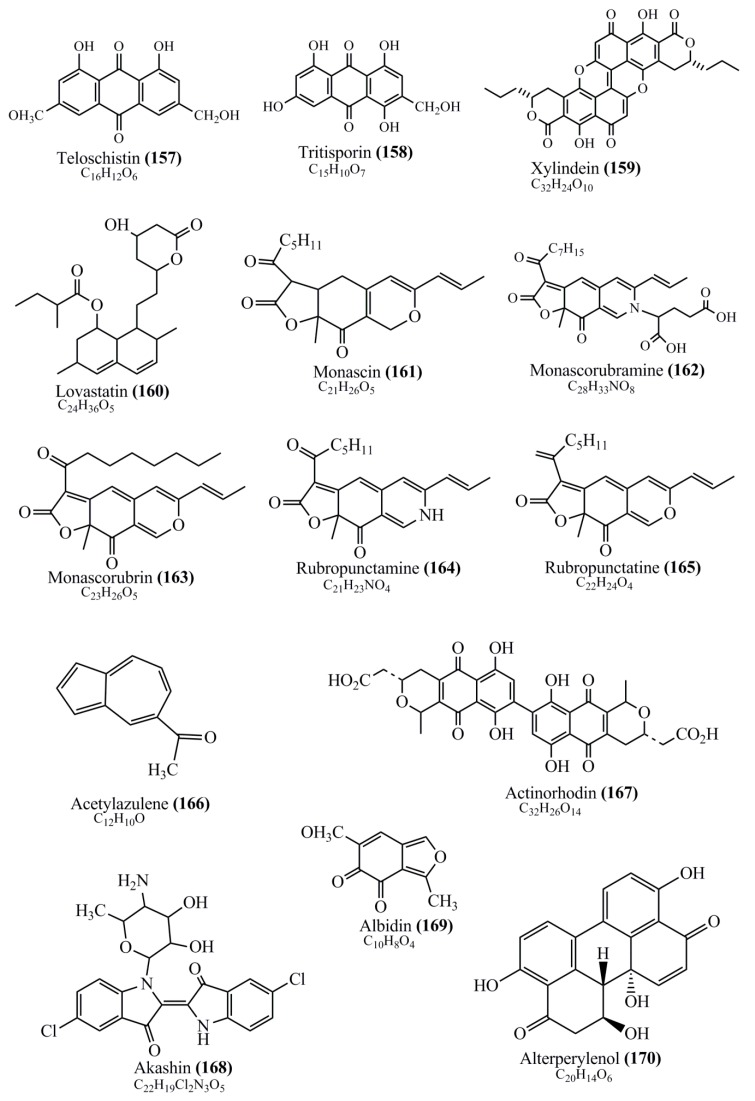

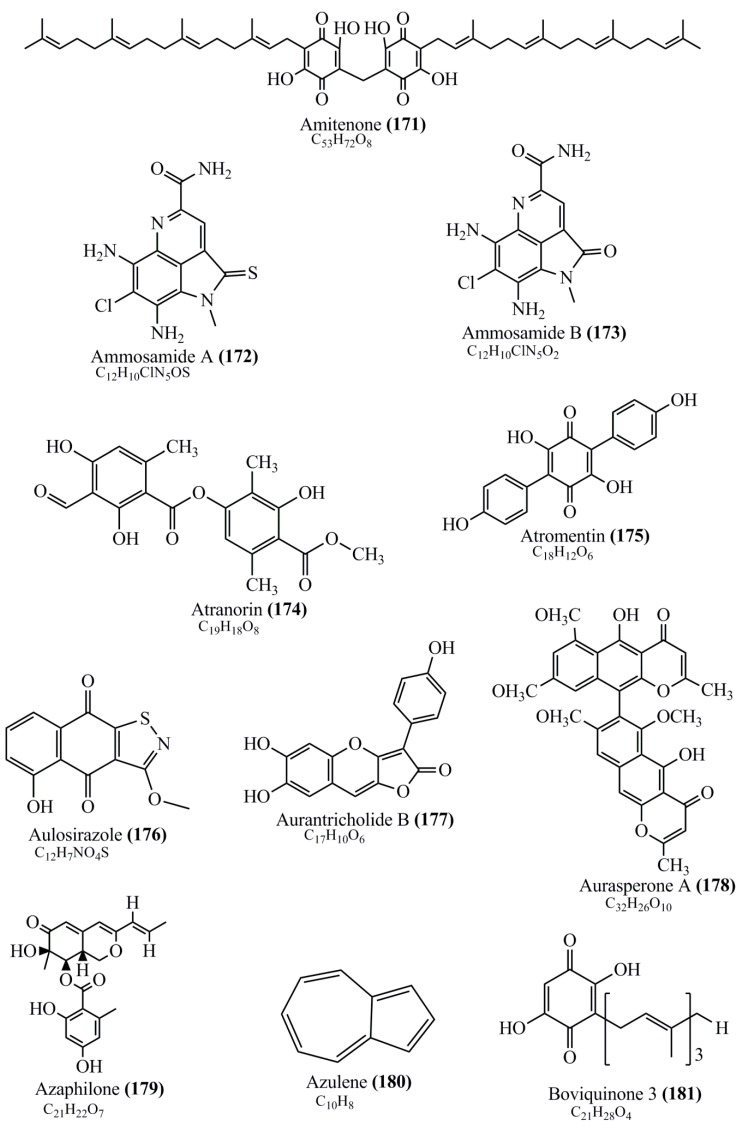

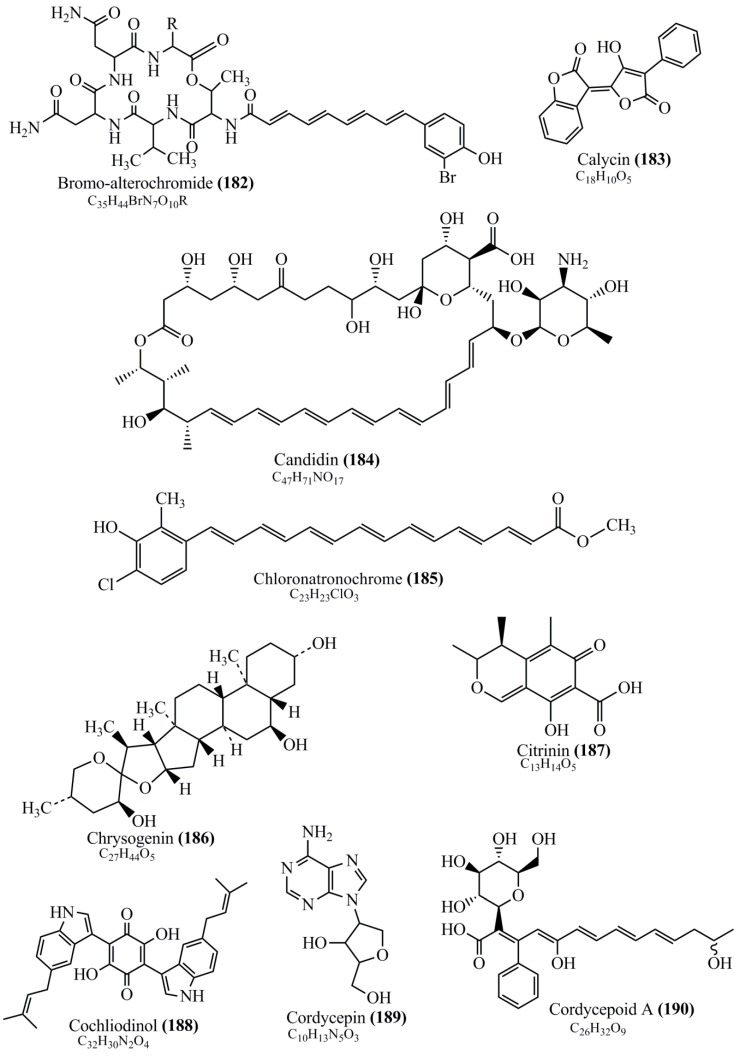

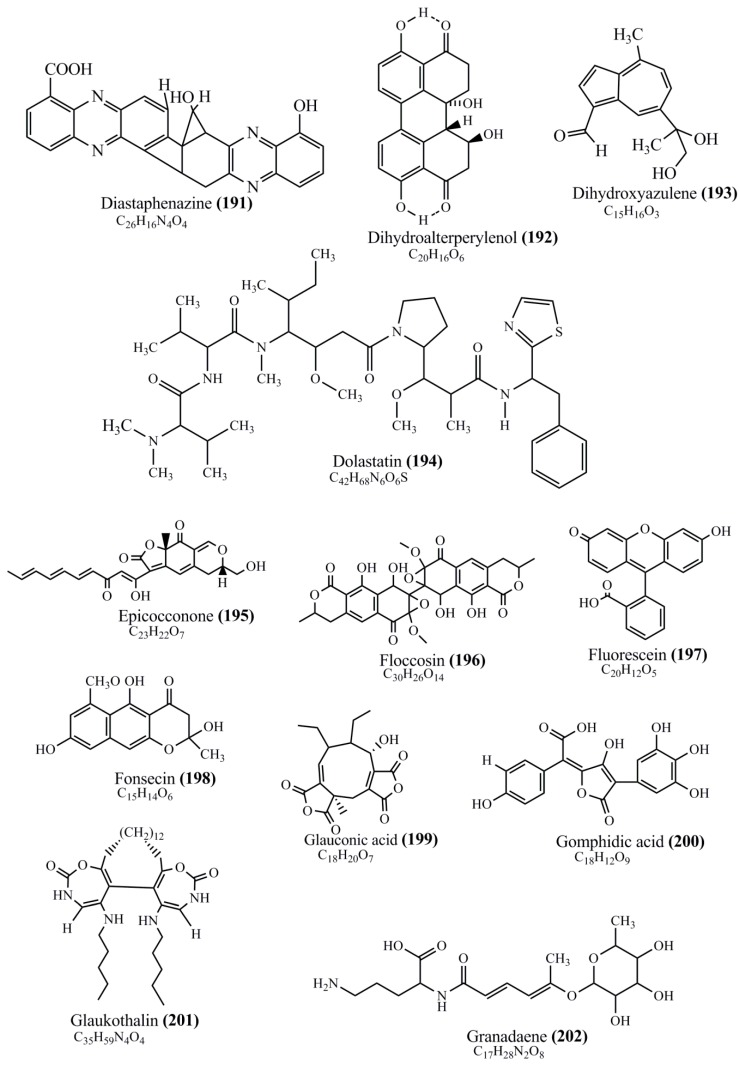

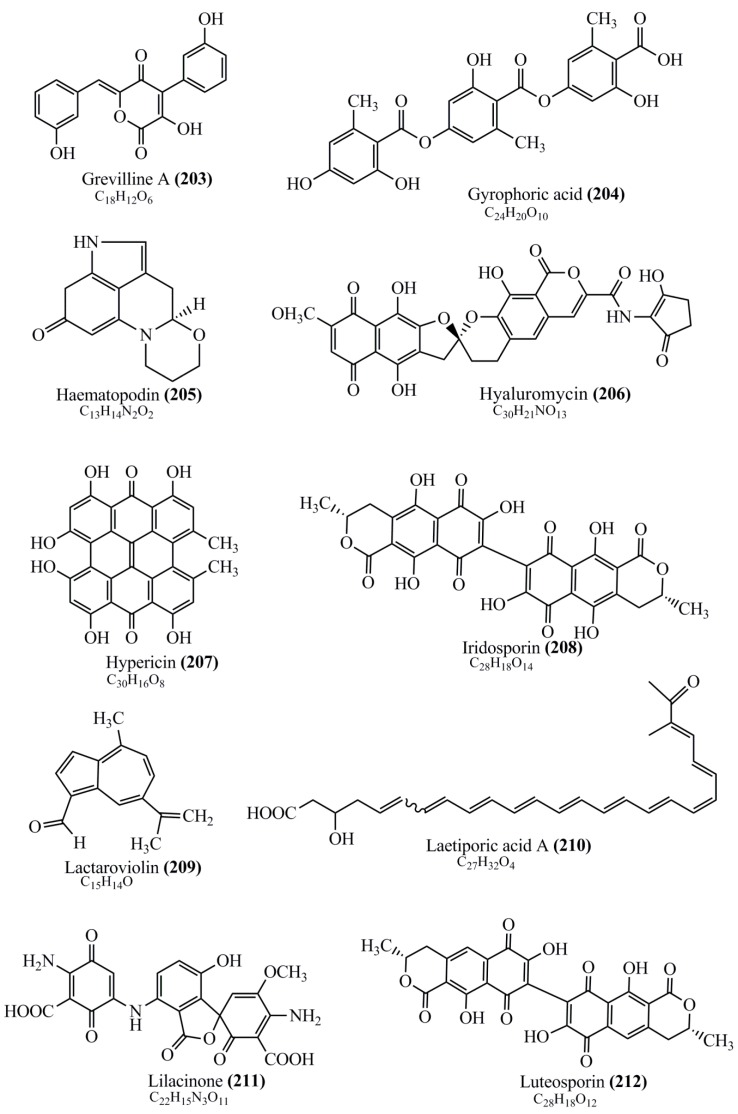

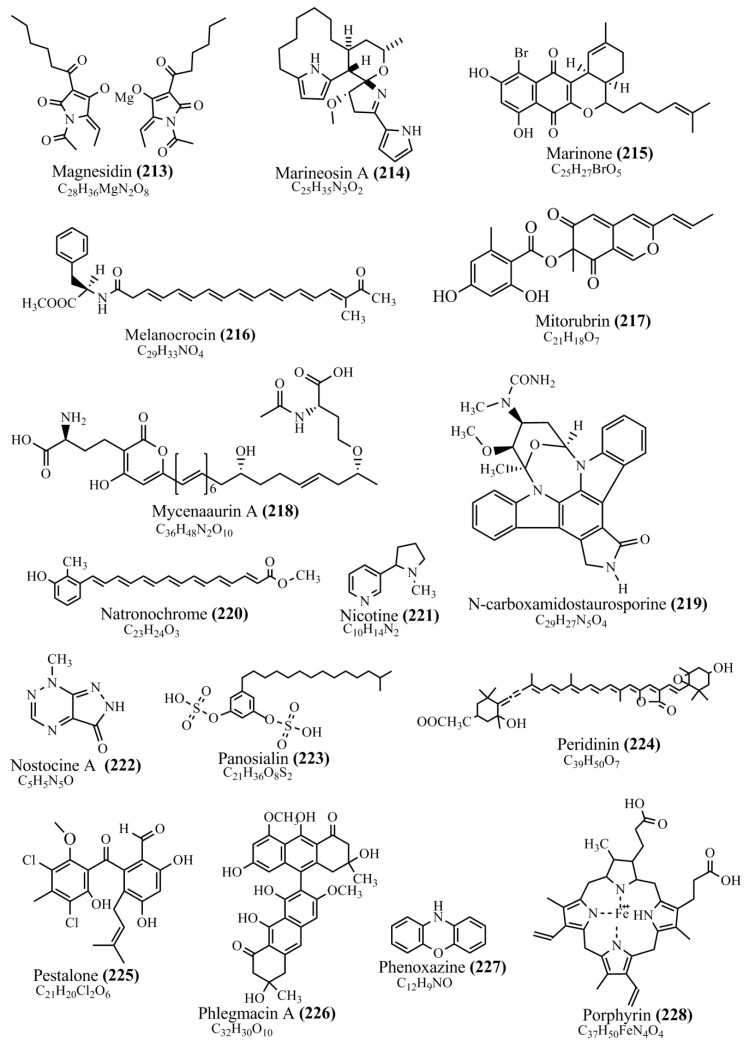

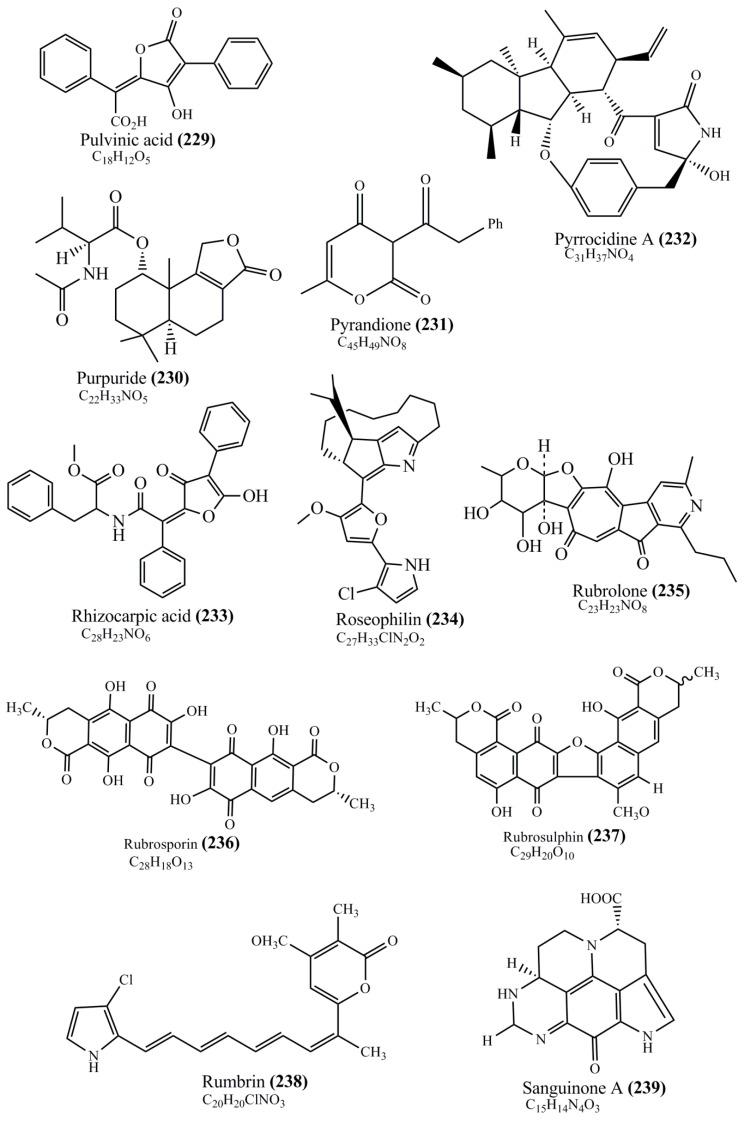

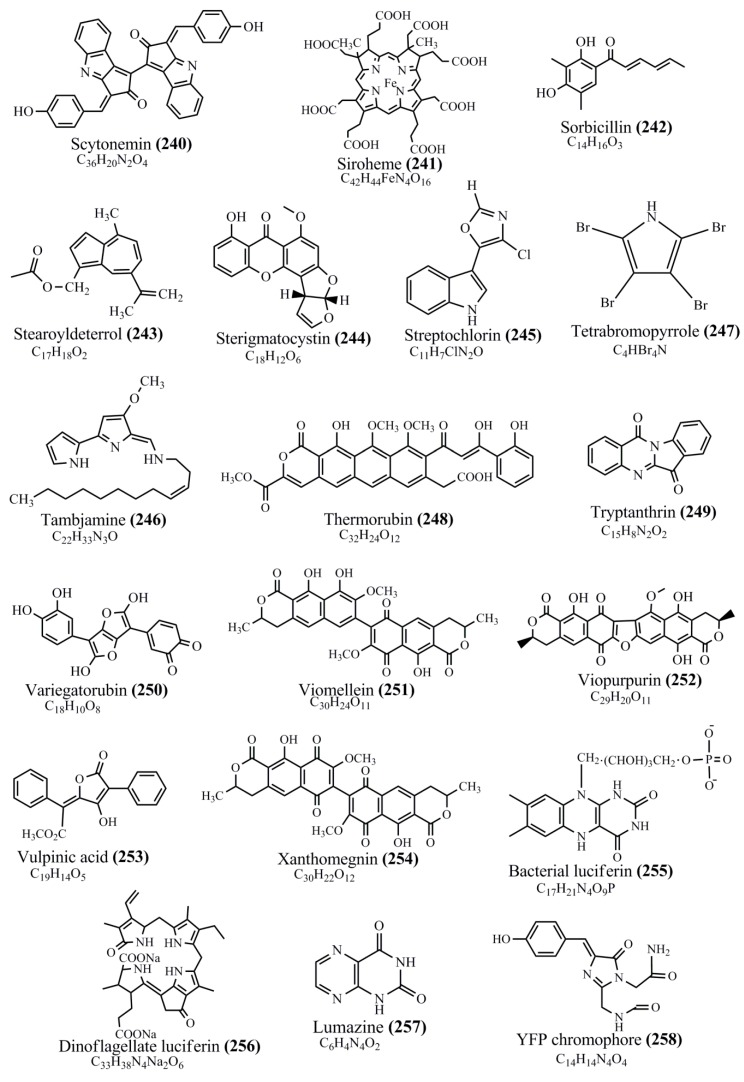

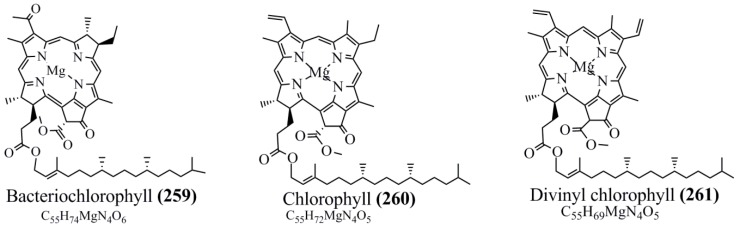
Chemical structures of various pigments.

The chemical structures of some of the major pigment molecules are given below: carotenoids: Acyl glyco-carotenoic acid (diapolycopenedioic acid) **(1)**, adonixanthin **(2)**, alloxanthin **(3),** anhydrorhodovibrin **(4),** antheraxanthin **(5)**, astaxanthin **(6)**, aleuriaxanthin **(7)**, aphanicin **(8)**, aphanizophyll **(9)**, auroxanthin **(10)**, β-carotene **(11)**, bacterioruberin **(12)**, caloxanthin **(13)**, canthaxanthin **(14)**, chlorobactene **(15)**, chloroxanthin **(16)**, crocoxanthin **(17)**, cryptoxanthin **(18)**, deinoxanthin **(19)**, decaprenoxanthin **(20)**, demethylspheroidene **(21)**, demethylspheroidenone **(22)**, diadinoxanthin **(23)**, diatoxanthin **(24)**, diapolycopene **(25)**, dinoxanthin **(26)**, echinenone **(27)**, ergoxanthin **(28)**, escholtzxanthin **(29)**, eutreptiellanone **(30)**, flavacin **(31)**, flexirubin **(32)**, flexixanthin **(33)**, fucoxanthin **(34)**, gyroxanthin diester **(35)**, heteroxanthin **(36)**, isorenieratene **(37)**, lutein **(38)**, loroxanthin **(39)**, lycopene **(40)**, monadoxanthin **(41)**, mutachrome **(42)**, mutatoxanthin **(43)**, mycosporine **(44)**, myxobactin **(45)**, myxobactone **(46)**, keto-myxocoxanthin **(47)**, myxoxanthophyll **(48)**, *Nanocystis exedens* pigments **(49)**, nostoxanthin **(50)**, neoxanthin **(51)**, neurosporene **(52)**, okenone **(53)**, oscilloxanthin **(54)**, phoenicoxanthin **(55)**, phytoene **(56)**, prasinoxanthin **(57)**, pyrroxanthin **(58)**, rhodopin **(59)**, rhodovibrin **(60)**, salinixanthin **(61)**, saproxanthin **(62)**, sarcinaxanthin **(63)**, siphonein **(64)**, siphonaxanthin **(65)**, spheroidene **(66)**, spheroidenone **(67)**, spirilloxanthin **(68)**, staphyloxanthin **(69)**, torulene **(70)**, torularhodin **(71)**, vaucherixanthin **(72)**, violaxanthin **(73)**, vioxanthin **(74)**, xanthomonadines **(75)**, xanthophyll **(76)**, and zeaxanthin **(77)**; phycobiliproteins: cyanophycin **(78)**, phycocyanin **(79)**, phycocyanobilin **(80)**, phycoerythrin **(81)**, phycoerythrobilin **(82)**, and phycourobilin **(83)**; flavins: ankaflavin **(84)**, monascoflavin **(85)**, riboflavin **(86)**, roseoflavin **(87)**, and toxoflavin **(88)**; melanins: melanin precursors such as catechol **(89)**, 1,8-dihydroxynaphthalene (DHN) **(90)**, Dopa **(91)**, eumelanin **(92)**, l-glutaminyl-4-hydroxybenzene (GHB) **(93)**, homogentisic acid (HGA) **(94)**, lincomycin **(95)**, phaeomelanin **(96)**, phenazostatin D **(97)**, and all trans-retinal **(98)**; heterocyclic pigments: cycloprodigiosin **(99)**, indigotine (indigo) **(100),** indigoidine **(101)**, prodigiosin **(102)**, undecylprodigiosin **(103)**, and violacein **(104)**; phenazine compounds: actinomycin D **(105)**, chlororaphine **(106)**, Dihydrophencomycin **(107)**, griseolutein **(108)**, iodinin **(109)**, myxin (cuprimycin) **(110)**, oxychlororaphine **(111)**, phenazine-1-carboxylic acid **(112)**, phenozostatin D **(113)**, pyocyanin **(114)**, pyorubin (aeruginosinA+B) **(115)**, and pyoverdin **(116)**; quinones: arpink red **(117)**, averythrin **(118)**, austrocortinin **(119)**, bostrycoidin **(120)**, catenarin **(121)**, cercosporin **(122)**, chlorobiumquinone **(123)**, 7-chloroemodin **(124)**, chrysophanol **(125)**, citreorosein **(126)**, cynodontin **(127)**, dermocybin **(128)**, dermoglaucin **(129)**, dermorubin **(130)**, draconin **(131)**, elsinochromes **(132)**, emodin **(133)**, erythroglaucin **(134)**, fallacinal **(135)**, flaviolin **(136)**, flavomannin **(137)**, fusarubin **(138)**, helminthosporin **(139)**, javanicin **(140)**, juglone **(141)**, Karuquinone A **(142)**, menaquinone-7 **(143)**, naphthoquinone **(144)**, nectriachrysone **(145)**, pachybasin **(146)**, parietinic acid **(147)**, phomaligin A **(148)**, phomarin **(149)**, physcion **(150)**, piloquinone **(151)**, questin **(152)**, rubellin D **(153)**, rubrocristin **(154)**, skyrin **(155)**, spinulosin **(156)**, teloschistin **(157)**, tritisporin **(158)**, and Xylindein **(159)**; monascus pigments: ankaflavin **(84)**, lovastatin **(160)**, monascins **(161)**, monascoflavin **(85)**, monascorubramine **(162)**, monascorubrin **(163)**, rubropunctamine **(164)**, and rubropunctatine **(165)**; other compounds: acetylazulene **(166)**, actinorhodin **(167)**, akashin **(168)**, albidin **(169)**, alterperylenol **(170)**, amitenone **(171)**, ammosamide A **(172)**, ammosamide B **(173)**, atranorin **(174)**, atromentin **(175)**, aulosirazole **(176)**, aurantricholide B **(177)**, aurasperone A **(178)**, azaphilone **(179)**, azulene **(180)**, boviquinone 3 **(181)**, bromoalterochromides **(182)**, calycin **(183)**, candidin **(184)**, chloronatronochrome **(185)**, chrysogenin **(186)**, citrinin **(187)**, cochliodinol **(188)**, cordycepin **(189)**, cordycepoid A **(190)**, diastaphenazine **(191)**, dihydroalterperylenol **(192)**, dihydroxyazulene **(193)**, dolastatin **(194)**, epicocconone **(195)**, floccosin **(196)**, fluorescein **(197)**, fonsecin **(198)**, glauconic acid **(199)**, glaukothalin **(200)**, gomphidic acid **(201)**, granadaene **(202)**, grevilline A **(203)**, gyrophoric acid **(204)**, haematopodin **(205)**, hyaluromycin **(206)**, hypericin **(207)**, iridosporin **(208)**, lactaroviolin **(209)**, laetiporic acid A **(210)**, lilacinone **(211)**, luteosporin **(212)**, magnesidin **(213)**, marineosin A **(214)**, marinone **(215)**, melanocrocin **(216)**, mitorubrin **(217)**, mycenaaurin A **(218)**, N-carboxamidostaurosporine **(219)**, natronochrome **(220)**, nicotine **(221)**, nostocine A **(222)**, panosialin **(223)**, peridinin **(224)**, pestalone **(225)**, Phlegmacin A **(226)**, phenoxazine **(227)**, porphyrin **(228)**, pulvinic acid **(229)**, purpuride **(230)**, pyrandione **(231)**, pyrrocidine A **(232)**, rhizocarpic acid **(233)**, roseophilin **(234)**, rubrolone **(235)**, rubrosporin **(236)**, rubrosulphin **(237)**, rumbrin **(238)**, sanguinone A **(239)**, scytonemin **(240)**, siroheme **(241)**, sorbicillin **(242)**, stearoyldeterrol **(243)**, sterigmatocystin **(244)**, streptochlorin **(245)**, tambjamine **(246)**, tetrabromopyrrole **(247)**, thermorubin **(248)**, tryptanthrin **(249)**, variegatorubin **(250)**, viomellein **(251)**, viopurpurin **(252)**, vulpinic acid **(253)**, xanthomegnin **(254)**, bacterial luciferin **(255)**, dinoflagellate luciferin **(256)**, blue fluorescent protein-lumazine (BFP) **(257)** and yellow fluorescent protein (YFP) chromophore **(258)**; and chlorophylls: bacteriochlorophylls **(259),** chlorophylls **(260)** and divinyl-chlorophylls **(261)** occurring in different microorganisms are illustrated (Figure 1; Table S1). Here, we also categorize the microbial pigments into two categories: (1) Fluorescent pigments (phycoerythrins, fluorescein, epicocconone, BFP, and YFP) and (2) nonfluorescent pigments (rest of the pigmented compounds as detailed above).

## 3. Brief Historical Note on Microbial Pigments

In 1879, a natural yellow pigment called “lactoflavin” was obtained from milk. In 1932, a yellow dye from aqueous yeast extracts was fractioned by Warburg and Christian. Afterwards, Karrer and Kuhn elucidated the yellow pigment called riboflavin, and both of them were endowed with the Nobel Prizes in chemistry for Karrer and Kuhn 1937 and 1938, respectively [22]. In the early 1970s, the purple pigment bacteriorhodopsin of *Halobacterium* was discovered [23]. Several pigmented non-photosynthetic bacteria and fungi were isolated during 1934 and 1976 by Ingraham and Baumann (both of them had conducted a systematic survey of carotenoid-producing non-photosynthetic bacteria in the 1930s) and Valadon respectively [24]. Monascus pigments are the well0 known natural food colorants known around the world since 1884 [25]. In Asia, for more than 10 decades, monascus red pigments appear to be used as food colorants to red pot-roast lamb and red rice koji [21]. So far, a total of 65 different monascus pigmented compounds have been reported, and some of which possessing antimicrobial, anticancer, and anti-obesity activities were recently well reviewed [26]. In 1934, ZoBell and Feltham found that 69.4% of bacterial colonies grown on agar medium inoculated with seawater and marine sediment were chromogenic. An infallible literature summary carried out by ZoBell in 1946 shows that many of marine bacterial species which spoil fish appeared to be pigmented [27]. Zeaxanthin producing *Flavobacterium* was isolated during the mid-1960s by scientists at Hoffmann-La Roche [21]. In 1964, thermorubin, a red pigment, was first isolated from a mildly thermophilic soil actinomycetes *Thermoactinomyces antibioticus* [28].

## 4. Host Pigmented Compounds Said to Be of Microbial Origin

Dolastatin, a well-known antitumor compound isolated from different marine invertebrate species like sea hares and molluscs, has recently been found to actually have originated from their symbiotically associated marine cyanobacteria [13]. Pigments produced by some marine plants, invertebrates, and vertebrates such as seagrass, sponges, corals, molluscs, and tunicates are indeed produced by their epibiotic bacteria [29]. Some of the compounds such as Tambajamine, a yellow pigment molecule isolated from sponges and bryozoans, are believed to originate from endobiotic or epibiotic *Pseudoalteromonas* [30]. Tambjamines isolated from bryozoans (*Bugula dentata* and *Sessibugula translucens*), nudibranchs, and ascidians (*Atapozoa* sp.) have been found to be produce by *Streptomyces* sp., *Pseudoalteromonas tunicate*, and *Serratia marcescens* [31,32].

## 5. Ecology and Habitats of Pigmented Microorganisms

A plethora of research articles have reported the isolation of pigmented microorganisms like bacteria, fungi, and yeast from terrestrial as well as marine milieus. They are distributed in different geographical conditions, from polar regions to tropical environments and from aerial to deep-sea regions. It is believed that microorganisms from different geographical regions are known to tolerate harsh conditions by producing pigments. Some of the pigmented microbes such as bacteria (e.g., *Stenotrophomonas*) and yeast (e.g., *Rhodotorula*) from terrestrial environment are found to enter coastal environments through discharges from hospitals and domestic sewages, thereby adapting to marine environment. Literature survey indicates that pigmented bacteria could be divided into two categories of true marine pigmented bacteria—primarily of marine origin and adaptive pigmented bacteria—originated from terrestrial ecosystem and survive and proliferate in coastal environment (Figure 1). Irrespective of the common occurrence of PB in terrestrial environment, marine pigmented microbes are gaining more attention due to their varied bioactive pigment compounds.

**Figure 2:**
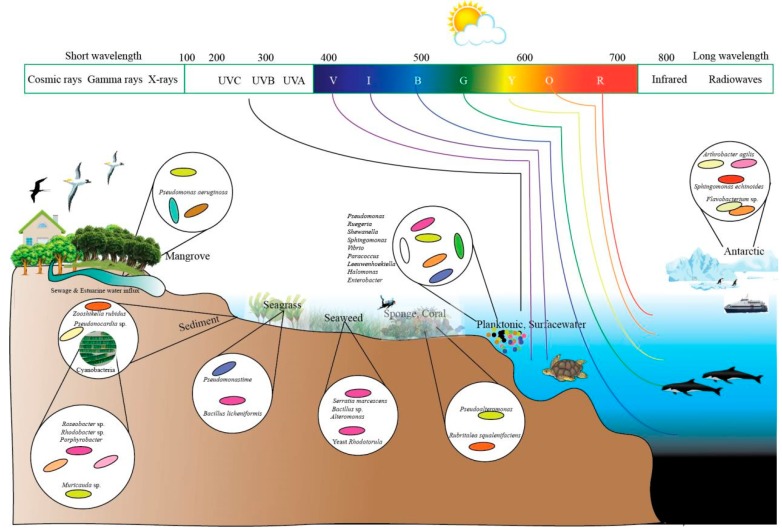
Distribution of marine pigmented microorganisms in different niches.

Recent studies have been diverted to investigate marine microbial pigments as novel chromogenic compounds for biotechnological and industrial application. The occurrence of PB in a marine environment is found to vary according to geographical and nutritional conditions. Apparently, the diversity of pigmented heterotrophic bacteria (PHB) is less in abundance when compared to the enormous diversity of marine heterotrophic bacteria (MHB). Green and blue pigments are rare colors produced by microorganisms. The colony forming units (CFU) of PHB may vary depending on sampling site, seasonal variation, and availability of nutrients. Occurrence of high frequency of pigmented bacteria is noticed in air–water interfaces [33], glaciers [34], ice cores [35], bacterioneuston (sea surface microlayer) and underlying waters [36], salt lakes [37], deepsea hydrothermal vents [38], and abyssal hot springs (e.g., *Thermus*). Recently, various pigmented bacterial communities have been isolated from lava caves [39]. *P. aeruginosa*, a pigmented bacterium, has been reported to isolate from the wounds skin of humans and animals. These PBs are reported to be isolated from different marine niches such as seawater, marine sediment, seagrass, sponge, mussel, sea cucumber [40], algal mats, corals, freshwater, athalassohaline lagoon, marine solar saltern, microbial mats in Antarctic lakes, oil contaminated soil, nonsaline alkaline groundwater, and sea ice (e.g., *Algoriphagus*) [41] (Figure 2). 

Several microbes are noticed exhibiting polyextremophilic characteristics according to their environments, for instance, xerophilic (*Penicillium purpurogenum*) [42], dimorphic (*Metschnikowia laotica*), pleomorphic (*Arthrobacter*), extreme halophilic (*Salinibacter*), thermophilic (*Thermus*), psychrophilic (*Kocuria polaris*), acidophilic (*Acidobacterium*), alkaliphilic (*Microbacterium arborescens*), radioresistance (*Deinococcus grandis*), polyextremophile (*Halorubrum*), barophilic or piezophilic (*Halomonas salaria*), and color mimic (*Cellulophaga lytica*). Various species of microalgae distributed in different environments are also reviewed by different authors [43,44,45,46,47,48]. Factors driving the limited dispersal of these microorganisms in their respective environment are poorly understood.

## 6. Uses of Microbial Pigments

### 6.1. Biological Significance

Empirically, it is well-understood that most of the microbial pigments found as variety of hues are known to act as defensive systems against UV irradiation, thereby protecting and increasing their survivability [36] and adapting to the surrounding environmental conditions [49] compared to nonpigmented microbes. Carotenoid pigmentation in Antarctic heterotrophic bacteria withstand environmental stresses by adaptation to cold environments [50]. Symbiotic or epibiotic association of some bioactive pigmented bacteria with their host organisms indicates their defensive role in protecting their host from other pathogenic microorganisms and predatory fouling organisms [51]. It was investigated that C50-carotenoids produced by the extremophile microorganisms *Halococcus morrhuae*, *Halobacterium salinarium*, and *Thermus filiformis* are known to be important for their survival as these pigments stabilize their cell membrane and also act as antioxidant agents [52]. Toxic oxygen molecules such as reactive nitrogen species, reactive oxygen species, and other nonbiological radicals formed in the cells are efficiently reduced by carotenoid [52,53]. A violet compound, violacein produced by *Chromobacterium violaceum*, has been reported to protect lipid membranes such as rat liver microsomes and egg and soy-bean phosphathidylcholine liposomes against peroxidation induced by reactive hydroxyl radicals [54]. Bacterial phenazines are known to regulate cellular gene expressions that trigger the survival and biofilm formation by the bacteria [55]. It is also hypothesized that *Thermus* strains in natural thermal areas exposed to sunlight are protected by yellow pigmentation [56].

Unambiguously, it was evident that prodigiosin producing *Vibrio* strains have survived under UV exposure (324 J/m^2^) around 1000-fold more successfully as compared to non-pigment-producing vibrios [57]. Bacterial melanins are known to act as cellular protectors by neutralizing diverse toxic chemical compounds like drugs and antibiotics [58] and are one of the survival fitness factors to tolerate stressful physiological conditions like hyperosmotic stress, starvation, and high temperature as observed with *Vibrio cholerae* [59]. Self-survival defensive mechanism in *Janthinobacterium lividum* and *Chromobacterium violaceum* has been related to violacein pigments which have caused cell death to common bacterivorous nanoflagellates *Ochromonas* sp., *Spumella* sp., and *Bodo saltans* when fed on them [60]. The beneficial roles of bacterial pigments are also perceived as protection from phagocytosis. Similarly, indigoidine, a blue quinine compound produced by some *Roseobacter* strains, annihilates other potentially out competing bacteria; therefore, *Roseobacter* survives in the environment [2]. Pyoverdin produced by *P. fluorescens* was presumed to have a role in facilitating iron transport as well [61]. 

Investigations also revealed that marine pigmented bacteria are more resistant to heavy metals and antibiotics compared to nonpigmented bacteria [62]. Melanins in *Rhizobium* species were found to be involved in the detoxification of polyphenolic compounds accumulated in senescing nodules [63]. Fungal melanins are known to protect fungi from UV and solar radiation (photodestructive impact) and to also inhibit cell-wall-degrading enzymes produced by other microorganisms. Anthraquinones produced by endophytic fungi are found to protect the host plant from insects or other microorganisms [64]. Tambjamines produced by diverse organisms are referred to as natural defensive compounds against predators [31]. Photosynthetic bacteria possess bacteriochlorophylls, bacteriorhodopsins, and proteorhodopsins, which are similar to chlorophylls. Bacteriorhodopsins are light harvesting membrane proteins that enable bacteria to obtain energy when a low amount of organic matter occurs, while halorhodopsin serves as an inward-directed chloride pump and proteorhodopsin serves as a proton pump [65]. In brief, microbial pigments are known to play important roles in different ways including antioxidant activities [54], photosynthesis, cell signaling communication, radiation protection [66], UV absorption [67], antibiotic activities [68], virulence [69], and membrane stabilization [70]. Pigment trait is also used as biological markers for taxonomic identification and the discrimination of different microbes [70]. Interestingly, *Claviceps purpurea*, an aposamatic fungus, displays a wide range of colors, i.e., yellow, orange, red, and black as warning sign to the predators [71].

### 6.2. Industrial Significance

Microbial pigments, especially bacterial pigments are getting more attention due to their wide application in textiles dyeing, cosmetics, food colorants, painting, pharmaceuticals, plastics, etc., and it was assumed that bacterial pigments are to dominate the pigment industries and organic market in near future. Considerably, consumer demand on food grade of important natural microbial pigments such as β-carotene, riboflavin and phycocyanin is increasing in niche markets [72]. These pigments in the foods serve as preservatives and antioxidants [73]. Synthetic colorants are also employed but are found to cause sickness, so natural pigments are highly preferred over the use of synthetic pigments. Carotenoids obtained from *Haematococcus pluvialis* and *Phaffia rhodozyma* are being utilized in pharmaceutical, food additives for animals and fish, and aquaculture industries [65]. Astaxanthins from *H. pluvialis* are also used in aquaculture feeds by aquaculture industries and appear to play a role in memory improvement and antiaging [74]. Xanthan gum, a well-known exopolysaccharide produced by *Xanthomonas campestris*, is being used as a food additive. Phycocyanin from cyanobacteria is rich in proteins and hence used as dietary supplement; Riboflavin from *Bacillus subtilis* is used in foods, vitamin enriched milk products, and energy drinks; flexirubin produced by *Chryseobacterium* and *Flavobacterium* are used in the treatment of chronic skin disease, eczemea, gastric ulcers, etc.; and bacterial pigments as an indicators of oils spill and as biosensors and markers of water, soil, and air pollution are also known [72]. Several biomedical applications of microbial pigments are detailed in the Supplementary Materials (Table S1).

Cyanobacteria possess chlorophyll “a” and also other pigments like carotenoids, the blue phycobiliproteins, phycocyanin, and allophycocyanin which are potential antioxidants. Strains of *Anabaena*, *Nostoc*, and *Spirulina* are consumed as human food in many countries, and *Arthrospira platensisis* is marketed in the form of flakes, powder, tablets, and capsules [75]. In Japan, cyanobacterial pigments such as phycocyanin and phycoerythrin are being used for coloring candy, ice cream, yogurt, dairy products, and soft drinks. Phycocyanin obtained from *Spirulina* is being used in preparing bio-lipsticks (e.g., red pigment from *Haematococcus*), bio-eyeliners, bio-eye shadows, creams, and soaps. Phycocyanin and phycoerythrins of *Spirulina* are also being used in fluorescent microscopy, in immunoassays, and as phycofluoures for DNA probes [76,77]. Application of carotenoids as coloring agents for cooked sausages (e.g., bologna and frankfurters), soft drinks (e.g., cola), and baked goods (e.g., Livarot cheeses) have been investigated [78]. Azaphilone pigments and Arpink red™ (Natu-ral Red™) obtained from *Monascus* species and *Penicillium oxalicum*, respectively, have wide applications as red food colorants [21]. Microalgal pigments are also widely being used in aquaculture application, cosmetics, creams, jellies, etc. [79].

A survey by the Infectious Disease Society of America (IDSA) has raised alarm on the urgent threat of antibiotic resistant microbial pathogens, and the WHO has undertaken a project to develop a list of global R&D priorities with respect to drug-resistant infectious microorganisms. Succinctly, here, the therapeutic uses of different microbial pigmented compounds are described below, which may be highly appreciable to use against various diseases including drug resistant microorganisms and cancer cells.

#### 6.2.1. Antibacterial Activity

Prodiginine compounds like prodigiosin, undecylprodigiosin, cycloprodigiosin, heptylprodigiosin, nonylprodigiosin, cyclononylprodigiosin, and cyclomethyl-decylprodigiosins are well-known to exhibit various biological properties including antibacterial activities against different gram-negative and gram-positive bacterial members [80]. Tambjamines and other members of this class compounds are produced by marine bacteria like the well-known *Pseudoalteromonas tunicata* and possess a wide range of antibacterial activities [49]. Violacein extracted from *Janthinobacterium lividum* and *Chromobacterium violaceum* exhibited a wide range of antibacterial activity against gram-positive and gram-negative bacteria [81]. Tetrabromopyrrole, the yellow pigment extracted from *Chromobacterium*, a seawater isolate, was known to inhibit different human pathogens as well as marine bacteria including autoinhibition of the producing bacteria [82]. Several other phenazine compounds [83], quinones [63], and anthroquinones biosynthesized by different bacterial and fungal species also showed a broad range of antibacterial activities [64].

#### 6.2.2. Antifungal Activity

Prodiginines such as prodigiosin, undecylprodigiosin, and cycloprodigiosin compounds have contributed as fungicidal agents against several fungi such as *Coccidioides*, *Candida*, *Didymella*, *Aspergillus*, *Penicillium*, *Saccharomyces*, *Cryptococcus*, *Histoplasma*, *Trichophyton*, and *Verticillium* [80]. Tambjamines are yellow pigments and are believed to be originated from bacterial species such as *Pseudoaltermonas tunicata* and to possess antifungal properties [30]. Fungicidal activity of violacein isolated from *Janthinobacterium lividum* has also been reported against white root rot causing phytopathogenic fungi *Rosellinia necatrix* [84]. Some anthraquinone compounds synthesized by *Trichoderma harzianum*, *Curvularia lunata* [64] and phenazine compounds synthesized by *Pseudomonas* and *Streptomyces* species are also demonstrated to have antifungal activities against various fungal species [83].

#### 6.2.3. Antiviral Activity

Phenazine compounds synthesized by *Pseudomonas* and *Streptomyces* species have been reported to show promising antiviral activities [83]. Violacein demonstrated a significant level of antiviral activities against herpes simplex virus, poliovirus, and simian rotavirus SA II [81]. Quinone compounds such as benzoquinones, naphthoquinones, and anthraquinones are well-known to demonstrate antiviral properties [64,85]. 

#### 6.2.4. Antimetastatic Activity

In vitro and in vivo investigations on the antimetastatic activity of prodigiosin revealed the inhibition of metastatic nodules of human highly metastatic lung carcinoma 95-D cells and the highly metastatic substrain B16BL6 of mouse melanoma B16 cells. Results also showed the elevated survival rate of mice, indicating the potentiality of prodigiosin as an antimetastatic compound that is to be focused for further research [86].

#### 6.2.5. Immunosuppressive Activity

Intriguingly, cycloprodigiosin hydrochloride, a red pigment obtained from *Pseudoalteromonas denitrificans*, is stable under several physicochemical conditions and demonstrated immunosuppressive activity by inhibiting the proliferation of T cells and PMA (Phorbol 12-myristate 13-acetate) stimulated Jurkat cells [87]. At nontoxic concentrations, prodigiosin inhibited the T-cell mediated immune functions such as concanavalin-A induced proliferation, mixed lymphocyte response, local graft vs. host reaction, and T-dependent antibody response [88]. Undecylprodigiosin demonstrated the inhibition of purified peripheral human T and B lymphocytes with an IC_50_ of 3 to 8 ng/mL and elicited the inhibition of retinoblastoma protein phosphorylation by inhibiting cyclin-dependent kinase-2 and cyclin-dependent kinase-4 in human lymphocytes [89]. Tambjamine alakaloids and its various other related members are found to possess immunosuppressive activities [49].

#### 6.2.6. Antitumor Activity

A well-detailed recent review by Soliev and Enomoto [13] corroborated that several pigmented compounds belong to structural classes of polyketide, pyrroloiminoquinone, indolocarbazole, butenolide, phenoxazinone, alkaloid, phycobiliprotein, terpenoid dihydroquinones, phenazine, peptides, indole, and pyrrole alkaloid known to be produced by marine *Pseudoalteromonas*, marine Actinomycetes, marine cyanobacterial species, and other bacterial species with potential antitumor activities. Numerous marine- and terrestrial-derived fungal species produce pigmented anthroquinone compounds, which have potential in inhibiting tumor cells [90]. 

#### 6.2.7. Anti-Alzhelmeric Activity

Phycobilioproteins originating from red algae and cyanobacterial species are shown to display anti-alzhelmeric activity [77].

#### 6.2.8. Antiatherosclerosis Activity

The repression of lipid peroxidation and atherosclerotic plaque by *Monascus*-fermented red mold dioscorea (RMD) including a higher monacolin K level and a dioscorea substrate have contributed to potent anti-atherosclerotic effects with 48 mg/kg/day [91].

#### 6.2.9. Antihypertensive Activity

Significantly, the oral administration of monascus-fermented dioscorea at a low-dose (150 mg/kg) in spontaneously hypertensive rats (SHRs) has revealed decreased systolic and diastolic blood pressures [92]. The antihypertensive activity had earlier been reported from the microalgae *Dunaliella tertiolecta* [93].

#### 6.2.10. Anticancer Activity or Antineoplastic Activity

Prodigiosin pigments produced by *Serratia marcescens* have induced apoptosis in haematopoietic cancer cell lines and human colon cancer cells activities [94]. Quinones are yellow to red compounds that demonstrated significant anticancer activities [63]. Significantly, violacein extracted from *C. violaceum* showed cytotoxic effects and apoptosis of different cancer cells including colorectal cancer, uveal melanoma, leukemia, and lymphoma cells in culture [69]. A yellow pigment producing *Pseudoalteromonas piscicida* strain NJ6-3-1 isolate obtained from sponge *Hymeniacidon perleve* possesses cytotoxic activity on cancer cells HeLa or BGC-823 cell lines, with IC_50_ values of 150 ± 4.6 and 192 ± 3.5 µg/mL, respectively [95]. Bacterial phenazine compounds also appeared to be potential anticancer agents [96]. Monascus pigments such as monascin, ankaflavin, monaphilone A and monaphilone B, monascuspiloin, monascorubrin, rubropunctatin, and monascorubramine exhibited significant cytotoxic activities against various cancer cell lines [26]. β-carotene synthesized from microalgal species have been found to be a potential anticancer agent in human and animal model studies [79]. Phycobilioproteins produced by different cyanobacterial species and red algae are also known to be anticancer agents [77].

#### 6.2.11. Anti-Tuberculosis Activity

Violacein and flexirubin pigments isolated from Antarctic bacteria *Janthinobacterium* sp. Ant5-2 and *Flavobacterium* sp. Ant342 demonstrated the growth inhibition of *Mycobacterium tuberculosis* with minimum inhibitory concentrations (MICs) of 34.4 and 10.8 µg/mL for virulent *M. tuberculosis*, respectively [97]. 

#### 6.2.12. Antifouling Activity

Pigmented *Pseudoalteromonas* bacterial species isolated from marine plants and animals appeared to be effective inhibitors against common fouling organisms such as invertebrate larvae of *Hydroides elegans* and *Balanus amphitrite*, algal spores of *Ulva lactuca* and *Polysiphonia* sp., diatoms, bacteria, and fungi [98]. The same inhibitory activity was recently reported from prodigiosin producing *S. marcescens* CMST07 that inhibited marine fouling bacteria like *Alteromonas* sp. and *Gallionella* sp. with a minimum inhibitory concentration (MIC) and a minimum bactericidal concentration (MBC) of 6.75 and 12.5 µg/mL, respectively [99]. Also, Prodigiosin was found to inhibit cyanobacterial adhesion on glass surfaces [99]. Investigations have also reported the correlation between pigmentation and antifouling activities [51,98].

#### 6.2.13. Anti-Algicidal Activity

Serendipitously, purified prodigiosin extracted from *Hahella chejuensis*, a marine bacterium, showed complete inhibition of algicidal activity against a major red-tide dinoflagellate *Cochlodinium polykrikoides* at a 10^−1^ mg/L concentration [100] or at low concentrations as ~1 ppb [101]. Xylindein, a blue-green compound from a fungal species *Chlorociboria aeruginosa*, was found to control the *Chlorella* growth [102]. 

#### 6.2.14. Anti-Insecticidal Activity

Naphthoquinone pigments such as fusarubin, javanicin, and related compounds are reported to display insecticidal activities [63,64].

#### 6.2.15. Anti-Herbicidal Activity

Anthraquinones from several fungal species such as *Phoma exigua* var. *exigua, Phoma foveata, P. glomerata, P. herbarum, P. macdonaldii, P. macrostoma, P. multirostrata, P. proboscis, P. sorghina*, and *P. tracheiphila* possess herbicidal activities [103]. Quereshi et al. (2011) [104] isolated a pigment compound—anhydropseudophlegmacin-9,10-quinone-3′-amino-8′-*O*-methyl ether—from *Phoma herbarum* FGCC#54 that showed potential herbicidal activity against prominent weeds *Hyptis suaveolens*, *Lantana camara*, *Parthenium hysterophorus*, and *Sida acuta*. 

#### 6.2.16. Antiparasitic Activity

The violacein pigment compound obtained from *Chromobacterium violaceum* has exhibited in vitro antiparasitic activity as trypanocide activity by the growth inhibition of *Trypanosoma cruzi* [105]. Lopes et al. (2009) [106] reported the inhibition of chloroquine-sensitive and -resistant strains of *Plasmodium falciparum* by violacein with an IC_50_ value of 0.85 ± 0.11 µM. 

#### 6.2.17. Antiprotozoal Activity

Violacein extracted from freshwater isolates of *Janthinobacterium lividum* and *Chromobacterium violaceum* is reported to have protozoan-killing efficiency against cultures of nanoflagellates *Spumella* sp. and *Ochromonas* sp. At higher concentrations of >10 µM, it resulted in the complete reduction of the cells, indicating their defensive role in avoiding being ingested by these nanoflagellates [60]. Antiprotozoal activities by red pigment prodigiosin have also been reported from *Serratia* [107].

#### 6.2.18. Antileishmanial Activity

Reduction in viability/growth inhibition of *Leishmania amazonensis* with violacein compound was observed at the concentration of EC_50_/24 h value of 4.3 ± 1.15 µmol/L [108]. Prodigiosin producing *S. marcescens* variant SM 365 has evidently contributed to the lysis of *Leishmania chagasi* [109].

#### 6.2.19. Antiulcerogenic Activity 

Violacein, a purple violet pigment, has demonstrated increased inhibition of gastric damage (ulcer formation) in the presence of β-cyclodextrin (βCD) inclusion complexation at 1:1 and 1:2 molar ratios compared to that of violacein [110]. Flexirubin pigments from *Chryseobacterium* and *Flavobacterium* are used in treatment for chronic skin disease, eczemea, gastric ulcers, etc. [8,72].

#### 6.2.20. Antilipoperoxidant Activity

Violacein and βCD inclusion complexation at 1:2 ratio exhibited four-fold potent antilipoperoxidant activity compared to violacein in rat liver cells by a 40% inhibition of malonaldehyde (MAD) with an IC_50_ of 125 and 505 µM [110].

#### 6.2.21. Anti-HIV Activity

Compounds extracted from pigmented *Phoma* species have demonstrated inhibition of HIV virus integrase [103]. In vitro investigations were also initiated to evaluate the effect of violacein on AIDS-related lumphoma [111].

#### 6.2.22. Anti-Malarial Activity

Violacein, a violet pigment extracted from *Chromobacterium violaceum* is known to exhibit more effective antimalarial activity against *Plasmodium falciparum* strains in vitro [106]. Prodiginines such as cycloprodigiosin, prodigiosin, undecylprodigiosin, heptyl prodigiosin, and metacycloprodigiosin have demonstrated the antimalarial activity against *P. falciparum* [80,112,113]. Liu et al. (1993) [114] reported the mosquitocidal activity of melanin produced by *Bacillus thuringiensis* subsp. *israelensis*.

#### 6.2.23. Antitrypanosomal Activity

Recently, Genes et al. [115] reported that prodigiosin extracted from *S. marcescens* have apparently appeared to cause cell death of *Trypanosoma cruzi* by disrupting the mitochondrial function and by interfering with the oxidative phosphorylation processes. In vitro experiments also demonstrated that merely prodigiosin producing *S. marcescens* has resulted in cell lysis of trypanosomatid protozoan parasites *T. cruzi* [81,116].

#### 6.2.24. Antinematodal Activity

Phenazine compounds extracted from *Pseudomonas fluorescens* are found to suppress egg hatching and to enhance the juvenile mortality of root knot nematode, *Meloidogyne incognita*, in vitro [117].

#### 6.2.25. Anti-Inflammatory Activity

Investigations on red mold dioscorea (RMD) have demonstrated the anti-inflammatory effects in STZ-induced diabetic rats by reducing inflammatory cytokine TNF-*α* levels and enhancing IL-2 cytokine expression [118]. Monascin, a yellow metabolite of monascus, displayed anti-inflammatory activity by inhibiting inflammatory the signal pathways of PKC (protein kinase C) and JNK (c-Jun N-terminal kinase) phosphorylation in a C2C12 cell model [119]. 

#### 6.2.26. Antihypertriglyceridemia Activity

A study corroborated that increased levels of high-density lipoprotein and decreased levels of triglycerides (TG) and glycosylated hemoglobin (HbA1c) in DM (diabetes mellitus) + 1X RMD and DM + 5X RMD supplemented rats displayed antihypertriglyceridemia activity [118].

#### 6.2.27. Anti-Atherosclerotic Activity

Ankaflavin and monascin are proven to prevent the accumulation of fatty liver and lipid plaque and enhanced high-density lipoprotein cholesterol, respectively, in heart aorta of hamsters [120]. It was concluded that ankaflavin also acts as a potential hypolipidemic agent [120]. Astaxanthin from different microbial sources has been reported to exert preventive actions against atherosclerotic cardiovascular disease by the enhancement of oxidative stress, inflammation, lipid metabolism, and glucose metabolism [121].

#### 6.2.28. Antioxidant Activity

Regardless of common carotenoids like lutein, β-carotene, astaxanthin, etc., the antioxidnat activity of rare C_50_ carotenoids such as sarcinaxanthin, sarcinaxanthin monoglucoside, and sarcinaxanthin diglucoside with IC_50_ values of 57, 54, and 74 μM, respectively, were reported from a halophilic bacterium *Micrococcus yunnanensis* strain AOY-1 isolated from hard coral [122]. Violacein is a strong antioxidant compound that can protect lipid membranes from peroxidation caused by hydroxyl radicals [49,54]. Monascus pigments are reported to act as effective antioxidants [26]. Carotenoids with both large numbers of conjugated double bounds and of hydroxyl groups appeared to have strong antioxidant activity. Mandelli et al. [52] reported the antioxidant activity demonstrated by extremophile microorganisms *Halococcus morrhuae* (IC_50_ = 0.85 µg·mL^−1^), *Halobacterium salinarium* (IC_50_ = 0.84 µg·mL^−1^), and *Thermus filiformis* (IC_50_ = 2.41 µg·mL^−1^). A structurally unusual phenolic carotenoid, 3,3′-dihydroxyisorenieratene isolated from the bacterium *Streptomyces mediolani* [123], phycobiloproteins from cyanobacterial species, and some algal species have demonstrated powerful antioxidant activity [77]. Cyanobacterial pigments such as β-carotene, lycopene, lutein C-phycocyanin, and phycobilioproteins are known to demonstrate antioxidant properties [77].

#### 6.2.29. Anti-Proliferation Activity

Undecylprodigiosin also acts as an anti-proliferative agent against human T and B lymphocytes with an IC_50_ value of 3 to 8 ng/mL [89]. Astaxanthin from the yeast *Phaffia rhodozyma* demonstrated the antiproliferative activity on MCF-7 and MDA-MB231cell lines [124]. Tambjamines [49] and beta-carotene [125] are also reported to possess anti-proliferation activities.

#### 6.2.30. Anti-Aging Activity

Natural astaxanthin pigments appeared to be potential anti-aging supplements [74]. Also, water-soluble phycobilioproteins biosynthesized from cyanobacteria and red algae are found to show potential anti-aging activities [77]. 

#### 6.2.31. Anti-Obesity Activity

l-Tryptophan and l-leucine ethyl ester derivatives of the monascus pigments are GRAS (generally recognized as safe) compounds showing an anti-obesity effect on mice by inhibiting cholesterol and triglyceride contents [126]. Monascin and ankaflavin have reduced the preadipocyte proliferation of 3T3-L1 cells at a 8-μg/mL concentration; decreased the triglyceride accumulation; and suppressed the expression of adipocyte specific transcription factors, C/EBP*β*, C/EBP*δ*, PPAR*γ,* and C/EBP*α* [120,127]. TEA (2-(p-toyly) ethylamine), an amine derivative of monascus pigment, reduced the total cholesterol (24%) and LDL (low-density lipoprotein) cholesterol (38%) content in C57BL/6 mice serum [128]. The red mold dioscorea (RMD) cultured with deep ocean water (DOW-RMD) with increased levels of monascin and ankaflavin have displayed anti-obesity effects by inhibiting PPARγ and C/EBPα expression in differentiation and lipoprotein lipase activity [129].

#### 6.2.32. Anti-Diabetic Activity

Monascus fermented red mold dioscorea appeared to delay diabetes by showing antioxidant effects, protection of pancreatic β-cells, and control of hyperglycemia by decreasing blood glucose and serum-free fatty acid levels in Streptozotocin-induced diabetic rats [118]. Similarly, monascus fermented durian seed (MFDS) ethanol extracts have exhibited potentiality towards diabetes mellitus by α-glucosidase inhibitory activity with an IC_50_ of 70.7 µg/mL [130]. Monascins are found to prevent PPAR-γ phosphorylation by phospho c-Jun N-terminal kinase (p-JNK) to exhibit anti-diabetic activity [119].

#### 6.2.33. Antiadipogenic Activity

Monascin and ankaflavin also promote delipidation of mature adipocytes by glycerol release by 113.2% and 278.3% and reduce the downregulation activity of HR-LPL (heparin-releasable lipoprotein lipase) by 45.3% and 58.1%, respectively [127]. High fat diet (HFD) supplemented with the amine derivatives of monascus pigments, 4- phenylbutylamine (PBA) (2.5 µM), and 2-(p-toyly) ethylamine (TEA) (12.5µM) have demonstrated an inhibitory activity against adipogenic differentiation in 3T3-L1cells [128]. Metals present in deep ocean water are found to cause synergistic effects on the production of monascin and ankaflavin, and DOW with RMD shows a significant anti-adipogenesis effect [131].

#### 6.2.34. Ichthyodeterrent Activity

New tambjamine compounds isolated from ascidian *Atapozoa* sp. [132], and bryozoan *Bugula dentata* [31] appeared to originate from their associated symbiotic bacteria *S. marcescens* and possess Ichthyodeterrent activities. 

#### 6.2.35. Conjugated Antibodies

Phycoerythrins are widely used in fluorescent probes and have been commercialized as conjugated antibodies [133].

#### 6.2.36. Cytotoxic Activity

A marine bacterial strain *Pseudoalteromonas maricaloris* KMM 636^T^ isolated from sponge *Fascaplysinopsis reticulata* was found to produce two brominated yellow pigments bromoalterochromide A and A′. These compounds displayed a cytotoxic effect on developing eggs of the sea urchin *Strongylocentrotus intermedius* [134]. Grossart reported the cytotocic effect of deep blue pigment glaukothalin extracted from *Rheinheimera* strains (isolated from diatom aggregates and organic particles) against *Artemia salina* (*c* = 0.1 mg/mL, mortality = 100%) [135]. 

#### 6.2.37. Inducing Activity as Larval Metamorphosis

Tetrabromopyrrole isolated from four *Pseudoalteromonas* bacterial strains have induced metamorphosis of acroporid coral *Acropora millepora* larvae, i.e., planulae transformation into fully developed polyps within 6 h, indicating that the settlement of these larvae on crustose coralline algae is mediated through epibiotic microbes [136]. Similarly, the enhanced production of eggs and juveniles was observed upon the addition of lutein and zeaxanthin to the adult diet of sea urchin *Lytechinus variegatus* [137].

#### 6.2.38. Miscellaneous Activities

Lutein and zeaxanthin are being used in nutraceutical and as dietary supplements to prevent cardiovascular diseases, cancers, cognitive function, and age-related macular degeneration (AMD) [21]. Evidently, cell free culture filtrates (with presence of 1.09 to 9.89 µg·mL^−1^ of cytokinins) of pink pigmented *Methylobacterium* strains isolated from the phyllosphere of different crop plants such as sugarcane, pigeonpea, mustard, potato, and radish have enhanced the seed germination of wheat *Triticum aestivum* [138]. Monascin and rubropunctatin pigments from *Monascus purpureus* have displayed teratogenic effects on chicken embryos [139]. Phycobilioproteins extracted from cyanobacterial species are being used as fluorescence probes as protein markers for gel electrophoresis [77]. Evidently phycoerythrin-feeding appeared to increase the mean survival percentage of *Caenorhabditis elegans* [77]. 

## 7. Factors Affecting Pigment Production

Decades of research prospects on single-cell prokaryotes to multicellular eukaryotic organisms have corroborated that their life cycles depend on a broad range of physicochemical parameters which regulate or hamper the production of various metabolites. Three major routes are known for the production of any kind of microbial metabolites: (1) Naturally produced metabolites; (2) metabolites produced under unfavourable/strained environmental conditions; and (3) metabolites produced upon stimulation with various carbon, nitrogen, and additional nutrient amendments. The literature implies that the production of different pigment molecules are intra- or extracellular (or both) and dependent on light, pH, temperature, and various media constituents [140] and shifts over time and space such as seasonal factors (alluvial, nival, pluvial), sampling sites and habitats, and different cultivation conditions in the laboratories [49].

**Figure 3:**
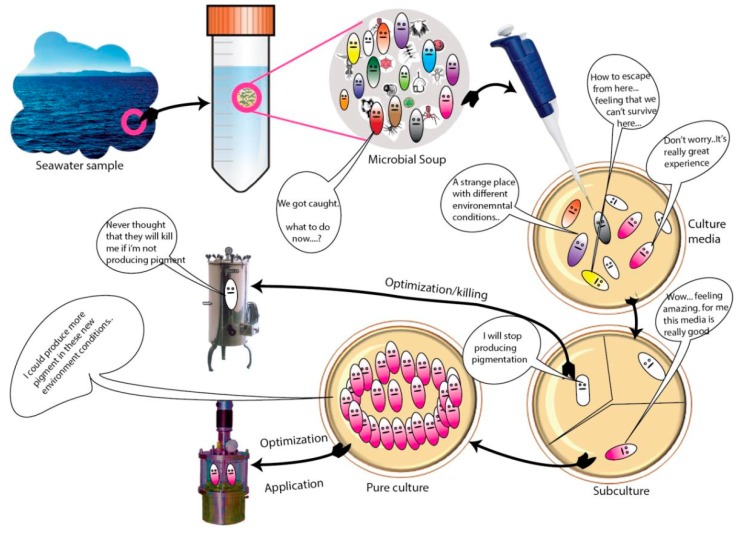
An illustration showing the feelings of microbes.

It is often encountered that microbial pigments, especially bacterial pigmentations, appear is to be ephemeral in nature under laboratory conditions and also when the culture frequently subcultured. However, reverting respective pigmentation may be possible if the culture is supplied with various factors such as environmental parameters and the optimization of medium components (Table 1). Empathizing microbial feelings (e.g., nutrient amendments) in an understandable way is always important for better pigment production research (Figure 3).

Nutrient conditions (richness/poorness) always exert a perceptible effect on pigment synthesis, as earlier studies observed that high phosphate content and high acidity cause diminution of fluorescent pigment and that trace amounts of sulphate can vitiate pigment synthesis [141]. Organic acids produced during *Monascus ruber* culture in oxygen-excess conditions appear to inhibit pigmentation [142]. The addition of several substrates such as rice and wheat meals (either integral or broken residual cereal) and light stimulation have induced high levels of carotenoid production in fungi and yeasts [143]. In 1944, ZoBell and Upham observed that pigmentation was increased when bacteria was grown in sea water enriched with beef extract, bacto-tryptone, and neopeptone at 4 °C. In 1946, ZoBell indicated that the infusions of marine animals such as fish, octopus, and mussel and other animals stimulated pigment production [27]. A study found that pink pigment production in *Acinetobacter wofii* was induced by methanol as sole source of carbon [144]. *Mycobacterium tuberculosis* was found to produce carotenoid pigments in acidic stress at pH 5.0–6.0 and long-term growth in anaerobic culture conditions [145], and several other *Mycobacterium* species appeared to produce different pigments [146].

It was studied that a large amount of water-soluble yellow-green fluorescent pigment synthesis by *P. fluorescens* depends on the addition of succinate as the sole carbon source, and no pigment production was observed upon the addition of citric and malic acids as substrates [63]. Carbon sources such as glucose and inorganic nitrogen sources like ammonium sulphate, peptone, or other amino acids also induce pigmentation. Photochromogenic (photoinduction) and scotochromogenic (pigment formation in the dark) effects on pigment synthesis have been found in nontuberculous Mycobacteria [63]. *Mycobacterium marinum*, *Myxococcus xanthus* (bacteria), *Dacryopinax spathularia* (fungus), and *Rhodotorula glutinis* (yeast) were found to produce carotenoids in the presence of light [63]. Similarly, pigment syntheses by the microalgae appeared to be greatly influence by temperature, salinity, pH, and the light color and intensity [79,147]. 

Observations deduced that many nonpigmented *Thermus* strains at high growth temperatures produce an unstable and irreversible yellow pigmentation product [56]. Some bacterial cultures for instance, e.g., a well-known marine *S. marcescens* produce red pigment on solid peptone-glycerol agar plates, however, failed to produce pigment in a peptone–glycerol liquid medium. Nevertheless, pigmentation was induced in a liquid medium culture when supplemented with silica gel [148]. Chen et al. revealed that elevated levels of prodigiosin production was perceived when supplemented with starch and peptone as carbon source, and significantly, prodigiosin production was increased from 7.05 g/L to 15.6 g/L with the addition of calcium alginate beads as a porous carrier [149]. Increase in pigment production in the cells may be seen when subjected to stress conditions such as high temperature, osmotic pressure, metabolic inhibition, and the existence of heavy metals, etc. [72]. Production of a pigment (e.g., glaukothalin) was found to be linked with the presence of acylated homoserine lactones (AHL), amino acids, and other bacterial strains [135]. Violacein pigment synthesis in *C. violaceum* is regulated by N-acylhomoserine lactone (AHL)-dependent quorum-sensing system [60]. The enhancement of pigment production has been observed upon introduction of mutagens such as UV light, ethyl methane sulfonate, and 1-methyl-3-nitro-1-nitrosoguanidine in *Haematococcus pluvialis* and similarly microwave on *Serratia marcescens* [73]. Further, enhanced pigment production from interested microbe may be achieved by altering genes (gene knockout or promotion) or mutagenesis techniques [72]. To envisage microbial pigments for industrial production, evaluating several suitable substrates and physicochemical parameters for interested pigmented microbe is always an essential step to yield a high amount of pigments for various applications. A detailed description has been reviewed on the extraction of various pigmented compounds from microalgae [9,48,150], fungi [90,103,151], bacteria [49], and yeasts [152]. 

Biosynthesis and expression of pigments in different microorganisms are regulated by respective genes which impart color to the cells. Different substrates such as phenylalanine, tryptophan, and, more significantly, tyrosine were found to be good stimulators for various pigment compounds; however, efforts are to be extended to know other substrates’ efficiency in promoting pigmentation (Table 1).

## 8. Challenges in Pigment Compound Development 

Irrespective of terrestrial or marine origin, to bring out any kind of versatile therapeutic or nutraceutically important microbial pigment products into the market, a lot of investment along with experimental work (e.g., prior assessment of color stability in heat (thermolabile—various temperatures and autoclaving), light (photolysis), pH, agitation, aeration, dissolved oxygen, etc. are most important concerns to be studied for various biotechnological applications), in addition to solubility (e.g., lipolytic, hydrosoluble, and oxidized), optimization process, extensive toxicological studies (e.g., acute oral toxicity in mice 90-day subchronical toxicological study, acute dermal irritation, acute eye irritation, antitumor activity, micronucleus test in mice, AMES test, estimation of antibiotic activity, and estimation of microbial toxins), regulatory approval (e.g., EU and USA legislations, Codex Alimentarius Commission, Food and Drug Administration, European Food Safety Authority, Pharmaceutical and Food Safety Bureau, and National Agency of Sanitary Vigilance), and penchant by the consumers are highly required [21,153,154]. 

Other factors to be considered for desired productivity of microbial pigments in fermentation aspects are type of bioreactor and its design (e.g., traditional bioreactors, stirred-tank and air lift reactor, and trickle-bed reactor), type of fermentation (batch, feed-batch, or continuous), and physicochemical and biological conditions in fermentation process [72]. Upon successful achievement of these requirements, there would be potential demand in the biggest markets for food pigments such as Europe and United States [17]. Storage of pigments is suggested to be in the dry powder form or liquid concentrates, with the former being more preferable due to its low water activity and high stability [143].

Many other pigments are to be commercialized; however, commercialization ventures are found to be hampered by cost-effective synthetic medium, which are being alternatively substituted by the utilization of cheap agro-industrial residues as substrates (e.g., corn meal, peanutmeal, coconut residue, soybean meal, rice water, jackfruit seed, tapioca starch, grape juice, grape must, peat extract, mustard waste, liquid pineapple waste, mung bean waste, sugar beet molasses, corn syrup, starch, cheese whey, minerals, and vitamins) to maximize pigment production [72,153]. Enhancement of pigment production may be effective when culture conditions are optimized with several substrates and using via RSM (Response Surface Methodology) combined with the ANN (Artificial Neural Network) statistical approach [72]. Taskin et al. prepared chicken feather peptone (CFP) from waste chicken feathers and found that CFP induces carotenoid and biomass production about 53 and 36% at 8 g/L CFP concentration respectively [155].

## 9. Pathogenicity of Pigmented Microbes

Regardless of microbial pigments in various applications, some of the pigments produced by certain microorganisms are known to promote pathogenicity and virulence. On sheep blood agar, *P. aeruginosa* is often strongly beta haemolytic and can produce different diffusible pigments such as pyocyanin, a green coloured pigment. *Vibrio campbellii* has been reported to produce a brown pigment which may be due to pyomelanin [129] or proteorhodopsin [156], and this species is a significant pathogen in harveyi clade. Phenazines produced by pseudomonads are known to play a role in virulence [55]. Virulence and pathogenicity in several species of bacteria (e.g., *Vibrio cholerae*) and fungi (e.g., *Cryptococcus neoformans* and *Aspergillus fumigatus*) for their respective animal or plant hosts appeared to be linked with melanin production. *Mycobacterium marinum* is known to cause infections on skin and soft tissues [157], and some of the *Bacillus* species are also reported to be pathogens. *Serratia marcesens* is a well-known agent of nosocomial infections of the urinary tract and wounds [69]. Other pigmented compounds such as golden staphyloxanthin, porphyrin, and granadaene produced by *Staphylococcus aureus*, *Porphyromonas gingivalis*, and *Streptococcus agalactiae*, respectively, are also related to potential virulence functions [69]. Violacein producing *Chromobacterium violaceum* is an opportunistic pathogen for animals and humans and can cause fatal septicemia from skin lesions with many liver and lung abscesses [158]. *Stenotrophomonas maltophiliais* is also an emerging human pathogen that is responsible for fatal infections in humans [159], and orange pigmentation in this species has recently been reported [40]. *Xanthomonas campestris* is a phytopathogenic bacterium which causes diseases in cauliflower, cabbages, and rutabagas. Melanin-like compounds producing *Aeromonas salmonicida* appear to cause fish furunculosis of salmonids [63]. Pyocyanine, a pigmented exotoxin produced by *Pseudomonas aeruginosa* causes chronic lung infections, namely cystic fibrosis in patients [160].

*Rosellinia necatrix* a fungal species was found to infect several plants like *Narcissus* and mulberry and forms white root rot [158]. Melanin producing yeast *Cryptococcus neoformans* is evidently more virulent (a neurotropic pathogen) than albino mutants [63]. *Pencillium marneffei* is found to biosynthesize a mycotoxin called “citrinin” that showed nephrotoxic activity in mammals [161,162]. *Talaromyces purpurogenus* has been reported to produce mycotoxins such as rubratoxin A and B and luteoskyrin in addition to extrolites that may be toxic if injected intraperitoneally (spiculisporic acid) or in the veins of cats (rugulovasine A and B) [163]. Apparently, naphthoquinones from *Fusarium solani* have damaged the plasma membrane of plants [63]. Many other fungal pigments, mostly naphthoquinone metabolites, are found to be phytopathogenic to different plants [164]. 

## 10. Conclusions

Natural pigmented compounds originating from microbial sources like bacteria, fungi, and microalgae are found to be more valuable and demandable over synthetic compounds. Especially, in these days, marine environment is being focused on for the exploration of novel and known natural pigments with a broad range of biological activities due to vast marine resources which are known to harbor several known and novel pigment synthesizing microbes and microalgae. Extending the great exploration on the uncovered samples from new habitats belonging to terrestrial and particularly marine environments would certainly give promising results in finding novel compounds from interested microorganisms. Several pigment microbial species have been reported hitherto, and their biological activities are to be evaluated. Therefore, generating data (biological properties) from uninvestigated microbes as well as novel species are of great importance in understanding their biological activities of pigments to develop novel medicinal compounds for biotechnological applications.

## Figures and Tables

**Table 1:** Various media and supplements required for extraction of specific pigments from different microorganisms.

Pigment	Media/Supplement	Incubation Temperature	Reference
Prodigiosin	Casein hydrolysate agar	24–28 °C	[63]
Violacein	Lactose and tryptophan	22 °C	
Indigo	Potato-glucose-peptone agar, Phosphate agar—incorporation of 2-hydroxypyridine and/or Tryptophan		
Naphthoquinones	Glucose—mineral salt medium with ammonium sulphate, zinc, and magnesium ions—and Glucose—asparagine medium with small amounts of aspartic or glutamic acid and 5-fluorouracil		
Monascus pigments	Suitable media with glucose, peptone or amino acids, and corn and potato starch	25–28 °C	
Pyocyanine	Glycerol, leucine, glycine, alanine, and mineral salts		
Phenazine	Shikimic acid, chorismic acid, glucose, glycerol, gluconate, and glutamine		
Riboflavin	Cornsteep liquor, corn oil, and glycine	26–28 °C	
Melanin	Tyrosine agar, Peptone-yeast extract iron agar, Tyrosine, Zn, Cu, Co, and 3-chlorobenzoate		
Carotenoids	Mevalonic acid, trisporic acid, and Isopentenyl pyrophosphate		
Anthraquinones	Sucrose, molasses, corn extract, yeast extract, zinc sulfate, and magnesium sulphate	27–29 °C	21

## Supplementary Materials

The following are available online at https://www.mdpi.com/2076-2607/7/7/186/s1, Table S1: Pigmented compounds of different microorganisms and their biological properties.
