# Supplementary material for: Multifaceted Applications of Microbial Pigments: Current Knowledge, Challenges and Future Directions for Public Health Implications
Source: Microorganisms. 2019 Jun 28;7(7):186. doi: 10.3390/microorganisms7070186 (PMC6680428; doi:10.3390/microorganisms7070186)
Supplement: Supplementary file 1 [file microorganisms-07-00186-s001.pdf]

Table S1. Pigmented compounds of different microorganisms and their biological properties.

Genus/Species	Color	Pigment Compound	Application	Reference
**Prokaryotes**				
**Gram-negative bacteria**				
Achromobacter sp.	Orange red-pink			Duerre and Buckley, 1965
Acinetobacter lwofii	Pink	Bacterioruberin-like	Antioxidant activity	Ghosh et al., 2007
Aeromonas salmonicida	Brown	Melanin		Margalith, 1992
Agrobacterium arantiacum	Pink-red	Astaxanthin, adonixanthin	Food additive, antioxidant	Yokoyama et al., 1995
Allochromatium vinosum	Orange brown	Anhydrorhodovibrin		Kirti et al., 2014
Allochromatium warmingii	Pink-violet			Kirti et al., 2014
Altererythrobacter ishigakiensis	Orange-red	Astaxanthin, adonixanthin and zeaxanthin		Matsumoto et al., 2011
Alteromonas citrea	Lemon yellow	Non Carotenoid	Antibiotic property	Gauthier, 1977
Alteromonas (*Pseudomonas*) *nigrifaciens*	Blue	Indigoidine		Norton and Jones, 1969
Alteromonas nigrifaciens		Eumelanins (black or brown), phaeomelanins (yellow–red), allomelanins, and pyomelanins	Protection from UV irradiation	Liu and Nizet 2009; Soliev et al., 2011
Alteromonas rubra	Red	Prodigiosin	Antibiotic, immunosuppressive and anticancer activities	Williamson et al., 2007
Aureobacterium sp., Arthrobacter glacialis, Cellulomonas biazotea, Corynebacterium glutamicum	Yellow	Decaprenoxanthin		Fukuoka et al., 2004

Azotobacter chroococcum	Black	Catechol melanin		Margalith, 1992
Bacillus thuringiensis subsp. *israelensis*	Black	Melanin	Mosquitocidal activity	Liu et al.,1993
Bradyrhizobium sp.	Orange-red	Canthaxanthin	Impart color in farmed salmons	Kirti et al., 2014
Burkholderia cepacia	Black-brown	Melanin		Kirti et al., 2014
Burkholderia glumae	Yellow	Toxoflavin	Antifungal, phytotoxic activity	Karki et al., 2012
Cellulophaga lytica	Iridescent blue, violet, red, yellow, and green			Kientz et al., 2012
Cellulophaga tyrosinoxydans	Yellow	Phaeomelanin		Kahng et al., 2009
Chlorobiaceae	Brown-green	Chlorobactene, renieratene, renierapurpurin		Grice et al., 1998; Schaeflé et al., 1977
Chlorobium	Green	Bacteriochlorophyll *c,d,e*		Montano et al., 2003
Chlorobium tepidum	Yellow	Chlorobiumquinone		Frankenberg et al., 1996
Chloronema	Green , red	Bacteriochlorophyll *a*, keto-myxocoxanthin (Deoxyflexixanthin)		Takaichi et al., 2001a
Chromatium sp.	Green, red	Bacteriochlorophyll *a*, Spirilloxanthin, rhodovibrin, rhodopsin, Okenone		Madigan, 1986
Chromobacterium violaceum	Purple	Violacein (3-(1,2-dihydro-5-(5-hydroxy-1H-indol-3-yl)-2-oxo-3H-pyrrol-3-ilydene)-1,3-dihydro-2H-indol-2-one)	Anmicrobial activity	Rettori and Durán, 1998
Chryseobacterium	Yellow- ornage	Flexirubin	Used in treatment of	Venil et al., 2015

			chronic skin disease, eczema etc; antioxidant, antimicrobial	
Collimonas sp.	Blue/purple	Violacein	Antibacterial activity	Hakvåg et al., 2009
Colwellia	Red	Prodigiosin-like		Bowman et al., 1998
Cytophaga/Flexibacteria	Yellow	Tryptanthrin	Antibiotic activity	Wagner-Döbler et al., 2002
Cytophaga uliginosa	Yellow-red	Flexirubin	Cytochrome *c* oxidase activity	Bowman, 2000
Desulfovibrio	Green	Siroheme	Cytoplasmic respiratory sulfite reduction	Simon and Kroneck, 2013
Desulfovibrio desulfuricans (*Desulfomicrobium norvegicum*)	Red	Desulforubidin	Sulfite reducing activity	Lee et al., 1973
Desulfovibrio sp.	Blue to green, fluorescent	Desulfoviridin		Warren et al., 2005
Dinoroseobacter shibae	Pink-red	Bacteriochlorophyll *a*, and spheroidene		Endres et al., 2015
Dokdonia sp.	Yellow-orange	Salinixanthin, Zeaxanthin		Bertsova et al., 2016
Enterobacter sp.	Red	β-carotene	Used as feed supplement for hens or other avian	Tanskul et al., 2013
Erwinia chrysanthemi	Blue	Indigoidine	Antioxidant	Reverchon et al., 2002
Flavobacterium sp.	Yellow-red	Flexirubin	Photoprotective compound	Reichenbach et al. 1974
Gemmatimonas aurantiaca	Ornage	Carotenoids (oscillol 2,2′-dirhamnoside)		Kirti et al., 2014
Hahella chejuensis	Red	Prodigiosin	Antibacterial and algicidal activities	Kim et al., 2007
Hyphomonas sp.	Brown	Pyomelanin		Plonka and Grabacka, 2006

Hyphomonas strain	Brown-black	Melanin		Kotob et al., 1995
Iodobacter fluviatile	Violet			Logan, 1989
Janthinobacterium lividum	Bluish-purple	Violacein	Antibacterial, algicidal activity	Matz et al., 2004
Janthinobacterium lividum	Violet	Violaceinand deoxyviolacein	Antibacterial activity	Nakamura et al.,2003
Janthinobacterium svalbardensis	Violet	Violacein-like		Avgustin et al., 2013
Kiloniella	Yellow-orange	Phenazine	Antimicrobial activities	Schneemann et al. 2011
Klebsiella sp.	Black	Melanin		Shrishailnath et al., 2010
Lamprocystis roseopersicina	Rosy pink			Kirti et al., 2014
Leisingera sp.	Blue	Indigoidine	Antibacterial	Gromek et al., 2016
Marinomonas mediterranea	Black	Eumelanin		Plonka and Grabacka, 2006
Marinomonas mediterranea	Black	Melanins	Photoprotective and antioxidant	Solano et al., 1997; Solano and Sanchez-Amat, 1999
Nanocystis exedens	Yellow-orange	Carotenoids		Margalith, 1992
Pantoea agglomerans	Blue			Fujikawa and Akimoto, 2011
Paracoccus carotinifaciens	Pink to red	Astaxanthin	Fish feed supplement in USA	Malik et al., 2012
Paracoccus haeundaensis	Ornage-red	Astaxanthin		Lee et al., 2004
Paracoccus zeaxanthinifaciens	Yellow	Zeaxanthin		Malik et al., 2012
Pararhodospirillum		Bacteriochlorophyll *a*, and carotenoids lycopene and rhodopsin, rhodopin		Baldani et al., 2014
Pelagibacter	Yellow-orange	Phenazine	Antibacterial	Choi et al., 2010
Photobacterium ganghwense	Pink		Antibacterial	Ramesh et al., 2017
Potobacterium,	Blue-green	Luciferin	Toxicity assays,	Ramesh and Mohanraju,

Shewanella, Vibrio,			bioluminescence imaging	2015
Photobacterium kishitanii, Vibrio azureus	Blue	Lumazine (BFP)		Yoshizawa et al., 2012
Photorhabdus luminescens	Yellow	Anthraquinone	Antibiotic activity	Li et al., 1995
Polaribacter sp.		Proteorhodopsin	Light harvest and generate a proton gradient across the cytoplasmic membrane	Gonzȧlez et al., 2008
Pontibacter	Pink	Menaquinone-7		Joshi et al., 2012
Porphyromonas canoris, Porphyromonas gingivalis	Black	Porphyrin	Antioxidant	Smalley et al., 1998
Protomonas extorquens	Red	Rhodoxanthin	Feed additive	Nelis and de Leenheer, 1991
Pseudoalteromonas strain 520P1	Purple	Violacein	Cytotoxic activity against U937, K562, and HL60 leukemia cell lines	Yada et al., 2008; Zhang and Enomoto, 2011
Pseudoalteromonas strains, *Chromobacterium*	Yellow	Tetrabromopyrrole	Antibacterial, Inducing larval metamorphosis	Tebben et al., 2011; Andersen et al., 1974
Pseudoalteromonas denitrificans, Pseudoalteromonas rubra	Red	Cycloprodigiosin	Apoptotic effects on liver cancer cell lines; antimalarial	Yamamoto et al., 1999; Kim et al., 1999
Pseudoalteromonas flavipulchra, Pseudoalteromonas maricaloris, Pseudoalteromonas prydzensis, Pseudoalteromonas rubra	Yellow	Bromoalterochromide A and B	Cytotoxic activities	Speitling et al. 2007
Pseudoalteromonas luteoviolacea	Violet	Violacein	Antibacterial activity and cytotoxicity	Kobayashi et al., 2007

Pseudoalteromonas rubra	Yellow-red	Prodigiosins (2-methyl-3-butyl-prodiginine, 2-methyl-3-pentyl-prodiginine (prodigiosin), 2-methyl-3-hexyl-prodiginine, and 2-methyl-3-heptyl-prodiginine); cycloprodigiosin	cytotoxic effects against U937, K562, and HL60 leukemia cancer cells	Wang et al., 2012
Pseudoalteromonas tunicata		alkaloid tambjamines	antitumor activity, Antimicrobial and cytotoxic activities	Franks et al., 2005; Pinkerton et al. 2010
Pseudomonas aeruginosa	Blue	Pyocyanin	Antibiotic activity	Angell, 2006
Pseudomonas aeruginosa	Brown	Pyorubin	Antibacterial activity	Saha et al., 2008
Pseudomonas aeruginosa	Brown	Pyomelanin		Plonka and Grabacka, 2006
Pseudomonas aeruginosa	Yellow-orange	Phenazine	Colorant in beverages, cakes, confectionaries, pudding, decoration of food items	Isnansetyo and Kamei 2009; Saha et al., 2008
Pseudomonas aeruginosa		Oxychlororaphine		Margalith, 1992
Pseudomonas aeruginosa	Yellow-green	Fluorescein	Coloring agent	Margalith, 1992
Pseudomonas aeruginosa, Pseudomonas aureofaciens	Yellow	Phenazine-1-carboxylic acid		Margalith, 1992
Pseudomonas aeruginosa	Yellow	Pyoverdine	Pathogenesis and biofilm formation	Peek et al., 2012
Pseudomonas argentinensis	Yellow			Peix et al., 2005
Pseudomonas aurantiaca	Yellow	Bromoalterochromide A and B	Cytotoxic activities	Speitling et al., 2007
Pseudomonas aureofaciens	Golden yellow			Margalith, 1992
Pseudomonas brassicacearum	Orange	Phloroglucinol derivatives	Antimicrobial	Ivanova et al., 2009

			activity	
Pseudomonas cepacia	Purple	4,9-di-hydroxyphenazine-1,6-dicarboxylic acid dimethyl ester		Korth et al., 1978
Pseudomonas chlororaphis	Green	Chlororaphine	Antioxidant	Margalith, 1992
Pseudomonas fluorescens	Yellow-green fluorescent	Pyoverdine		Meyer and Abdallah, 1978
Pseudomonas indigofera	Blue	Indigoidine	Dyeing properties	Knackmuss, 1973
Pseudomonas magnesiorubra	Red	Prodigiosin	Antimicrobial activity	Lewis and Corpe, 1964
Pseudomonas magnesiorubra	Orange-yellow	Magnesidin	Antimicrobial activity	Kohl et al., 1974
Pseudomonas paucimobilis		Nostoxanthin		Jenkins et al., 1979
Pseudomonas phenazinium	purple	Iodinin		Byng and Turner, 1976
Pseudomonas syringae	Blue	Pyocyanine		Margalith, 1992
Pseudovibrio	Yellow-orange	Phenazine	antimicrobial activities	Schneemann et al., 2011
Ralstonia eutropha	Blue	Indigo		Drewlo et al., 2001
Ralstonia solanacearum	Red	Melanin	Pathogenic activity	Hernández-Romero et al., 2005
Rheinheimera strains	Deep blue	Glaukothalin	antibacterial and cytotoxic activities	Grossart et al., 2009
Rhizobium sp.	Brownish black	Melanin		Margalith, 1992
Rhodobaca bogoriensis		Bacteriochlorophyll *a*, demethylspheroidene and demethylspheroidenone		Takaichi et al., 2001b
Rhodobacter sphaeroides		Bacteriochlorophyll *a*, neurosporene, spheroidene		Polívka et al., 2007

Rhodovulum sulfidophilum	Green	Chloroxanthin	Color based whole-cell biosensors	Yoshida et al., 2007
Phaeobacter, Roseobacter	Blue	Indigoidine	Antibacterial	Cude et al., 2012
Rubritalea squalenifaciens		acyclic C30 –type carotenoic acids, diapolycopenedioic acid	Antioxidant activity	Misawa, 2011
Rugamonas rubra	Red	Prodigiosin	Antibacterial activity	Kirti et al., 2014
Salinibacter ruber	Red	Carotenoids		Antón et al., 2002
Saprospira grandis	Orange-red	Xanthophyll, Saproxanthin	Antioxidant	Aasen and Liaaen-Jensen, 1966
Serratia marcescens	Red	Prodigiosin	Antibacterial, Antifungal, Anti *Entamoeba histolytica*, Immunosuppressive properties, in vitro apoptotic effects, and in vivo anti-tumor activities	Margalith, 1992; Chen et al., 2013
Serratia marinorubra	Red	Prodigiosin	Antibacterial Activity	Margalith, 1992
Serratia plymuthica	Red	Prodigiosin	Antibacterial Activity	Grimont and Grimont, 1991
Serratia rubidaea	Red	Prodigiosin	Antibacterial Activity	Moss, 2002
Shewanella algae, Shewanella colwelliana, Shewanella hanedai	Dark brown	Pyomelanin		Kotob et al., 1995
Shewanella colwelliana	Black-brown	Eumelanins (black or brown), phaeomelanins (yellow–red), allomelanins, and pyomelanins	Protection from UV irradiation	Liu and Nizet 2009; Soliev et al., 2011; Plonka and Grabacka 2006

Shewanella violacea	Violet	5,5′-didodecylamino-4,4′-dihydroxy-3,3′-diazodiphenoquinone-(2,2′) (Indigoidine)	Dyeing application	Kobayashi et al., 2007
Sorangium	Red	Myxin	Antimicrobial, antifungal	Sekhon and Hargesheimer, 1975
Sphingomonas astaxanthinifaciens	Red	Astaxanthin	Antioxidant	Asker et al., 2007
Stenotrophomonas maltophilia	Orange		Antibacterial	Ramesh et al., 2017
Stigmatella aurantiaca	Yellow& red	Myxobactin, myxobactone		Kleinig et al., 1970
Thermus filiformis		C_{50} carotenoids	Antioxidant	Mandelli et al., 2012
Thioalkalivibrio versutus	Yellow	Natronochrome and chloronatronochrome		Takaichi et al., 2004
Thiocapsa roseopersicina	Rosy peach			Kirti et al., 2014
Thiocystis violacea, Thiodictyon elegans	Violet			
Unidentified marine bacterium	Yellow	2-methyl-pyrimidine-5-carboxamide		Laatsch, 2006
Vibrio sp.	Red	Prodigiosin	Antimicrobial activity	Rameshkumar and Nair, 2009
Vibrio sp.	Blue			Ramesh et al., 2017
Vibrio cholerae		Melanin, Homogentisic acid		Kotob et al., 1995; Ruzafa et al., 1995
Vibrio cholerae		Eumelanins (black or brown), phaeomelanins (yellow–red), allomelanins, and pyomelanins	Protection from UV irradiation	Liu and Nizet 2009; Soliev et al., 2011
Vibrio fischeri	Yellow	Yellow fluorescent protein		Herring, 2002

		(YFP) chromophore		
Vibrio gazogenes	Red	Prodigiosin like	Antibacterial Activity	Moss, 2002
Vibrio gazogenes	Orange-yellow	Magnesidin	Antialgal	Imamura et al., 1994
Vibrio psychroerythrus	Red	Prodigiosin, Zeaxanthin	Antibacterial Activity	Kirti et al., 2014
Vibrio ruber	Red	Prodigiosin	Antibacterial Activity	Shieh et al., 2003
Vogesella sp.	Deep blue		Colorant	Cardona-Cardona et al., 2010
Xanthomonas campestris	Yellow	Xanthan	Food additive & Antibacterial	Qian et al., 2006
Xanthomonas oryzae	Yellow	Xanthomonadin	Photoprotective pigment; Chemotaxonomic and diagnostic markers	Venil et al., 2013; Rajagopal et al., 1997
Zobellia	Yellow-red	Flexirubin		Nedashkovskaya et al., 2004
Zooshikella ganghwensis, Zooshikella marina Zooshikella rubidus	Red	Cycloprodigiosin-and prodigiosin	Antimicrobial activity	Ramaprasad et al., 2015 ; Lee et al., 2011
Marine α-proteobacterium	Red	Heptylprodigiosin	Cytotoxic activity, antimalarial activity	Lazaro et al., 2002
**Gram-positive bacteria**				
Arthrobacter atrocyaneus	Blue	Indigoidine	Dyeing properties	Knackmuss, 1973
Arthrobacter crystallopoietes	Dark green	Indigoidine	Colorant	Margalith, 1992
Arthrobacter oxidans	Blue	Nicotine	Colorant	Knaekmuss and Beekmann, 1973
Arthrobacter polychromogenus	Blue	Indigoidine	Dyeing properties	Knackmuss, 1973
Bacillus sp.	Yellow-pink	Carotenoids	Antioxidants	Khaneja et al., 2010
Bacillus	Yellow-orange	Phenazine	Antibacterial activity	Choi et al., 2010
Bacillus indicus,	Yellow-orange	Diapocarotenoids		Pérez-Fons and Fraser,

Halobacillus,<br>*Heliobacteria,*<br>*Methylobacterium rhodinum,*<br>*Planococcus,*<br>*Staphylococcus aureus,*<br>*Streptococcus faecium*				2012
Bacillus indicus	Yellow-orange	Glycosyl-diapolycopene		Pérez-Fons and Fraser, 2012
Bacillus licheniformis	Red	Diadinoxanthin		Nugraheni et al., 2010
Bacillus subtilis	Black		Antibiotic activity	Nakamura, 1989
Bacillus subtilis	Yellow	Riboflavin	Food colorant	Stahmann et al., 2000
Clostridium puniceum	Pink		Pectolytic activity	Lund et al., 1981
Deinococcus radiodurans	Red	Deinoxanthin	Antioxidant Activity	Hong-Fang, 2010
Dietzia (Rhodococcus) maris	Bluish Red			Joshi et al., 2003
Exiguobacterium	Yellow, orange	Carotenoid	Antioxidant and antibacterial	Balraj et al., 2014
Micrococcus sp.	Red	Prodigiosin like	Antioxidant activity	Variyar et al., 2002
Micrococcus luteus	Yellow		Antibacterial activity	Umadevi and Krishnaveni, 2013
Micrococcus roseus	Orange to pink	Canthaxanthin		Cooney et al., 1966
Micrococcus yunnanensis	Red	sarcinaxanthin, sarcinaxanthin mono-glucoside, and sarcinaxanthin diglucoside	Antioxidative activities	Osawa et al., 2010
Micromonospora	Yellow-orange	Phenazine	Antimicrobial activities	Schneemann et al., 2011
Planococcus maritimus	Red	11lycol-carotenoic acid ester and methyl glucosyl-3,4-dehydro-apo-8′-	Antioxidant	Shindo et al., 2008

		lycopenoate		
Planococcus maritimus		acyclic C30 –type carotenoic acids		Misawa, 2011
Staphylococcus aureus	Golden	Staphyloxanthin	Antioxidant	Clauditz et al., 2006
Staphylococcus aureus	Yellow	Carotenoids		Marshall and Wilmoth , 1981
Streptococcus agalactiae	Ornage-red	Granadaene	Antioxidant	Rosa-Fraile et al., 2006
**Actinomycetes**				
Actinomycete isolate CNB-632	Red	Marinone	Antibacterial activity	Pathirana et al., 1992
Actinomadura madurae	Red	Prodigiosin like		Gerber, 1969
Brevibacterium	Yellow-orange	Phenazine	Antibacterial activity	Choi et al., 2010
Brevibacterium iodinum	purple	Iodinin		Margalith, 1992
Brevibacterium linens	Yellow-red	Isorenieratene	Antioxidant activity	Martin et al., 2009
Corynebacterium michigannise	Greyish to creamish			Joshi et al., 2003
Gordonia jacobaea	Red	Canaxanthin	Food colorant and cosmetic application	Veiga-Crespo et al., 2012
Microbacterium arborescens	Orange	Lycopene		Godinho and Bhosle, 2008
Mycobacterium abscessus, Mycobacterium avium, Mycobacterium chelonae, Mycobacterium fortuitum, Mycobacterium goodie, Mycobacterium smegmatis, Mycobacterium tuberculosis		Carotenoids		Saviola, 2014
Mycobacterium aureus		Escholtzxanthin		Margalith, 1992
Mycobacterium aurum	Yellow-red	Isorenieratene	Antioxidant activity	Martin et al., 2009
Mycobacterium chubuense	Yellow	Zeaxanthin, escholtzxanthin		Margalith, 1992

Mycobacterium kansasii, Mycobacterium marinum		Carotenoids		Margalith, 1992
Mycobacterium lacticola	Red	Astaxanthin	Fish feeds	Kirti et al., 2014
Mycobacterium phlei	Yellow	Isorenieratene (leprotene)	Antioxidant activity	Margalith, 1992
Nocardia sp.	Blue-black	Indigo		Margalith. 1992
Nocardia madurae	Red	Prodigiosin	Antibacterial activity	Gerber and Lechevalier, 1976
Nocardia pelletierie				
Pseudonocardia sp.	Yellow	Phenozostatin D	Antibacterial and antifungal activities	Maskey et al., 2003
Streptomyces sp.		Dihydrophencomycin	Antibiotic activity	Pusecker et al., 1997
Streptomyces sp.	Yellow	Streptochlorin	Anticancer activity	Karuppiah et al., 2013
Streptomyces sp.	Blue	Ammosamide A		
Streptomyces sp.	Red	Ammosamide B		
Streptomyces sp.	Yellow	N-carboxamidostaurosporine		
Streptomyces sp.	Blue	Akashin	Antitumor activity	Maskey et al., 2002
Streptomyces sp.	Yellow	Griseolutein	Antibacterial activity	Umezawa et al., 1950
Streptomyces sp.	Red	Panosialin	Anti-influenza activity	Aoyagi et al., 1971
Streptomyces sp.	Red	Marineosin A	Cytotoxic effect, antifungal	Boonlarppradab et al., 2008
Streptomyces sp.	Brown-black	Melanin	Antimicrobial activity	Vasanthabharathi et al., 2011
Streptomyces sp.	Red	Hyaluromycin	Hyaluronidase inhibitory activity	Harunari et al., 2014
Streptomyces amakusaensis, Streptomyces antibioticus, Streptomyces biverticillatus, Streptomyces glaucescens, Streptomyces kentuckensis,	Brownish black	Melanin		Margalith, 1992

Streptomyces lavendulae, Streptomyces lucensis, Streptomyces violaceus, Streptomyces violaceorectus				
Streptomyces aureofaciens	Blue-green	Indigoidine		Kirti et al., 2014
Streptomyces chibanensis	Black –brown	Melanin		Kirti et al., 2014
Streptomyces coelicolor	Blue	Actinorhodins	Antibiotic	Bystrykh et al. 1996
Streptomyces coelicolor	Red, blue	Prodigiosin, undecylprodigiosin and actinorhodin	Antimicrobial activity	Ahmad et al., 2012
Streptomyces coelicolor	Red-brown	Flaviolin	Melanin production	Thanapipatsiri et al., 2015
Streptomyces davawensis	Pink/reddish orange	Roseoflavin	Antibacterial compound	Otani et al., 1974
Streptomyces diastaticus	Yellow	Diastaphenazine	cytotoxic and antibacterial activities	Li et al., 2014
Streptomyces echinoruber	Red	Rubrolone	Antibiotic activity and food colorant	Iacobucci and Sweeney, 1981
Streptomyces griseoviridis	Reddish purple	Roseophilin	Cytotoxic effect	Hayakawa et al., 1992
Streptomyces lincolnensis	Dark	Lincomycin (melanin like)	Antibacterial	Margalith, 1992
Streptomyces longisporubei	Red	Prodigiosin	Antimicrobial activity	Gerber and Lechevalier, 1976
Streptomyces longisporus	Red	Prodigiosin	Antibacterial activity	Variyar et al., 2002
Streptomyces mediolani	Yellow-red	Isorenieratene	Antioxidant activity	Martin et al., 2009
Streptomyces parvulus	Orange red	Actinomycin D	Antibacterial activity	Shetty et al., 2014
Streptomyces pilosus	Red	Piloquinone		Margalith, 1992
Streptomyces spectabilis	Red	Prodigiosin	Antibacterial activity	Variyar et al., 2002
Streptomyces variegatus	Red		Antibacterial activity	Lee et al., 2011
Streptomyces viridoflavus	Blue	Candidin	Antifungal activity	Taber et al., 1954
Streptoverticillium rubrireticuli	Red	Prodigiosin like	Antimicrobial	Gerber and Lechevalier,

			activity	1976
Thermoactinomyces antibioticus	Orange-red	Thermorubin	Antibacterial activity	Moppett et al., 1971
**Archaea**				
Halobacterium salinarium, Halococcus morrhuae	Red	Bacterioruberin	Antioxidant activity	Mandelli et al., 2012
Halobacterium halobium	Red	Bacteriorhodopsin		Matsuno-Yagi and Mukohata, 1977
Halobacterium salinarium, Halococcus morrhuae		C_{50} carotenoids	Antioxidant	Mandelli et al. 2012
Haloferax alexandrinus	Dark Red	Canthaxanthin		Malik et al., 2012
Haloferax mediterranei		Carotenoid	Antitumor, antioxidant, food colorants and cosmetic application	Rodrigo-Baños et al., 2015
**Cyanobacteria**				
Acaryochloris marina	Green	Chlorophyll *d*		Roy et al., 2011
Anabaena	Blue-red	Phycobiliproteins	Antiox-idant, anti-inflammatory, neuroprotective and hepatopro-tective properties	Hemlata and Fatma, 2009
Anabaena planctonica		Carotenoids	Antioxidant	Guedes et al., 2013
Anabaena variabilis	Blue	C-phycocyanin	antiviral, anticancer, antioxidant, antifungal and antibacterial	Sharma and Kaur, 2016
Arthronema africanum	Blue	C-phycocyanin and allophycocyanin	Antioxidant , anti-Inflammatory and neuroprotective effects	Chaneva et al., 2007; Romay et al., 2003

Aulosira fertilissima	Yellow	Aulosirazole	Antitumor activity	Řezanka and Dembitsky, 2006
Chlorococcus giganteus		Carotenoids	Antioxidant	Guedes et al., 2013
Chlorogloeopsis	Black	Scytonemin	Antioxidant	Jehlička et al., 2014; Wada et al., 2013
Chlorogloeopsis fritschii		Caloxanthin		Roy et al., 2011
Cyanobacteria		Antheraxanthin, Aphanicin, Aphanizophyll, canthaxanthin, iso-cryptoxanthin, Flavacin, oscilloxanthin, mutachrome, myxoxanthophyll		Hertzberg and Jensen, 1967
Cyanobacteria	Yellow green	Scytonemin	Anti-proliferative, anti-inflammatory activities	Stevenson et al., 2002
Cyanophytes	Pink-reddish	Cyanophycin, phycocyanobilin, phycoerythrobilin		Van den Hoek et al., 1995
Cyanothece sp.		Carotenoids	Antioxidant	Guedes et al., 2013
Leptolyngbya sp.	Red	Phycoerythrin	Antioxidative activity	Pumas et al., 2012
Limnothrix sp.	Blue	C-phycocyanin	Antioxidant	Gantar et al., 2012
Lyngbya, *Leptolyngbya*	Blue	Phycocyanin	antioxidant activity	Guedes et al., 2013
Lyngbya aestuarii	Black	Scytonemin	Antioxidant	Edwards et al., 2000; Wada et al., 2013
Lyngbya majuscule		Carotenoids	antioxidant activity	Guedes et al., 2013
Nodularia harveyana				

Nostoc sp.	Black	Scytonemin	Antioxidant	Wada et al., 2013
Nostoc carneum		Carotenoids	Antioxidant	Guedes et al., 2013
Nostoc commune	Red-violet	Canthaxanthin, Mycosporine		Bohm et al., 1995
Nostoc paludosum	Blue, red	Phycocyanin , Phycoerythrin, Allophycocyanin		Moreno et al., 1995
Nostoc punctiforme		Carotenoids	Antioxidant	Guedes et al., 2013
Nostoc spongiaeforme	Violet	Nostocine A	Antimicrobial, antifungal, herbicidal activities	Hirata et al., 1996
Oscillatoria	Red	Phycoerythrin	Antioxidant, anti-aging proprties	Chu et al., 2002; Sonani et al., 2015
Oscillatoria agardhii	Blue	C-phycocyanin, Echinenone	Antioxidant	Millie et al., 1990
Oscillatoria irrigua	Red	Phycourobilin		Stadnichuk et al., 1985
Phormidium	Blue	Phycocyanin	antioxidant activity	Patel et al., 2006
Prochlorococcus		divinyl-chlorophyll *a* and *b*, chlorophyll *c*-like pigment, zeaxanthin, and α-carotene		Takaichi et al., 2012
Phormidium tenue	Red	C-phycoerythrin		MubarakAli et al., 2012
Scytonema obscurum		Carotenoids	Antioxidant	Guedes et al., 2013
Spirulina maxima	Blue-red	Phycobiliproteins	Fluorescent labels in immunoassays	Tomasseli et al., 1997; Kronick,1986
Spirulina (Arthrospira) platensis	Light blue	Phycocyanin	Anti-oxidative, hypo-cholesterolemic activity, anti-inflammatory, cytotoxicity effects	Boussiba and Richmond, 1980; Walter et al., 2011

			and fluorescent agent in immunoassay analysis	
Stigonema sp.	yellow-green	Scytonemin	Ultraviolet sunscreen pigment	Haefner, 2003
Symploca sp.	Red	Dolastatin	Antitumor activity	Luesch et al., 2001; Soliev and Enomoto, 2013
Synechococcus elongates	Blue	C-phycocyanin	Antiviral, anticancer, antioxidant, antifungal and antibacterial	Sharma and Kaur, 2016
Synechocystis	Blue-red	Phycobiliproteins	Fluorescent labels in immunoassays	Hong and Lee, 2008; Kronick,1986
Synechocystis salina	Yellow-red	β-carotene , Lutein	Antioxidant	Guedes et al., 2013
Trichodesmium		Carotenoid	Antioxidant	Ananya et al., 2014
**Eukaryotes**				
**Fungi**				
Agaricus bisporus	Black	Melanin		Margalith, 1992
Agaricus bisporus	Black	GHB-melanin	Cryptobiosis and cytotoxicity	Vogel et al., 1977
Albatrellus confluens	Red	Grifolinone	Cytotoxic activity	Yang et al., 2008
Aleuria aurantia	Red	Aleuriaxanthin		Margalith, 1992
Alternaria alternata	Yellow	Fonsecin		Shaaban et al., 2012
Alternaria tagetica	Reddish orange	Alterperylenol, dihydroalterperylenol	Antifungal activity	Okuno et al., 1983
Ashbya gossypii	Yellow	Riboflavin (Vitamin B2)	food colorant	Stahmann et al., 2000
Aspergillus sp., *Monascus ruber*, *Penicillium citrinum*, *Phomopsis vexans*,	Red	Mevinolin (lovastatin)	Lowers blood cholesterol	Parthasarathy and Sathiyabama, 2015

Pleurotus ostreatus				
Aspergillus niger	Yellow	Aurasperone A	Antibacterial, anticancer	Song et al., 2004
Aspergillus cristatus	Yellow-Red	Catenarin, emodin, physcion, erythroglaucin, rubrocristin, questin	Food colorants and antibiotic properties	Caro et al., 2012
Aspergillus fonsecaeus (carbonarius)	Yellow	Fonsecin		Priestap, 1984
Aspergillus glaucus	Yellow-Red	Emodin, catenarin, cynodontin, helminthosporin, tritisporin, physcion (parietin), erythroglaucin, aspergiolide A, Physcion-9-anthrone, Catenarin, Viocristin, Rubrocristin,	Antimicrobial Activity	Caro et al., 2012
Aspergillus melleus, Aspergillus sulphureus	Yellow-red	Xanthomegnin, viomellein, rubrosulphin, and viopurpurin		Durley et al., 1975
Aspergillus nidulans, Aspergillus niger	Black	Melanin		Margalith, 1992
Aspergillus oryzae	Orange-red	Anthraquinone group		Joshi et al., 2003
Aspergillus repens	Yellow-red	Erythroglaucin, physcion	Food colorants	Caro et al., 2012
Aspergillus versicolor	Yellow	Sterigmatocystin (Vuillemin)	Cytotoxic activity	Piontek et al., 2016
Auxarthron umbrinum	Red	rumbrin	Anticancer	Clark and Murphy, 2009
Blakeslea trispora		β-carotene, Lycopene	Antioxidant	Papaioannou and Liakopoulou-Kyriakides 2010
Boletus sp.	Blue-yellow	Atromentin, grevillin A, variegatorubin, pulvinic		Nelsen, 2010

		acid, and vulpinic acid		
Botrytis cinerea	Grayish	DHN-melanin	Growth and virulence	Schumacher, 2016
Cantharellus cinnabarinus		Canthaxanthin	Poultry feeds and fish feeds	Kirti et al., 2014
Cercospora piaropia	Red	Cercosporin	Herbicide	Jiménez et al., 2010
Chaetomium globosum *Chaetomium cochliodes*	Purple	Cochliodinol	Antibacterial and antifungal	Meiler and Taylor, 1970
Chroogomphus rutilus	Pink	Boviquinone 3		Jiménez et al., 2010
Chlorociboria aeruginosa, Chlorociboria aeruginascens	Blue-green	Xylindein	Antialgicidal & textile dyes	Sakaki et al., 2002; Hinsch et al., 2015
Cladosporium sp.		Melanin		Margalith, 1992
Claviceps purpurea	Yellow-red	Ergoxanthin		Hooper et al., 1971
Cochliobolus miyabeanus		Melanin		Margalith, 1992
Colletotrichum lagenarium, Colletotrichum lindemuthianum		Melanin		Margalith, 1992
Cordyceps sp.	Yellow	Cordycepin	anti-cancer, anti-oxidant and anti-inflammatory activities	Tuli et al., 2014
Cordyceps bifusispora	Yellow	Cordycepoid A	Edible	Lu et al., 2013
Cortinarius	Yellow-red	Emodin, dermocybin, dermorubin, flavomannin	Food colorants and antibiotic properties	Zalas et al., 2015
Cryptococcus neoformans	Black-brown	Melanin		Kirti et al., 2014
Curvularia lunata	Red	Cynodontin	Antifungal	Gessler et al., 2013
Curvularia lunata	Yellow-red	Catenarin, chrysophanol, cynodontin, erythroglaucin, helminthosporin, tritisporin	Food colorants and antibiotic properties	Caro et al., 2012

Cylindrocarpon sp.(LL-Cyan426)	Yellow	Pyrrocidine A	Antibacterial, cytotoxicity	Uesugi et al., 2016
Dermocybe sp.		Phallacinol	Antibacterial and antifungal activities	Gessler et al., 2013
Dermocybe austroveneta	Red fluorescent	Hypericin-like		Margalith, 1992
Dermocybe sanguinea	Yellow-red	dermocybin-1-β-D-glycopyranoside; dermorubin; ermolutein; dermoglaucin; 5-chlorodermorubin; emodin & physcion	Food colorants and antibiotic activities	Caro et al., 2012
Drechslera avenae	Red	Cynodontin	Antifungal	Gessler et al., 2013
Drechslera dictyoides, Drechslera graminea, Drechslera phlei, Drechslera teres, Drechslera triticirepentis	Red	Catenarin	Food colorant and antiobiotic activity	Caro et al., 2012
Elsinoe fawcetti	Red	Elsinochromes	Cytotoxicity	Liao and Chung, 2008
Epicoccum nigrum	Purple	Epicocconone	Natural fluorescent probe	Bell and Karuso, 2003
Fungi K_BK5	Red	Austrocortinin		Caro et al., 2012
Fusarium	Red	Javanicin	Fungitoxicity, antibacterial, insecticidity, phytotoxicity, membrane modification, metal chelating	Margalith, 1992
Fusarium sp.	Yellow-red	catenarin, cynodontin, erythroglaucin,	Food colorants	Caro et al., 2012

		helminthosporin, physcion, tritisporin		
Fusarium, *Nectria haematococca*		Fusarubin	Fungitoxicity, antibacterial, insecticidity, phytotoxicity, membrane modification, metal chelating	Margalith, 1992
Fusarium solani	Red	karuquinone A	Cytotoxic and anticancer activity	Takemoto et al., 2014
Gomphidius glutinosus	Yellow	Gomphidic acid		Knight and Pattenden, 1979
Helminthosporium catenarium, *Helminthosporium graminium*	Red	Anthraquinonoid		Joshi et al., 2003
Herpotrichia rhodosticta	Orange	Averythrin and averythrin-6-monomethyl ether	Food colorants	Caro et al., 2012
Isaria farinosa	Red	Hydroxyanthraquinoid		
Lactarius sp.	Blue	Azulenes		Harmon et al., 1980
Lactarius deliciosus	Red	Dihydroxyazulene, Acetylazulene, Lactaroviolin		Rai, 2009
Lactarius indigo	Red	Lactaroviolin		Nelsen, 2010
Lactarius indigo	Blue	Stearoyldeterrol		Nelsen, 2010
Lactarius lilacinus	Red	Lilacinone		Spiteller et al., 2003
Laetiporus sulphureus		Laetiporic acid A	Antimicrobial and antioxidant	Popa et al., 2016
Magnaporte grisea	Brown-black	Melanin		Margalith, 1992
Melanogaster broomeianus	Yellow	Melanocrocin		Aulinger et al., 2001

Microsporum cookei	Yellow	Luteosporin, Floccosin, iridosporin, rubrosporin		Kawai and Nozawa, 1982
Monascus	Orange	Lovastatin (mevinolin)	Anti-hypercholesteremic	Goswami et al., 2012
Monascus anka	Yellow	Ankaflavin	Antimicrobial, anticancer , anti-obesity	Feng et al., 2016
Monascus purpureus	Red	Monascorubramin	Cytotoxic effect	
Monascus sp.	Orange	Rubropunctatin	Antimicrobial	
Monascus purpureus	Orange	Monascorubrin, monascoflavin	Antibacterial, antifungal	
Monascus purpureus	Red	Rubropunctamine	Cytotoxic effect	
Monascus pilosus	Yellow	Monascin	Anticancer, anti-obesity	
Monascus ruber	Red	Monarubrin, Rubropunctin	Antimicrobial	
Mycena aurantiomarginata	Red	Mycenaaurin A (polyene)	antibacterial activity	Jaeger and Spiteller, 2010
Mycena haematopus	Red	Haematopodin		Baumann et al., 1993
Mycena sanguinolenta	Blue	Sanguinone A		Peters and Spiteller, 2007
Nectria haematococca	Red	Bostrycoidin	antituberculotic and antimicrobial activities	Awakawa et al., 2012
Nectria haematococca	Yellow	Nectriachrysone	Antibiotic related	Parisot et al., 1991
Neurospora crassa	Yellow-orange	β-carotene	Antioxidant, cosmetic colorant	Priatni, 2014
Pachybasium candidum	Yellow-red	Pachybasin, chrysophanol	Food colorants	Caro et al., 2012
Penicilliopsis clavariaeformis	Red fluorescent	Hypericin-like		Margalith, 1992
Pencillium, Aspergillus	Purple	Spinulosin	Antibacterial	Margalith, 1992
Pencillium albidum	Red	Albidin	Antifungal	Tisler, 1989
Pencillium islandicum	Yellow-red	Skyrin, emodin	Food colorants and	Caro et al., 2012

			antibiotic properties	
Pencillium chrysogenum	Yellow	Chrysogenin		
Penicillium citrinum	Yellow-red	Citrinin, emodin	Antibacterial &Cytotoxic activity	Subramani et al., 2013
Penicillium marneffei	Red	Monascorubrin	Natural food colorant	Woo et al., 2014
Penicillium oxalicum	Red	Arpink Red (Anthraquinoid)	Anticancer activity	Dufossé, 2006; Sardaryan 2006
Penicillium purpurogenum	Red	Azaphilones	Food colorants	Carle and Schweiggert, 2016
Pestalotia sp.	Yellow	Pestalone	Antibiotic	Cueto et al., 2001
Phoma saccardo, Phoma sorghina	Yellow-red	Anthraquinones	Herbicidal and Mycopesticidal activities	Rai et al., 2009
Phoma exigua var. *foveata*	Yellow-Red	Pachybasin, Emodin, Chrysophanol, and Phomarin	Food colorants and bioactive properties	Caro et al., 2012
Phoma lingam and *Phoma wasabiae*	Yellow	Phomaligin A	Phytotoxic activity	Pedras et al., 1995
Phycomyces blakesleeanus	Yellow-ornage	β-carotene	Antioxidant	Tagua et al., 2012
Pycnoporus sanguineus	Red	Phenoxazine (Cinnabarin)	Cytotoxic and antiviral activity	Smânia Jr et al., 2003
Pyricularia oryzae	Dark	Melanin		Margalith, 1992
Ramularia collocygni	Red	Rubellin D	Phytotoxic activity	Heiser et al., 2003
Schizophyllum commune	Blue	Indigotine (Indigo)	Cytotoxic activity	Hosoe et al., 1999
Schizophyllum commune	Black-brown	Melanin	Antibacterial, antifungal, antioxidant and antiproliferative	Arun et al., 2015
Scytalidium cuboideum	Red	Draconin	Textile dyes	Hinsch et al., 2015

Scytalidium ganodermophthorum	Yellow		Textile dyes	Hinsch et al., 2015
Suillus bovinus	Yellow-orange	Amitenone	Antibiotic activity or anti-virus effect	Asawa and Minami, 1971
Suillus grevillei	Orange	Aurantricholide B, pyrandione and furanones		Velíšek and Cejpek, 2011
Suillus grevillei	Yellow	Grevilline A		Shmuel, 2004
Talaromyces albobiverticillius, Talaromyces marneffei, Talaromyces minioluteus, Talaromyces purpurogenus,	Red	Monascus-like	Antibacterial, food colorants	Yilmaz et al., 2014
Talaromyces atroroseus	Red	Mitorubrins, Glauconic acid, Purpuride	food colorant	Frisvad et al., 2013
Thielaviopsis basicola		Melanin		Margalith, 1992
Trichoderma aureoviride	Yellow-red	Pachybasin, chrysophanol	Food colorants	Caro et al., 2012
Trichoderma harzianum	Yellow-red	Pachybasin, chrysophanol		
Trichoderma reesei	Yellow	Sorbicillin	Antifungal activity	Basaran and Demirbas, 2010
Trichoderma viride	Yellow-red	Pachybasin, chrysophanol, emodin &1,3,6,8-tetraHAQN,2,4,5,7-tetraHAQN	Food colorants, bioactive properties	Caro et al., 2012
Trichophyton rubrum	Yellow-purple	Melanoid		Zussman et al., 1960
Trichophyton violaceum	Yellow-red	Xanthomegnin, viopurpurin, vioxanthin		Ng et al., 1969
Ustilago maydis	Brown-black	Melanin		Margalith, 1992
Verticillium dahlia				
Wangiella dermatitidis				
Verticillium dahlia	Yellow	Juglone	Cytotoxic activity	Babula et al., 2009

Xylaria euglossa	Green-yellow	Phlegmacin A		Wang et al., 2004
Other fungal species		Naphthoquinones	Phytopathogenic	Medentsev and Akimenko, 1998
**Yeast**				
Candida famata (*Debaryomyces hansenii*)	Yellow	Riboflavin	food colorant	Stahmann et al., 2000
Candida lipolytica	Yellow	Tryptanthrin	Antibiotic activity	Wagner-Döbler et al., 2002
Cryptococcus neoformans	Black	Melanin	Antioxidant	Casadevall and Perfect, 1998
Cryptococcus neoformans	Black/brown	DOPA melanin		Butler and Day, 1998
Kluyveromyces marxianus	Black	Melanin		Kirti et al., 2014
Phaeococcomyces sp.	Black	Melanin		Margalith, 1992
Phaffia rhodozyma	Pink-red	Astaxanthin	Feed supplement for salmons, crabs, shrimps, chickens, and egg production	Kirti et al., 2014
Pichia (*Candida*) *guilliermondii*	Yellow	Riboflavin	food colorant	
Rhodotorula	Orange-red	Carotenoids, torulene, torularhodin		Margalith, 1992
Sporidiobolus		Carotenoids	Antioxidant	Konuray and Erginkaya, 2015
Sporobolomyces ruberrimus				
Yarrowia lipolytica	Brown-red	Lycopene	Antioxidant , anticancer, anti-inflammatory	Nambou et al., 2015
Other species		Carotenoids	Antioxidant	Yurkova et al., 2008
**Lichens**				
Acarospora chlorophana	Yellow	Rhizocarpic acid		Lu et al., 2011
Caloplaca sp.	Yellow-red	2-chloroemodin, citreorosein, emodin,	Food grade colorants and antibiotic effects	Caro et al., 2012

		fallacinal, phallacinol, parietin, parietin, physcion, teloschistin		
Caloplaca cerina	Yellow	Emodin, fallacinal, physcion, teloschistin		
Caloplaca erythrantha	Yellow	Emodin, 7-chloroemodin		
Candelariella sp.	Red	Calycin		Edwards et al., 2003
Cladonia sp.	Red	Skyrin	Food grade colorants and antibiotic effects	Caro et al., 2012
Dirinaria aegialita	Red	Gyrophoric acid		Jehlička et al., 2014
Heteroderma obscurata	Yellow	Emodin	Food grade colorants and antibiotic effects	Caro et al., 2012
Laurera benguelensis	Yellow-red	Emodin, fallacinal, parietin, physcion, citreorosein, teloschistin		
Lepraria sp.	Yellow-red	Atranorin		Edwards et al., 2003
Nephroma laevigatum		Emodin, 7-chloroemodin	Food grade colorants and antibiotic effects	Caro et al., 2012
Teloschistes sp.	Yellow-red	Emodin, erythroglaucin, fallacinal, parietin, teloschistin, parietinic acid		
Teloschistes exilis	Yellow	Parietin, teloschistin		
Xanthoria sp.	Yellow-red	Citreorosein, emodin, erythroglaucin, fallacinal, fallacinol, physcion, parietin, teloschistin (phallacinol or fallacinol)		
Xanthoria fallax	Yellow-red	Emodin, erythroglaucin, fallacinal, fallacinol, parietin		
Xanthoria mandschurica	Red, yellow	Erythroglaucin, parietin		
Xanthoria parietina	Yellow-red	Emodin, fallacinol,		

		fallacinal, parietinic acid, Physcion		
**Microalgae**				
Amphidinium carterae		Dinoxanthin		Roy et al., 2011
Chlorella fusca, Chlorella zofingiensis, Chlorella protothecoides, Chlorella vulgaris, Chlorococcum citroforme, Coelastrum proboscideum, Muriella aurantiaca, Muriella decolor, Neospondiococcum gelatinosum, Tetracystis aplanosporum, Tetracystis intermedium, Tetracystis tetrasporum	Yellow	Lutein	Food colorant, anticancer	Dufossé, 2016
Chlorella ellipsoidea	Yellow	Zeaxanthin	Antiproliferative effect	Cha et al., 2008
Chlorella vulgaris		Carotenoids	Antioxidant	Guedes et al., 2013
Chlorella zofingiensis	Red	Astaxanthin	Food additive, antioxidant	Ip and Chen, 2005
Chlorococcum	Yellow	Lutein	Feed additive, disease prevention	Campo et al., 2007
Chlorophyta	Red	Prasinoxanthin		Graham and Wilcox, 2000
Chlorophyta		Siphonoxanthin, siphonein		Van den Hoek et al., 1995
Chondrus crispus	Blue, red	Phycocyanin, Phycoerythrin		Franklin et al., 2002
Chroomonas salina	Brown	Alloxanthin		Cheng et al., 1974
Cryptophyta		Crocoxanthin, monadoxanthin		Van den Hoek et al., 1995

Diatomophyceae (Diatoms)	Red	Diadinoxanthin		Graham and Wilcox, 2000
Dinoflagellates (Dinophyta)	Red	Peridinin		Van den Hoek et al., 1995
Dinoflagellates	Blue-green	Luciferin	Toxicity assays, bioluminescence imaging	Ramesh and Mohanraju, 2015
Dinophyta	Orange	Diatoxanthin, dinoxanthin, pyrroxanthin		Van den Hoek et al., 1995
Dunaliella sp.	Orange-red	Carotenes	Anticancer, antibacterial, antiviral, anti-inflammatory, anti-allergic, anti-atherosclerosis activities	Arun and Singh, 2016
Dunaliella salina	Yellow-red	β-carotene, lycopene, phytoene, bacterioruberin, and salinixanthin	Antioxidant activity	Hosseini and Shariati, 2009
Dunaliella tertiolecta		β-carotene , Chlorophyll *a*, Auroxanthin, Mutatoxanthin	Antihypertensive activity	Fox, 1985; Roy et al., 2011
Euglenophyta	Red	Diadinoxanthin, Neoxanthin		Lee, 1999
Eutreptiella gymnastica		Eutreptiellanone		Roy et al., 2011
Galdieria sulphuraria	Yellow	Lutein	Antioxidant	Graziani et al., 2013
Gymnodinium galatheanum	Yellow	Gyroxanthin diester		Bjørnland et al., 2000
Haematococcus pluvialis	Red	Astaxanthin	Antioxidant, nutraceuticals, food, cosmetics, and aquaculture industries	Shah et al., 2016

Haslea ostrearia	Blue	Marennine	antibacterial, antiviral, anticancer	Gastineau et al., 2012
Heterokontophyta	Orange	Diatoxanthin	Antioxidant	Van den Hoek et al., 1995
Muriellopsis sp.	Yellow	Lutein	Feed additive	Campo et al., 2007
Neospongiococcum excentricum	Yellow-orange	Zeaxanthin	Colorant for poultry and fish	Kirti et al., 2014
Pavlova lutheri		Carotenoids	Antioxidant	Guedes et al., 2013
Phaeodactylum tricornutum	Orange	Fucoxanthin	Antioxidant	Guedes et al., 2013
Phorphyridium aerugineum	Red	Phycoerythrin	Cosmetics	Sonani et al., 2016
Porphyridium aerugineum		Carotenoids	Antioxidant	Guedes et al., 2013
Pyramimonas parkeae	Yellow	Loroxanthin		Roy et al., 2011
Raphidophyceae		Heteroxanthin, vaucherixanthin, violaxanthin		Van den Hoek et al., 1995
Scenedesmus almeriensis, Scenedesmus capricornutum, Scenedesmus maximus, Scenedesmus obliquus, Scenedesmus quadricauda	Yellow	Carotenoid, Lutein	Antioxidant activity	Guedes et al., 2013
Siphonocladales, Codiales	Red	Siphonaxanthin	Antioxidant activity	Van den Hoek et al., 1995
Spongiococcum excentricum	Yellow	Lutein	As poultry feed	Kirti et al., 2014
Takayama	Brown-Red	Fucoxanthin, violaxanthin, diadinoxanthin, diatoxanthin, gyroxanthin diester		De Salas et al., 2003
Trentepohliaceae, Palmella	Red	Carotenoids (Hematochrome)		Sheath and Wehr, 2003
**Other eukaryotic microbes**				
Plasmodium falciparum (a protozoan)	Brown	Hemozoin	Triggers immune complex	Olivier et al., 2014

Thraustochytrium (a protist)	Orange-red	astaxanthin, zeaxanthin, canthaxanthin, echinenone, phoenicoxanthin (adonirubin), and β-carotene	Antioxidant activity	Carmona et al., 2003

Supplementary References

1. Aasen, A.J., Liaaen-Jensen, S. 1966. The carotenoids of flexibacteria II. A new xanthophyll from *Saprospira grandis*. Acta Chemica Scandinavica. 20, 811-819.
2. Ahmad, W.A., Ahmad, W.Y.W., Zakaria, Z., Yusof, N.Z. 2012. Application of bacterial pigments as colorant: the Malaysian perspective, in: Briefs in molecular science. Springer, Heidelberg, pp. 57-74.
3. Angell, S., Bench, B.J., Williams, H., Watanabe, C.M.H. 2006. Pyocyanin isolated from a marine microbial population: Synergistic production between two distinct bacterial species and mode of action. Chemistry & Biology. 13, 1349-1359.
4. Antón, J., Oren, A., Benlloch, S., Rodriguez-Valera, F., Amann, R., Rossello-Mora, R. 2002. *Salinibacter ruber* gen. nov., sp. nov., a novel, extremely halophilic member of the Bacteria from saltern crystallizer ponds. International Journal of Systematic and Evolutionary Microbiology. 52, 485-491.
5. Aoyagi, T., Yagisawa, M., Kumagai, M., Hamada, M., Okami, Y., Takeuchi, T., Umezawa, H. 1971. An enzyme inhibitor, panosialin, produced by *Streptomyces*. I. Biological activity, isolation and characterization of panosialin. Journal of Antibiotics. 24, 860-869.
6. Arun, G., Eyini, M., Gunasekaran, P. 2015. Characterization and biological activities of extracellular melanin produced by *Schizophyllum commune* (Fries). Indian Journal of Experimental Biology. 53, 380-387.
7. Arun, N., Singh, D.P. 2016. A review on pharmacological applications of halophilic alga *Dunaliella*. Indian Journal of Marine Sciences. 45, 440-447.
8. Asawa, K., Minami, K. 1971. The synthesis of the hydrogenated derivative of Amitenone a methylenebisbenzoquinone from *Suillus bovinus*. Journal of Wood Science. 17, 384-392.
9. Asker, D., Beppu, T., Ueda, K. 2007. *Sphingomonas astaxanthinifaciens* sp. nov., anovel astaxanthin-producing bacterium of the family Sphingomonadaceae isolated from Misasa, Tottori, Japan. FEMS Microbiology Letters. 273, 140-148.
10. Aulinger, K., Besl, H., Spiteller, P., Spiteller, M., Steglich, W. 2001. Melanocrocin, a polyene pigment from *Melanogaster broomeianus* (Basidiomycetes). Zeitschrift für Naturforschung. 56C, 495-498.
11. Avgustin, J.M., Bertok, D.Z., Kostanjsek, R., Avgustin, G. 2013. Isolation and characterization of a novel violacein-like pigment producing psychrotrophic bacterial species *Janthinobacterium svalbardensis* sp. nov. Antonie van Leeuwenhoek. 103, 763-769.
12. Awakawa, T., Kaji, T., Wakimoto, T., Abe, I. 2012. A heptaketide naphthaldehyde produced by a polyketide synthase from *Nectria haematococca*. Bioorganic & Medicinal Chemistry Letters. 22, 4338-4340.
13. Babula, P., Adam, V., Havel, L., Kizek, R. 2009. Noteworthy Secondary Metabolites Naphthoquinones – their Occurrence, Pharmacological Properties and Analysis. Current Pharmaceutical Analysis. 5, 47-68.
14. Baldani, J., Videira, S.S., et al. 2014. The Family Rhodospirillaceae, in: Rosenberg E., et al. (eds.), The Prokaryotes – Alphaproteobacteria and Betaproteobacteria. Springer-Verlag, Berlin Heidelberg, pp. 533-618.

15. Balraj, J., Pannerselvam, K., Jayaraman, K. 2014. Isolation of pigmented marine bacteria *Exiguobacterium* sp. from the Peninsular Region of India and a study on biological activity of purified pigment. International Journal of Scientific & Technology Research. 3(3), 375-384.
16. Basaran, P., Demirbas, R.M. 2010. Spectroscopic detection of pharmaceutical compounds from an aflatoxigenic strain of *Aspergillus parasiticus*. Microbiological Research. 165, 516-522.
17. Baumann, C., Brockelmann, M., Fugmann, B., Steffan, B., Steglich, W., Sheldrick, W.S. 1993. Haematopodin, an unusual pyrroloquinoline derivative isolated from the fungus *Mycena haematopus*, Agaricales. Anxrw Chm Inf Ed Engl. 32, 1087-1089.
18. Bell, P.J.L., Karuso, P. 2003. Epicocconone, a novel fluorescent compound from the fungus *Epicoccum nigrum*. Journal of the American Chemical Society. 125, 9304-9305.
19. Bertsova, Y.V., Arutyunyan, A.M., Bogachev, A.V. 2016. Na^+ -translocating rhodopsin from *Dokdonia* sp. PRO95 does not contain carotenoid antenna. Biochemistry. 81, 414-419.
20. Bjørnland, T., Fiksdahl, A., Skjetne, T., Krane, J., Liaaen-Jensen, S. 2000. Gyroxanthin-the first allenic acetylenic carotenoid. Tetrahedron. 56, 9047-9056.
21. Bohm, G.A., Pfleiderer, W., Boger, P., Scherer, S. 1995. Structure of a novel oligosaccharide-mycosporine-amino acid ultraviolet A/B sunscreen pigment from the terrestrial cyanobacterium *Nostoc commune*. Journal of Biological Chemistry. 270, 8536-8539.
22. Boonlarppradab, C., Kauffman, C.A., Jensen, P.R., Fenical, W. 2008. Marineosins A and B, Cytotoxic Spiroaminals from a Marine-Derived Actinomycete. Organic Letters. 10, 5505.
23. Boussiba, S., Richmond, A.E. 1980. C-phycocyanin as a storage protein in the blue-green alga *Spirulina platensis*. Archives of Microbiology. 125, 143-147.
24. Bowman, J.P., Gosink, J.J., McCammon, S.A., et al. 1998. *Colwellia demingiae* sp. nov., *Colwellia hornerae* sp. nov., *Colwellia rossensis* sp. nov. and *Colwellia psychrotropica* sp. nov. : psychrophilic Antarctic species with the ability to synthesize docosahexaenoic acid (22 : 6ω3). International Journal of Systematic Bacteriology. 48, 1171-1180.
25. Bowman, J.P. 2000. Description of *Cellulophaga algicola* sp. nov., isolated from the surfaces of Antarctic algae, and reclassification of *Cytophaga uliginosa* (ZoBell and Upham 1944) Reichenbach 1989 as *Cellulophaga uliginosa* comb. nov. International Journal of Systematic and Evolutionary Microbiology. 50, 1861-1868.
26. Butler, M.J., Day, A.W. 1998. Fungal melanins: a review. Canadian Journal of Microbiology. 44, 1115-1136.
27. Byng, G.S., Turner, J.M. 1976. Isolation of Pigmentation Mutants of *Pseudomonas phenazinium*. Journal of General Microbiology. 97, 57-62.
28. Bystrykh, L.V., FernandezMoreno, M.A., Herrema, J.K., Malpartida, F., Hopwood, D.A., Dijkhuizen, L. 1996. Production of actinorhodin-related 'blue pigments' by *Streptomyces coelicolor* A3(2). Journal of Bacteriology. 178, 2238-2244.
29. Campo, A.J.D., García-González, M., Guerrero, M.G. 2007. Outdoor cultivation of microalgae for carotenoid production: current state and perspectives. Applied Microbiology and Biotechnology. 74, 1163-1174.
30. Cardona-Cardona, V., Arroyo, D., Scellekens, J., Rios-Velazquez, C. 2010. Characterization of blue pigmented bacteria isolated from Puerto Rico, in: Méndez-Vilas, A. (ed.), Current Research, Technology and Education Topics in Applied Microbiology and Microbial Biotechnology. FORMATEX. pp. 117-123.

31. Carle, R., Schweiggert, R. 2016. Handbook on Natural Pigments in Food and Beverages: Industrial Applications for Improving Food Color. Woodhead Publishing.
32. Carmona, M.L., Naganuma, T., Yamaoka, Y. 2003. Identification by HPLC-MS of Carotenoids of the *Thraustochytrium* CHN-1 Strain Isolated from the Seto Inland Sea. Bioscience, Biotechnology, and Biochemistryem. 67, 884-888.
33. Casadevall, A., Perfect, J.R. 1998. *Cryptococcus neoformans*. ASM Press, Washington, D.C.
34. Cha, K.Y., Koo, S.Y., Lee, D. 2008. Antiproliferative Effects of Carotenoids Extracted from *Chlorella ellipsoidea* and *Chlorella vulgaris* on Human Colon Cancer Cells. Journal of Agricultural and Food Chemistry. 56, 10521-10526.
35. Chaneva, G., Furnadzhieva, S., Minkova, K., Lukavsky, J. 2007. Effect of light and temperature on the cyanobacterium *Arthronema africanum* a prospective phycobiliprotein producing strain. Journal of Applied Phycology. 19, 537-544.
36. Cheng, J.Y., Don-Paul, M., Antia, N.J. 1974. Isolation of an unusually stable cis-isomer of alloxanthin from a bleached autolysed culture of *Chroomonas salina* grown photo-heterotrophically on glycerol. Observations on cis-trans isomerization of alloxanthin. Journal of Protozoology. 21, 761-768.
37. Choi, E.J., Kwon, H.C., Ham, J., Yang, H.O. 2010. 6-Hydroxymethyl-1-phenazine-carboxamide and 1,6-Phenazinedimethanol from a marine bacterium, *Brevibacterium* sp. KMD 003, associated with marine purple vase sponge. ChemInform. 41, 1349-1359.
38. Chu, W.L., Alwi, A., Phang, S.M. 2002. Phycoerythrin production by a marine *Oscillatoria* (Cyanophyta). Malaysian Journal of Science. 21, 67-73.
39. Clark, B.R., Murphy, C.D. 2009. Biosynthesis of pyrrolylpolyenes in *Auxarthron umbrinum*. Organic and Biomolecular Chemistry. 7, 111-116.
40. Clauditz, A. et al. 2006. Staphyloxanthin plays a role in the fitness of *Staphylococcus aureus* and its ability to cope with oxidative stress. Infection and Immunity. 74, 4950-4953.
41. Cooney, J.J., Marks Jr, H.W., Smith, A.M. 1966. Isolation and Identification of Canthaxanthin from *Micrococcus roseus*. Journal of Bacteriology. 92, 342-345.
42. Cude, W.N., Mooney, J., Tavanaei, A.A., Hadden, M.K., Frank, A.M., Gulvik, C.A., May, A.L., Buchan, A. 2012. Production of the antimicrobial secondary metabolite indigoidine contributes to competitive surface colonization by the marine Roseobacter *Phaeobacter* sp. Strain Y4I. Applied and Environmental Microbiology. 78, 4771-4780.
43. Cueto, M., Jensen, P.R., Kauffman, C., Fenical, W., Lobkovsky, E., Clardy, J. 2001. Pestalone, a new antibiotic produced by a marine fungus in response to bacterial challenge. Journal of Natural Products. 64, 1444-1446.
44. De salas, M.F., Bolch, C.J.S., Botes, L., Nash, G., Wright, S.W., Hallegraeff, G.M. 2003. *Takayama* gen. nov. (Gymnodiniales, Dinophyceae), a new genus of unarmored dinoflagellates with sigmoid apical grooves, including the description of two new species. Journal of Phycology. 39, 1233-1246.
45. Drewlo, S., Brämer, C.O., Madkour, M., Mayer, F., Steinbüchel, A. 2001. Cloning and expression of a *Ralstonia eutropha* HF39 gene mediating indigo formation in *E. coli*. Applied and Environmental Microbiology. 67, 1964-1969.
46. Duerre, J.A., Buckley, P.J. 1965. Pigment production from tryptophan by an *Achromobacter* species. Journal of Bacteriology. 90, 1686-1691.

47. Dufossé, L. 2006. Microbial production of food grade pigments. Food Technology and Biotechnology. 44, 313-321
48. Durley, R.C., MacMillan, J., Simpson, T.J., Glen, A.T., Turner, W.B. 1975. Fungal products. Part XIII. Xanthomegnin, viomellin, rubrosulphin, and viopurpurin, pigments from *Aspergillus sulphureus* and *Aspergillus melleus*. Journal of the Chemical Society. 2, 163-169.
49. Edwards, H.G.M., Garcia-Pichel, F., Newton, E.M., Wynn Wiliams, D.D. 2000. Vibrational Raman spectroscopic study of scytonemin, the UV-protective cyanobacterial pigment. Spectrochimica Acta Part A. 56, 193-200.
50. Edwards, H.G.M., Newton, E.M., Wynn-Williams, D.D., Lewis-Smith, R.I. 2003. Nondestructive analysis of pigments and other organic compounds in lichens using Fourier-Transform Raman spectroscopy: A study of Antarctic epilithic lichens. Spectrochimica Acta Part A. 59, 2301-2309.
51. Endres, S., Granzin, J., Circolone, F., Stadler, A., et al. 2015. Structure and function of a short LOV protein from the marine phototrophic bacterium *Dinoroseobacter shibae*. BMC Microbiology. 15, 30.
52. Frankenberg, N., Hager-Braun, C., Feiler, U., Fuhrmann, M., Rogl, H., Schneebauer, N., Nelson, N., Hauska, G. 1996. P840-reaction centers from *Chlorobium tepidum*-Quinone analysis and functional reconstitution into lipid vesicles. Photochemistry Photobiology. 64, 14-19.
53. Franklin, L.A., Kräbs, G., Kuhlenkamp, P. 2002. Blue light and UV radiation control the synthesis of mycosporine like amino acids in *Chondrus crispus* (Floridiophyceae). Journal of Phycology. 37, 257-270.
54. Fujikawa, H., Akimoto, R. 2011. New Blue Pigment Produced by *Pantoea agglomerans* and Its Production Characteristics at Various Temperatures. Applied and Environmental Microbiology. 77, 172-178.
55. Fukuoka, S., Ajiki, Y., Ohga, T., Kawanami, Y., Izumori, K. 2004. Production of dihydroxy C50-carotenoid by *Aureobacterium* sp. FERM P-18698. Biosci. Biotechnol. Biochem. 68(12): 2646-2648.
56. Gantar, M., Simovic, D., Djilas, S., Gonzalez, W.W., Miksovska, J. 2012. Isolation, characterization and antioxidative activity of C-phycocyanin from *Limnothrix* sp. strain 37-2-1. Journal of Biotechnology. 159, 21-26.
57. Gastineau, R., Pouvreau, J., Hellio, C., et al. 2012. Biological Activities of Purified Marennine, the Blue Pigment Responsible for the Greening of Oysters. Journal of Agricultural and Food Chemistry. 60, 3599-3605.
58. Gauthier, M. J. 1977. *Alteromonas citrea,* a new gram-negative, yellow-pigmented species from seawater. International Journal of Systematic Bacteriology. 27, 349-354.
59. Gerber, N.N. 1969. Prodigiosin-like pigments from *Actinomadura* (*Nocardia*) *pelletieri* and *Actinomadura madurae*. Applied Microbiology. 18, 1-3.
60. Gerber, N.N., Lechevalier, M.P. 1976. Prodiginine (prodigiosin-like) pigments from *Streptomyces* and other aerobic Actinomycetes. Canadian Journal of Microbiology. 22, 658-667.

61. Godinho, A., Bhosle, S. 2008. Carotenes produced by alkaliphilic orange- pigmented strain of Microbacterium arborescens - AGSB isolated from coastal sand dunes. Indian Journal of Marine Sciences. 37: 307-312.
62. Gonzålez, J.M., Fernandez-Gomez, B., Fernandez-Guerra, A., Gomez-Consarnau, L., Sanchez, O., et al. 2008. Genome analysis of the proteorhodopsin-containing marine bacterium *Polaribacter* sp. MED152 (Flavobacteria). PNAS. 105, 8724-8729.
63. Goswami, S., Vidyarthi, A.S., Bhunia, B., Mandal, T. 2012. A review on lovastatinand its production. Journal Of Biochemical Technology. 4, 581-587.
64. Graziani, G., Schiavo, S., Nicolai, M.A., Buono, S., Fogliano, V., et al. 2013. Microalgae as human food: chemical and nutritional characteristics of the thermo-acidophilic microalga *Galdieria sulphuraria*. Food & Function. 4, 144-152.
65. Grice, K., Schouten, S., Peters, K.E., Damsté, J. S. 1998. Molecular isotopic characterisation of hydrocarbon biomarkers in Palaeocene-Eocene evaporitic, lacustrine source rocks from the Jianghan Basin, China. Organic Geochemistry. 29, 1745-1764.
66. Grimont, F., Grimont, P.A.D. 1991. The genus *Serratia*, In: Balows, A., Truper, H.G., et al. (eds.), Prokaryotes, Vol. 3. Springer, New York, pp. 2822-2848.
67. Gromek, S.M., Suria, A.M., Fullmer, M.S., Garcia, J.L., Gogarten, P., Nyholm, S.V., et al. 2016. *Leisingera* sp. JC1, a bacterial isolate from Hawaiian bobtail squid eggs, produces indigoidine and differentially inhibits vibrios. Frontiers in Microbiology. 7, 1342.
68. Guedes, A.C., Gião, M.S., Seabra, R., Ferreira, A.C.S, Tamagnini, P., Moradas-Ferreira, P., Malcata, F.X. 2013. Evaluation of the antioxidant activity of cell extracts from microalgae. Marine Drugs. 11, 1256-1270.
69. Haefner, B. 2003. Drugs from the deep: Marine natural products as drug candidates. Drug Discovery Today. 8, 536-544.
70. Hakvåg, S., Fjærvik, E., Klinkenberg, G., Borgos, S.E.F., Josefsen, K.D., Ellingsen, T.E., Zotchev, S.B. 2009. Violacein-producing *Collimonas* sp. from the sea surface microlayer of costal waters in Trøndelag, Norway. Marine Drugs. 7, 576-588.
71. Harmon, A.D., Weisgraber, K.H., Weiss, U. 1980. Preformed azulene pigments of *Lactarius indigo* (Schw.) Fries (Russulaceae, Basidiomycetes). Experientia. 36, 54-56.
72. Harunari, E., Imada, C., Igarashi, Y., Fukuda, T., Terahara, T., Kobayashi, T. 2014. Hyaluromycin, a new hyaluronidase inhibitor of polyketide origin from marine *Streptomyces* sp. Marine Drugs. 12, 491-507.
73. Hayakawa, Y., Kawakami, K., Seto, H., Furihata, K. 1992. Structure of a new antibiotic, roseophilin. Tetrahedron Lett. 33, 2701.
74. Heiser, I., Sachs, E., Liebermann, B. 2003. Photodynamic oxygen activation by rubellin D, a phytotoxin produced by *Ramularia collocygni* (Sutton et Waller). Physiological and Molecular Plant Pathology. 62, 29-36.
75. Hemlata, Fatma, T. 2009. Screening of cyanobacteria for phycobiliproteins and effect of different environmental stress on its yield. Bulletin of Environmental Contamination and Toxicology. 83, 509-515.
76. Hernández-Romero, D., Solano, F., Sanchez-Amat, A. 2005. Polyphenol Oxidase Activity Expression in *Ralstonia solanacearum*. Appl. Environ. Microbiol. 71(11): 6808-6815.

77. Herring, P. 2002. Marine microlights: the luminous marine bacteria. Microbiol. Today. 29: 174-176.
78. Hertzberg, S., Jensen, S.L. 1967. The carotenoids of blue-green algae-III. A comparative study of mutatochrome and flavacin. Phytochemistry. 7, 1119-1126.
79. Hinsch, E.M.; Chen, H-L.; Weber, G.; Robinson, S.C. 2015. Colorfastness of extracted wood-staining fungal pigments on fabrics: a new potential for textile dyes. Journal of Textile and Apparel, Technology and Management. 9(3): 1-11.
80. Hirata, K., Takashina, J., Nakagami, H., Ueyama, S., Murakami, K., Kanamori, T., Miyamoto, K. 1996. Growth inhibition of various organisms by a violet pigment nostocine A, produced by *Nostoc spongiaeforme*. Bioscience, Biotechnology, and Biochemistry. 60, 1905-1906.
81. Hong, S.J., Lee, C.G. 2008. Statistical optimization of culture media for production of phycobiliprotein by *Synechocystis* sp. PCC 6701. Biotechnology and Bioprocess Engineering. 13, 491-498.
82. Hong-Fang, J. 2010. Insight into the strong antioxidant activity of deinoxanthin, a unique carotenoid in *Deinococcus radiodurans*. International Journal of Molecular Sciences. 11, 4506-4510.
83. Hooper, J.W., Marlow, W., Whalley, W.B., Borthwick, A.D., Bowden, R. 1971. The chemistry of fungi. Part LXV. The structures of ergochrysin A, isoergochrysin A, and ergoxanthin, and of secalonic acids A, B, C, and D. Journal of the Chemical Society. 21, 3580-3590.
84. Hosoe, T., Nozawa, K., Kawahara, N., Fukushima, K., Nishimura, K., Miyaji, M., Kawai, K. 1999. Isolation of a new potent cytotoxic pigment along with indigotin from the pathogenic basidiomycetous fungus *Schizophyllum commune*. Mycopathologia. 146, 9-12.
85. Hosseini, T.A., Shariati, M. 2009. *Dunaliella* Biotechnology: methods and applications. Journal of Applied Microbiology. 107, 14-35.
86. Iacobucci, G.A., Sweeney, L.G. 1981. Process for enhancing the sunlight stability of rubrolone. US patent. 4, 285-985.
87. Imamura, N., Adachi, K., Sano, H. 1994. Magnesidin A, a component of marine antibiotic magnesidin, produced by *Vibrio gazogenes* Atcc29988. Journal of Antibiotics. 47, 257-261.
88. Ip, P.F., Chen, F. 2005. Production of astaxanthin by the green microalga *Chlorella zofingiensis* in the dark. Process Biochemistry. 40, 733-738.
89. Isnansetyo, A., Kamei, Y. 2009. Bioactive substances produced by marine isolates of *Pseudomonas*. Journal of Industrial Microbiology and Biotechnology. 36, 1239-1248.
90. Ivanova, E.P., Christen, R., Bizet, C., Clermont, D., Motreff, L., Bouchier, C., Zhukova, N.V., Crawford, R.J., Kiprianova, E.A. 2009. *Pseudomonas brassicacearum* subsp. *neoaurantiaca* subsp. nov., orange pigmented bacteria isolated from soil and the rhizosphere of agricultural plants. International Journal of Systematic and Evolutionary Microbiology. 59, 2476-2481.
91. Jaeger, R.J.R., Spiteller, P. 2010. Mycenaaurin A, an antibacterial polyene pigment from the fruiting bodies of *Mycena aurantiomarginata*. Journal of Natural Products. 73, 1350-1354.
92. Jehlička, J., Edwards, H.G.M., Oren, A. 2014. Raman spectroscopy of microbial pigments. Applied and Environmental Microbiology. 80(11), 3286-3295.

93. Jenkins, C.L., Andrewes, A.G., McQuade, T.J., Starr, M.P. 1979. The pigment of *Pseudomonas paucimobitis* is a carotenoid (Nostoxanthin), rather than a brominated aryl-polyene (Xanthornonadin). Current Microbiology. 3, 1-4.
94. Jiménez, M.M., Bahena, S.M., Espinoza, C., Trigos, A. 2010. Isolation, characterization, and production of red pigment from *Cercospora piaropia* biocontrol agent for waterhyacinth. Mycopathologia. 169, 309-314.
95. Joshi, M.N., Sharma, A.C., Pandya, R.V., et al. 2012. Draft Genome Sequence of *Pontibacter* sp. nov. BAB1700, a halotolerant, industrially important bacterium. Journal of Bacteriology. 194, 6329-6330.
96. Kahng, H.Y., Chung, B.S., Lee, D.H., Jung, J.S., Park, J.H., Jeon, C.O. 2009. *Cellulophaga tyrosinoxydans* sp. nov., a tyrosinase-producing bacterium isolated from seawater. International Journal of Systematic and Evolutionary Microbiology. 59, 654-657.
97. Karki, H.S., Shrestha, B.K., Han, J.W., Groth, D.E., Barphagha, I.K., Rush, M.C., Melanson, R.A., Kim, B.S., Ham, J.H. 2012. Diversities in virulence, antifungal activity, pigmentation and DNA fingerprint among strains of *Burkholderia glumae*. PLoS ONE. 7, e45376.
98. Karuppiah, V., Aarthi, C., Sivakumar, K., Kannan, L. 2013. Statistical optimization and anticancer activity of a red pigment isolated from *Streptomyces* sp. PM4. Asian Pacific Journal of Tropical Biomedicine. 3, 650-656.
99. Kawai, K., Nozawa, Y. 1982. Biochemistry of pigments from pathogenic fund: Chemical structures and biological activities. Japanese Journal of Medical Mycology. 23, 1010-115.
100. Khaneja, R., Perez-Fons, L., Fakhry, S., et al. 2010. Carotenoids found in *Bacillus*. Journal of Applied Microbiology. 108, 1889-1902.
101. Kientz, B., Marie, P., Rosenfeld, E. 2012. Effect of abiotic factors on the unique glitter-like iridescence of *Cellulophaga lytica*. FEMS Microbiology Letters. 333(2), 101-108.
102. Kim, D., Lee, J. S., Park, Y. K., et al. 2007. Biosynthesis of antibiotic prodiginines in the marine bacterium *Hahella chejuensis* KCTC 2396. Journal of Applied Microbiology.102, 937-944.
103. Kleinig, H., Reichenbach, H., Achenbach, H. 1970. Carotenoid Pigments of *Stigmatella aurantiaca* (Myxobacterales). II. Acylated Carotenoid Glucosides. Archives of Microbiology. 74, 223-234.
104. Knaekmuss, H.J. 1973. Chemistry and biochemistry of azaquinones. Angewandte Chemie. 12, 139-145.
105. Knaekmuss, H.J., Beekmann, W. 1973. The Structure of Nicotine Blue from Arthrobacter oxidans. Archives of Microbiology. 90, 167-169.
106. Knight, D.W., Pattenden, G.1979. Syntheses of permethylated derivatives of pinastric acid and gomphidic acid, pulvinic acid pigments of lichen and fungi. Journal of the Chemical Society, Perkin Transactions. 1, 84-88.
107. Kobayashi, H., Nogi, Y., Horikoshi, K. 2007. New violet 3,3′-bipyridyl pigment purified from deep-sea microorganism *Shewanella violacea* DSS12. Extremophiles. 11, 245-250.
108. Kohl, H., Bhat, S.V., Patell, J.R., Ghandhi, N.M., Hazereth, J., Diveker, P.V., de Souza, N.J. 1974. Structure of magnesidin, a new magnesium-containing antibiotic from *Pseudomonas magnesiorubra*. Tetrahedron Letters. 12, 983-986.

109. Korth, H., Romer, A., Budzikiewicz, H., Pulverer, G. 1978. 4,9-Dihydroxyphenazine-l,6-dicarboxylic Acid Dimethylester and the 'Missing Link' in Phenazine Biosynthesis. Journal of General Microbiology. 104, 299-303.
110. Kotob, S.I., Coon, S.L., Quintero, E.J., Weiner, R.M. 1995. Homogentistic acid is the primary precursor of melanin synthesis in *Vibrio cholerae*, a *Hyphomonas* strain, *Shewanella clowelliana*. Applied and Environmental Microbiology. 61, 1620-1622.
111. Kronick, M.N. 1986. The use of phycobiliproteins as fluorescent labels in immunoassay. Journal of Immunological Methods. 92, 1-13.
112. Laatsch, H. 2006. Marine bacterial metabolites, in: Proksch, P., Müller, W. E. G. (eds.), Frontiers in Marine Biotechnology. Horizon Bioscience, Norfolk, U.K., pp. 225-288.
113. Lee, J.H., Kim, Y.-S., Choi, T.-J., Lee, W.J., Kim, Y.T. 2004. *Paracoccus haeundaensis* sp. nov., a gram-negative, halophilic, astaxanthin-producing bacterium. International Journal of Systematic and Evolutionary Microbiology. 54, 1699-1702.
114. Lee, J.P., Yi, C.S., LeGall, J., Peck Jr, H.D. 1973. Isolation of a new pigment, desulforubidin, from *Desulfovibrio desulfuricans* (Norway strain) and its role in sulfite reduction. Journal of Bacteriology. 115, 453-455.
115. Lee, J.S., Kim, Y.S., Park, S., et al. 2011. Exceptional production of both prodigiosin and cycloprodigiosin as major metabolic constituents by a novel marine bacterium, *Zooshikella rubidus* S1-1. Applied and Environmental Microbiology. 77, 4967-4973.
116. Lewis, S.M., Corpe, W.A. 1964. Prodigiosin producing bacteria from marine sources. Applied Microbiology. 12, 13-17.
117. Li, J., Chen, G.H., Wu, H.M., Webster, J.M. 1995. Identification of two pigments and a hydroxystilbene antibiotic from *Photorhabdus luminescens*. Applied and Environmental Microbiology. 61, 4329-4333.
118. Li, Y., Han, L., Rong, H., Li, L., Zhao, L., Wu, L., Xu, L., Jiang, Y., Huang, X. 2014. Diastaphenazine, a new dimeric phenazine from an endophytic *Streptomyces diastaticus* subsp. *ardesiacus*. Journal of Antibiotics. 1-3.
119. Liao, H., Chung, K. 2008. Cellular toxicity of elsinochrome phytotoxins produced by the pathogenic fungus, *Elsinoe fawcettii* causing citrus scab. New Phytologist. 177, 239-250.
120. Logan, N.A. 1989. Numerical taxonomy of violet-pigmented, gram-negative bacteria and description of *Iodobacter fluviatile* gen. nov., comb. nov. International Journal of Systematic Bacteriology. 39, 450-456.
121. Lu, R., Luo, F., Hu, F., Huang, B., Li, C., Bao, G. 2013. Identification and production of a novel natural pigment, cordycepoid A, from *Cordyceps bifusispora*. Applied Microbiology and Biotechnology. 97, 6241-6249.
122. Lu, X., Al-Qadiri, H.M., Lin, M., Rasco, B.A. 2011. Application of mid-infrared and Raman spectroscopy to the study of bacteria. Food and Bioprocess Technology. 4, 919-935.
123. Luesch, H.; Moore, R.E.; Paul, V.J.; Mooberry, S.L.; Corbett, T.H. 2001. Isolation of dolastatin 10 from the marine cyanobacterium *Symploca* species VP642 and total stereochemistry and biological evaluation of its analogue symplostatin 1. J. Nat. Prod. 64, 907–910.
124. Lund, B.M., Brocklehurst, T.F., Wyatt, G.M. 1981. Characterization of Strains of *Clostridium puniceum* sp. nov., a Pink-pigmented, Pectolytic Bacterium. Journal of General Microbiology. 122, 17-26.

125. Madigan, M.T. 1986. *Chromatium tepidum* sp. nov., a thrmophilic photosynthetic bacterium of the family Chromatiaceae. International Journal of Systematic Bacteriology. 36, 222-227.
126. Malik, K., Tokkas, J., Goyal, S. 2012. Microbial Pigments: A review. International Journal of Microbial Resource Technology. 1, 361-365.
127. Marshall, J.H., Wilmoth, G.J. 1981. Pigments of *Staphylococcus aureus*, a series of triterpenoid carotenoids. Journal of Bacteriology. 147, 900-913.
128. Maskey, R.P., Grün-Wollny, I., Fiebig, H.H., Laatsch, H. 2002. Akashins A, B, and C: novel chlorinated indigoglycosides from *Streptomyces* sp. GW 48/1497. Angewandte Chemie. 41, 597-599.
129. Maskey, R.P., Kock, I., Helmke, E., Laatsch, H. 2003. Isolation and structure determination of Phenazostatin D, a new phenazine from a marine actinomycete isolate *Pseudonocardia* sp. B6273. Zeitschrift für Naturforschung. 58B, 692-694.
130. Matsumoto, M., Iwama, D., Arakaki, A., Tanaka, A., Tanaka, T., Miyashita, H., Matsunaga, T. 2011. *Altererythrobacter ishigakiensis* sp. nov., an astaxanthin-producing bacterium isolated from marine sediments. International Journal of Systematic and Evolutionary Microbiology. 61, 2956-2961.
131. Matsuno-Yagi, A., Mukohata, Y. 1977. Two possible roles of bacteriorhodopsin; a comparative study of strains of *Halobacterium halobium* differing in pigmentation. Biochemical and Biophysical Research Communications. 78, 237-243.
132. Medentsev, A.G.; Akimenko, V.K. 1998. Naphthoquinone metabolites of the fungi. Phytochemistry. 47(6), 935--959.
133. Meiler, D., Taylor, A. 1970. The effect of cochliodinol, a metabolite of *Chaetomium cochliodes* on the respiration of microspores of *Fusarium oxysporum*. Canadian Journal of Microbiology. 17, 83-86.
134. Meyer, J.M., Abdallah, M.A. 1978. The Fluorescent Pigment of *Pseudomonas fluorescens*: Biosynthesis, purification and physicochemical properties. Journal of General Microbiology. 107, 319-328.
135. Millie, D.F., Ingram, D.A., Dionigi, C.P. 1990. Pigment and photosynthetic responses of *Oscillatoria agardhii* (Cyanophyta) to photon flux den-sity and spectral quality. Journal of Phycology. 26, 660-666.
136. Montano, G.A., Bowen, B.P., LaBelle, J.T., Woodbury, N.W., Pizziconi, V.B., Blankenship, R.E. 2003. Characterization of *Chlorobium tepidum* Chlorosomes: A Calculation of Bacteriochlorophyll *c* per Chlorosome and Oligomer Modeling. Biophysical Journal. 85, 2560-2565.
137. Moppett, C.E., Dix, D.T., Johnson, F. 1971. Structure of Thermorubin A, the Major Orange-Red Antibiotic of *Thermoactinomyces antibioticus*. Journal of the American Chemical Society. 94, 13269-3272.
138. Moreno, J., Rodriquez, H., Vargas, M.A., Rivas, J., Guerrero, M.G. 1995. Nitrogen fixing cyanobacteria as a source of phycobiliproteins pigments - composition and growth performance of ten filamentous *Herterocystous* strains. Journal of Applied Phycology. 7, 17-23.
139. Moss, M. 2002. Bacterial pigments. Micobiologist. 3, 10-12.

140. MubarakAli, D., Gopinath, V., Rameshbabu, N., Thajuddin, N. 2012. Synthesis and characterization of CdS nanoparticles using C-phycoerythrin from the marine cyanobacteria. Materials Letters. 74, 8-11.
141. Nakamura, L.K. 1989. Taxonomic relationship of black-pigmented *Bacillus subtilis* Strains and a proposal for *Bacillus atrophaeus* sp. nov. International Journal of Systematic Bacteriology. 39, 295-300.
142. Nambou, K., Jian, X., Zhang, X., Wei, L., Lou, J., Madzak, C., Hua, Q. 2015. Flux balance analysis inspired bioprocess upgrading for lycopene production by a metabolically engineered strain of *Yarrowia lipolytica*. Metabolites. 5, 794-813.
143. Nedashkovskaya, O.I., Suzuki, M., Vancanneyt, M., Cleenwerck, I., Lysenko, A.M., Mikhailov, V.V., Swings, J. 2004. *Zobellia amurskyensis* sp. nov., *Zobellia laminariae* sp. nov. and Zobellia russellii sp. nov., novel marine bacteria of the family Flavobacteriaceae. International Journal of Systematic and Evolutionary Microbiology. 54, 1643-1648.
144. Nelis, H.J., de Leenheer, A.P. 1991. Microbial sources of carotenoidpigments used in foods and feeds. Journal of Applied Bacteriology. 70, 181-191.
145. Nelsen, S.F. 2010. Bluing components and other pigments of *Boletes*. Fungi. 3, 11-14.
146. Ng, A.S., Just, G., Blank, F. 1969. Metabolites of pathogenic fungi. VII. On the structure and stereo-chemistry of xanthomegnin, vioxanthin, and viopurpurin pigments from *Trichophyton violaceum*. Canadian Journal of Chemistry. 47, 1223-1227.
147. Norton, C.F., Jones, G.E. 1969. A marine isolate of *Pseudomonas nigrifacience*. II. Characterization of its blue pigment. Archives of Microbiology. 64, 369-376.
148. Nugraheni, S.A., Khoeri, M.M., Kusmita, L., Widyastuti, Y., Radjasa, O.K. 2010. Characterization of carotenoid pigments from bacterial symbionts of seagrass *Thalassia hemprichii*. Journal of Coastal Development. 14, 51-60.
149. Okuno, T., Natsume, I., Sawai, K., Sawamura, K., Furusaki, A., Matsumoto, T. 1983. Structure of antifungal and phytotoxic pigments produced by *Alternaria* species. Tetrahedron Lett. 24, 5653-5656.
150. Olivier, M., Ham, K.V.D., Shio, M.T., Kassa, F.A., Fougeray, S. 2014. Malarial pigment hemozoin and the innate inflammatory response. Frontiers in Immunology. 5, 25.
151. Otani, S., Takatsu, M., Nakano, M., Kasai, S., Miura, R. 1974. Letter: Roseoflavin, a new antimicrobial pigment from *Streptomyces*. Journal of Antibiotics. 27, 86-87.
152. Papaioannou, E.H., Liakopoulou-Kyriakides, M. 2010. Substrate contribution on carotenoids production in *Blakeslea trispora* cultivations. Food and Bioproducts Processing. 8, 305-311.
153. Parisot, D., Devys, M., Barbier, M. 1991. Nectriachrysone, a new metabolite related to fusarubin produced by the fungus *Nectria haematococca*. Journal of the Chemical Society, Perkin Transactions. 1, 2280-2281.
154. Parthasarathy, R., Sathiyabama, M. 2015. Lovastatin-producing endophytic fungus isolated from a medicinal plant *Solanum xanthocarpum*. Natural Product Research. 29(24), 2282-2286.
155. Patel, A., Mishra, S., Ghosh, P.K. 2006. Antioxidant potential of C-phycocyanin isolated from cyanobacterial species *Lyngbya*, *Phormidium* and *Spirulina* spp. Indian Journal of Biochemistry and Biophysics. 43, 25-31.

156. Pathirana, C., P. R. Jensen, and W. Fenical. 1992. Marinone and debromomarinone: antibiotic sesquiterpenoid naphthoquinones of a new structure class from a marine bacterium. Tetrahedron Lett. 33: 7663-7666.
157. Pedras, M.S.C., Taylor, J.L., Morales, V.M. 1995. Phomaligin A and other yellow pigments in *Phoma lingam* and *P. wasabiae*. Phytochemistry. 38, 1215-1222.
158. Peek, M.E., Bhatnagar, A., McCarty, N.A. and Zughaier, S.M. 2012. Pyoverdine, the Major Siderophore in *Pseudomonas aeruginosa*, Evades NGAL Recognition. Interdisciplinary Perspectives on Infectious Diseases. 2012: ID 843509,
159. Peix, A., Berge, O., Rivas, R., Abril, A., Velazquez, E. 2005. *Pseudomonas argentinensis* sp. nov., a novel yellow pigment producing bacterial species, isolated from rhizospheric soil in Cordoba, Argentina. International Journal of Systematic and Evolutionary Microbiology. 55, 1107-1112.
160. Pérez-Fons, L., Fraser, P. D. 2012. Analysis of Diapocarotenoids Found in Pigmented *Bacillus* species, in: José-Luis Barredo (ed.), Microbial Carotenoids from Bacteria and Microalgae: Methods and Protocols, Methods in Molecular Biology. 892, 335-345.
161. Peters, S., Spiteller, P. 2007. Sanguinones A and B, blue pyrroloquinoline alkaloids from the fruiting bodies of the mushroom *Mycena sanguinolenta*. Journal of Natural Products. 70, 1274-1277.
162. Piontek, M., Łuszczynska, K., Lechów, H. 2016. Occurrence of the Toxin-Producing *Aspergillus versicolor* Tiraboschi in residential buildings. International Journal of Environmental Research and Public Health. 13, 862.
163. Polívka, T., Niedzwiedzki, D., Fuciman, M., Sundstrom, V., Frank, H. A. 2007. Role of B800 in carotenoid-bacteriochlorophyll energy and electron transfer in LH2 complexes from the purple bacterium *Rhodobacter sphaeroides*. Journal of Physical Chemistry B. 111, 7422-7431.
164. Popa, G., Cornea, C.P., Luta, G., et al. 2016. Antioxidant and antimicrobial properties of *Laetiporus sulphurous* (Bull.) Murrill. AgroLife Scientific Journal. 5, 168-173.
165. Priatni, S. 2014. Review: Potential production of carotenoids from *Neurospora*. Nusantara Bioscience. 6, 63-68.
166. Priestap, H.A. 1984. New naphthopyrones from *Aspergillus fonsecaeus*. Tetrahedron. 40, 3617-3624.
167. Pumas, C., Peerapornpisal, Y., Vacharapiyasophon, P., Leelapornpisid, P., Boonchum, W., Ishii, M., Khanongnuch, C. 2012. Purification and characterization of a thermostable phycoerythrin from hot spring cyanobacterium *Leptolyngbya* sp. KC45. International Journal of Agriculture and Biology. 14, 121-125.
168. Pusecker, K., Laatsch, H., Helmke, E., Weyland, H. 1997. Dihydrophencomycin methyl ester, a new phenazine derivative from a marine Streptomycete. Journal of Antibiotics. 50, 479-483.
169. Qian, F., An, L., He, X., Han, Q., Li, X. 2006. Antibacterial activity of xantho-oligosaccharide cleaved from xanthan against phytopathogenic *Xanthomonas campestris* pv. *campestris*. Process Biochemistry. 41, 1582-1588.
170. Rai, M., Deshmukh, P., Gade, A., Ingle, A., Kovics, G.J., Irinyi, L. 2009. *Phoma* Saccardo: distribution, secondary metabolite production and biotechnological applications. Critical Reviews in Microbiology. 35, 182-196.

171. Rajagopal, L., Sundari, C.S., Balasubramanian, D., Sonti, R.V. 1997. The bacterial pigment xanthomonadin offers protection against photodamage. FEBS Letters. 415, 125-128.
172. Ramaprasad, E.V.V., Bharti, D., Sasikala, C., Ramana, C.V. 2015. *Zooshikella marina* sp. nov. a cycloprodigiosin-and prodigiosin-producing marine bacterium isolated from beach sand. International Journal of Systematic and Evolutionary Microbiology. 65, 4669-4673.
173. Ramesh, CH., Mohanraju, R. 2015. A review on bioluminescence and its applications. International Journal of Luminescence and Applications. 5, 45-46.
174. Rameshkumar, N., Nair, S. 2009. Isolation and molecular characterization of genetically diverse antagonistic, diazotrophic red-pigmented vibrios from different mangrove rhizospheres. FEMS Microbiology Ecology. 67, 455-467.
175. Rettori, D., Duran, N. 1998. Production, extraction and purification of violacein: an antibiotic pigment produced by *Chromobacterium violaceum*. World Journal of Microbiology and Biotechnology. 14, 685-688.
176. Reverchon, S., Rouanet, C., Expert, D., Nasser, W. 2002. Characterization of indigoidine biosynthetic genes in *Erwinia chrysanthemi* and role of this blue pigment in pathogenicity. Journal of Bacteriology. 184, 654-665.
177. Řezanka, T., Dembitsky, V.M. 2006. Metabolites Produced by Cyanobacteria Belonging to Several Species of the Family Nostocaceae. Folia Microbiologica. 51, 159-182.
178. Rodrigo-Baños, M., Garbayo, I., Vílchez, C., Bonete, M.J., Martínez-Espinosa, R.M. 2015. Carotenoids from *Haloarchaea* and their potential in biotechnology. Marine Drugs. 13, 5508-5532.
179. Romay, C., Gonzalez, R., Ledon, N., Remirez, D., Rimbau, V. 2003. C-Phycocyanin: a biliprotein with antioxidant, anti-Inflammatory and neuroprotective effects. Current Protein & Peptide Science. 4, 207-216.
180. Rosa-Fraile, M. et al. 2006. Granadaene: proposed structure of the group B *Streptococcus* polyenic pigment. Applied and Environmental Microbiology. 72, 6367-6370.
181. Ruzafa, C., Sanchez-Amat A. Solano, F. 1995. Characterization of the Melanogenic System in *Vibrio cholerae*, ATCC 14035, Pigment cell research. 8 (3), 147-152.
182. Saha, S., Thavasi, R., Jayalakshmi, S. 2008. Phenazine pigments from *Pseudomonas aeruginosa* and their application as antibacterial agent and food colourants. Research Journal of Microbiology. 3, 122-128.
183. Sardaryan, E. 2006. Food supplement. United States Patent application 20060247316.
184. Schaeflé, J., Ludwig, B., Albrecht, P., Ourisson, G. 1977. Hydrocarbures aromatique d'origine geologique. II. Nouveaux carotanoïdes aromatiques fossiles. Tetrahedron Letters. 41, 3673-3676.
185. Schumacher, J. 2016. DHN melanin biosynthesis in the plant pathogenic fungus *Botrytis cinereais* based on two developmentally regulatedkey enzyme (PKS)-encoding genes. Molecular Microbiology. 99(4), 729-748.
186. Sekhon, A. S. and Hargesheimer, E. 1975. Sensitivity of some human pathogenic yeasts and systemic fung to myxin. Journal of Clinical Pathology. 28, 547-549.
187. Shaaban, M., Shaaban, K.A., Abdel-Aziz, M.S. 2012. Seven naphtho-γ-pyrones from the marine derived fungus Alternaria alternata: Structure elucidation and biological properties. Organic and Medicinal Chemistry Letters. 2, 6.

188. Shah, M.M.R., Liang, Y., Cheng, J.L., Daroch, M. 2016. Astaxanthin-Producing Green Microalga *Haematococcus pluvialis*: From Single Cell to High Value Commercial Products. Frontiers in Plant Science. 7, 531.
189. Sharma, A., Kaur, J. 2016. Phycocyanin the pigment with a purpose. LAP Lambert Academic Publishing.
190. Sheath, R.G., Wehr, J.D. 2003. Freshwater Algae of North America. Elsevier Science, USA.
191. Shetty, P.R., Buddana, S.K., Tatipamula, V.B., Naga, Y.V.V.N., Ahmad, J. 2014. Production of polypeptide antibiotic from *Streptomyces parvulus* and its antibacterial activity. Brazilian Journal of Microbiology. 45, 303-312.
192. Shieh, W.Y., Chen, Y., Chaw, S., Chiu, H. 2003. *Vibrio ruber* sp. nov., a red, facultatively anaerobic, marine bacterium isolated from sea water. International Journal of Systematic and Evolutionary Microbiology. 53, 479-484.
193. Shindo, K., Endo, M., Miyake, Y., Wakasugi, K., Morritt, D., Bramley, M.P., Fraser, D.P., Kasai, H., Misawa, N. 2008. Methyl glucosyl-3,4-dehydro-apo-8′-lycopenoate, a novel antioxidative glycol-C30-carotenoic acid produced by a marine bacterium *Planococcus maritimus*. Journal of Antibiotics. 61, 729-735.
194. Shmuel, Y. 2004. Dictionary of food compounds with CD-ROM: Additives, flavors, and ingredients. Boca Raton: Chapman & Hall/CRC.
195. Shrishailnath, S., Kulkarni, G., Yaligara, V., Kyoung, L., Karegoudar, T.B. 2010. Purification and physiochemical characterization of melanin pigment from *Klebsiella* sp. GSK. Journal of Microbiology and Biotechnology. 20, 1513-1520.
196. Simon, J., Kroneck, P.M.H. 2013. Microbial Sulfite Respiration, in: Poole, R.K. (ed.), Advances in Microbial Physiology, Volume 62. Academic Press, Elsevier, pp. 45-117
197. Smalley, J. W., et al. 1998. The periodontopathogen *Porphyromonas gingivalis* binds iron protoporphyrin IX in the mu-oxo dimeric form: an oxidative buffer and possible pathogenic mechanism. Biochemical Journal. 331, 681-685.
198. Smânia Jr, A., Marques,C. J. S., Smânia, E.F.A., Zanetti, C.R., Carobrez, S.G., Tramonte, R., Loguercio-Leite, C. 2003. Toxicity and antiviral activity of cinnabarin obtained from *Pycnoporus sanguineus* (Fr.) Murr. Phytotherapy Research. 17, 1069-1072.
199. Solano, F. García, E. Pérez de Egea, E. and Sanchez-Amat, A. 1997. Isolation and Characterization of Strain MMB-1 (CECT 4803), a Novel Melanogenic Marine Bacterium. Appl. Environ. Microbiol. 63(9), 3499-3506.
200. Solano, F and Sanchez-Amat, A. 1999. Studies on the phylogenetic relationships of melanogenic marine bacteria: proposal of *Marinomonas mediterranea* sp. nov. int. J. System. Bacteriol. 49, 1241-1246.
201. Sonani, R.R., Rastogi, R.P., Madamwar, D. 2015. Antioxidant Potential of Phycobiliproteins: Role in Anti-Aging Research. Biochemistry and Analytical Biochemistry. 4, 172.
202. Song, Y.C., Li, H., Ye, Y.H., Shan, C.Y., Yang, Y.M., Tan, R.X. 2004. Endophytic naphthopyrone metabolites are co-inhibitors of xanthine oxidase, SW1116 cell and some microbial growths. FEMS Microbiology Letters. 241, 67-72.
203. Spiteller, P., Arnold, N., Spiteller, M., Steglich, W. 2003. Lilacinone, a red aminobenzoquinone pigment from *Lactarius lilacinus*. Journal of Natural Products. 66, 1402-1403.

204. Stadnichuk, I.N., Romanova, N.I., Selyakh, I.O. 1985. A phycourobilin-containing phycoerythrin from the cyanobacterium *Oscillatoria* sp. Archives of Microbiology. 143, 20-25.
205. Stahmann, K.P., Revuelta, J.L., Seulberger, H. 2000. Three biotechnical processes using *Ashbya gossypii*, *Candida famata*, or *Bacillus subtilis* compete with chemical riboflavin production. Applied Microbiology and Biotechnology. 53, 509-516.
206. Stevenson, C.S., Capper, E.A., Roshak, A.K. 2002. Scytonemin—a marine natural product inhibitor of kinases key in hyperproliferative inflam-matory diseases. Inflammation Research. 51, 112-114.
207. Subramani, R., Kumar, R., Prasad, P., Aalbersberg, W. 2013. Cytotoxic and antibacterial substances against multi-drug resistant pathogens from marine sponge symbiont: Citrinin, a secondary metabolite of *Penicillium* sp. Asian Pacific Journal of Tropical Biomedicine. 3, 291-296.
208. Taber, W.A., Vining, L.C., Sa, W. 1954. Candidin, a new antifungal antibiotic produced by *Streptomyces viridoflavus*. Antibiotics and Chemotherapy. 4, 455-461.
209. Tagua, V.G., Medina, H.R., Martín-Dominguez, R., Eslava, A.P., Corrochano, L.M., Cerdá-Olmedo, E., Idnurm, A. 2012. A gene for carotene cleavage required for pheromone biosynthesis and carotene regulation in the fungus *Phycomyces blakesleeanus*. Fungal Genetics and Biology. 49, 398-404.
210. Takaichi, S., Maoka, T., Yamada, M., Matsuura, K., Haikawa, Y., Hanada, S. 2001a. Absence of carotenes and presence of a tertiary methoxy group in a carotenoid from a thermophilic filamentous photosynthetic bacterium *Roseiflexus castenholzii*. Plant and Cell Physiolology. 42, 1355-1362.
211. Takaichi, S., Jung, D.O., Madigan, M.T. 2001b. Accumulation of unusual carotenoids in the spheroidene pathway, demethylspheroidene and demethylspheroidenone, in an alkaliphilic purple nonsulfur bacterium *Rhodobaca bogoriensis*. Photosynthesis Research. 67, 207-214.
212. Takaichi, S., Maoka, T., Akimoto, N., Sorokin, D.Y., Banciu, H., Kuenen, J. G. 2004. Two novel yellow pigments natronochrome and chloronatronochrome from the natrono(alkali)philic sulfur-oxidizing bacterium *Thialkalivibrio versutus* strain ALJ 15. Tetrahedron Letters. 45, 8303-8305.
213. Takaichi, S., Mochimaru, M., Uchida, H., et al. 2012. Opposite Chilarity of α-Carotene in Unusual Cyanobacteria with Unique Chlorophylls, *Acaryochloris* and *Prochlorococcus*. Plant and Cell Physiolology. 53, 1881-1888.
214. Takemoto, K.; Kamisuki, S.; Chia, P.T.; Kuriyama, I.; Mizushina, Y.; Sugawara, F. 2014. Bioactive Dihydronaphthoquinone Derivatives from *Fusarium solani*. Journal of Natural Products. 77: 1992-1196.
215. Tanskul, S., Khoonchumnan, S., Watanasit, S., Oda, K. 2013. Application of a new red carotenoid pigment-producing bacterium, *Enterobacter* sp. P41, as feed supplement for chicken. African Journal of Biotechnology. 12, 64-69.
216. Thanapipatsiri, A., Claesen, J., Gomez-Escribano, J-P., Bibb, M., Thamchaipenet, A. 2015. A *Streptomyces coelicolor* host for the heterologous expression of Type III polyketide synthase genes. Microb Cell Fact. 14: 145.
217. Tisler, M. 1989. Heterocyclic Quinones, in: advances in heterocyclic chemistry, vol. 45. Academic Press. Inc. pp 37-150.

218. Tomasseli, L., Boldrini, G., Margheri, M.C. 1997. Physiological behaviour of *Arthrospira* (*Spirulina*) *maxima* during acclimation to changes in irradiance. Journal of Applied Phycology. 9, 37-43.
219. Tuli, H.S., Sandhu, S.S., Sharma, A.K. 2014. Pharmacological and therapeutic potential of *Cordyceps* with special reference to Cordycepin. 3Biotech. 4, 1-12.
220. Uesugi, S., Fujisawa, N., Yoshida, J., Watanabe, M., Dan, S., Yamori, T., Shiono, Y., Kimura, K. 2016. Pyrrocidine A, a metabolite of endophytic fungi, has a potent apoptosis-inducing activity against HL60 cells through caspase activation via the Michael addition. Journal of Antibiotics. 69, 133-140.
221. Umadevi, K., Krishnaveni, M. 2013. Antibacterial activity of pigment produced from *Micrococcus luteus* KF532949. International Journal of Chemical and Analytical Science. 4, 149-152.
222. Umezawa, H., Hayano, S., Maeda, K., Ogata, Y., Okami, Y. 1950. On a new antibiotic, griseolutein, produced by streptomyces. The Japanese Medical Journal. 3, 111-117.
223. Variyar, P.S., Chander, R., Venkatachalam, S.R., Bongirwar, D.R. 2002. A new red pigment from an alkalophilic *Micrococcus* species. Indian Journal of Chemistry. 41B, 232-233.
224. Vasanthabharathi, V., Lakshminarayanan, R., Jayalakshmi, S. 2011. Melanin production from marine *Streptomyces*. African Journal of Biotechnology. 10, 11224-11234.
225. Veiga-Crespo, P., Vinuesa, T., Viñas, M., Villa, T.G. 2012. Analysis of Canthaxanthin Production by *Gordonia jacobaea*, in: Barredo, J. (ed.), Microbial Carotenoids from Bacteria and Microalgae: Methods and Protocols, Methods in Molecular Biology, vol. 892. Springer Science+Business Media, LLC, pp. 159-172.
226. Velíšek, J., Cejpek, K. 2011. Pigments of Higher Fungi: A Review. Czech Journal of Food Sciences. 29, 87-102.
227. Venil, C.K., Zakaria, A.Z., Ahmad, W.A. 2015. Optimization of culture conditions for flexirubin production by *Chryseobacterium artocarpi* CECT 8497 using response surface methodology. Acta Biochimica Polonica. 62, 185-190.
228. Vogel, F.S., Kemper, L.A., Jeffs, P.W., Cass, M.W., Graham, D.G. 1977. gamma-L-Glutaminyl-4-hydroxybenzene, an inducer of cryptobiosis in *Agaricus bisporus* and a source of specific metabolic inhibitors for melanogenic cells. Cancer Res. 37(4): 1133-1136.
229. Wada, N., Sakamoto, T., Matsugo, S. 2013. Multiple roles of photosynthetic and sunscreen pigments in cyanobacteria focusing on the oxidative stress. Metabolites. 3, 463-483.
230. Wagner-Döbler, I., Beil, W., Lang, S., Meiners, M., Laatsch, H. 2002. Integrated approach to explore the potential of marine microorganisms for the production of bioactive metabolites. Advances in Biochemical Engineering/Biotechnology. 74, 207- 238.
231. Walter, A., de Carvalho, J. C., Thomaz-Soccol, V., Faria, A.B.B., Ghiggi, V., Soccol, C.R. 2011. Study of phycocyanin production from *Spirulina platensis* under different light spectra. Brazilian Archives of Biology and Technology. 54, 675-682
232. Wang, L.C., Lung, T.Y., Kung, Y.H., Wang, J.J., Tsai, T.Y., Wei, B.L., Pan, T.M., Lee, C.L. 2013. Enhanced anti-obesity activities of red mold dioscorea when fermented using deep ocean water as the culture water. Marine Drugs. 11, 3902-3925.

233. Wang, X., Tan, R., Wang, F., Steglich, W., Liu, J. 2004. The First Isolation of a Phlegmacin Type Pigment from the Ascomycete *Xylaria euglossa*. Zeitschrift für Naturforschung. 60B, 333-336.
234. Wang, Z., O'Shaughnessy, T.J., Soto, C.M., Rahbar, A.M., Robertson, K.L., Lebedev, N., Vora, G.J. 2012. Function and regulation of *Vibrio campbellii* proteorhodopsin: acquired phototrophy in a classical organoheterotroph. PLoS ONE. 7, e38749.
235. Warren, Y.A., Citron, D.M., Merriam, C.V., Goldstein, E.J.C. 2005. Biochemical differentiation and comparison of *Desulfovibrio* species and other phenotypically similar genera. Journal of Clinical Microbiology. 43, 4041-4045.
236. Woo, P.C.Y., et al. 2014. The biosynthetic pathway for a thousand-year-old natural food colorant and citrinin in *Penicillium marneffei*. Scientific Reports. 4, 6728.
237. Yada, S., Wang, Y., Zou, Y., et al. 2008. Isolation and characterization of two groups of novel marine bacteria producing violacein. Marine Biotechnology. 10, 128-132.
238. Yamamoto, C., Takemoto, H., Kuno, K., Yamamoto, D., Tsubura, A., Kamata, K., Hirata, H., Yamamoto, A., kano, H., Seki, T., Inoue, K. 1999. Cycloprodigiosin hydrochloride, a New H^+ /Cl^- symporter, induces apoptosis in human and rat hepatocellular cancer cell linesin vitro and inhibits the growth of hepatocellular carcinoma xenografts in nude mice. Hepatology. 30, 894-902.
239. Yang, X., Qin, C., Wang, F., Dong, Z., Liu, J. 2008. A New Meroterpenoid Pigment from the Basidiomycete *Albatrellus confluens*. Chemistry & Biodiversity. 5, 484-489.
240. Yilmaz, N., Visagie, C.M., Houbraken, J., Frisvad, J.C., Samson, R.A. 2014. Polyphasic taxonomy of the genus *Talaromyces*. Studies in Mycology. 78, 175-341.
241. Yokoyama, A., Adachi, K., Shizuri, Y. 1995. New carotenoid glucosides, astaxanthin glucoside and adonixanthin glucoside, isolated from the astaxanthin-producing marine bacterium, *Agrobacterium aurantiacum*. Journal of Natural Products. 58: 1929-1933.
242. Yoshida, K.; Yoshioka, D.; Inoue, K.; Takaichi, S.; Maeda, I. 2007. Evaluation of colors in green mutants isolated from purple bacteria as a host for colorimetric whole-cell biosensors. Appl Microbiol Biotechnol. 76(5): 1043-1050.
243. Yoshizawa, S., Karatani, H., Wada, M., Kogure, K. 2012. *Vibrio azureus* emits blue-shifted light via an accessory blue fluorescent protein. FEMS Microbiology Letters. 329, 61-68.
244. Zalas, M., Gierczyk, B., Bogacki, H., Schroeder, G. 2015. The *Cortinarius* fungi dyes as sensitizers in dye-sensitized solar cells. International Journal of Photoenergy. 2015, ID 653740.
245. Zhang, X., Enomoto, K. 2011. Characterization of a gene cluster and its putative promoter region for violacein biosynthesis in *Pseudoalteromonas* sp. 520P1. Applied Microbiology and Biotechnology. 90, 1963-1971.
246. Zussman, R.A., Lyon, L., Vicher, E.E. 1960. Melanoid pigment production in a strain of *Trichophyton rubrum*. Journal of Bacteriology. 80, 708-713.
